# A borane-mediated palladium-catalyzed reductive allylic alkylation of α,β-unsaturated carbonyl compounds†

**DOI:** 10.1039/c9sc05970a

**Published:** 2020-01-13

**Authors:** Barry M. Trost, Zhijun Zuo, Johnathan E. Schultz, Nagaraju Anugula, Katherine A. Carr

**Affiliations:** Departmentof Chemistry, Stanford University Stanford CA 94305-5080 USA bmtrost@stanford.edu

## Abstract

The development of the palladium-catalyzed allylic alkylation of *in situ* generated boron enolates *via* tandem 1,4-hydroboration is reported. Investigation of the reaction revealed insights into specific catalyst electronic features as well as a profound leaving group effect that proved crucial for achieving efficient allylic alkylation of ester enolates at room temperature and ultimately a highly preparatively useful synthesis of notoriously challenging acyclic all-carbon quaternary stereocenters. The method demonstrates boron enolates as viable pro-nucleophiles in transition-metal catalyzed allylic alkylation, potentially opening up further transformations outside their traditional use.

## Abstract 2

A palladium-catalyzed allylation of ester-derived boron enolates, which is prepared through 1,4-reduction of α,β-unsaturated ester analogues.

## Graphical abstract

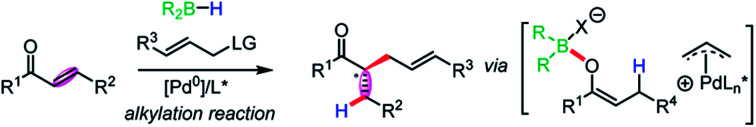

## Introduction

**Scheme 1:**
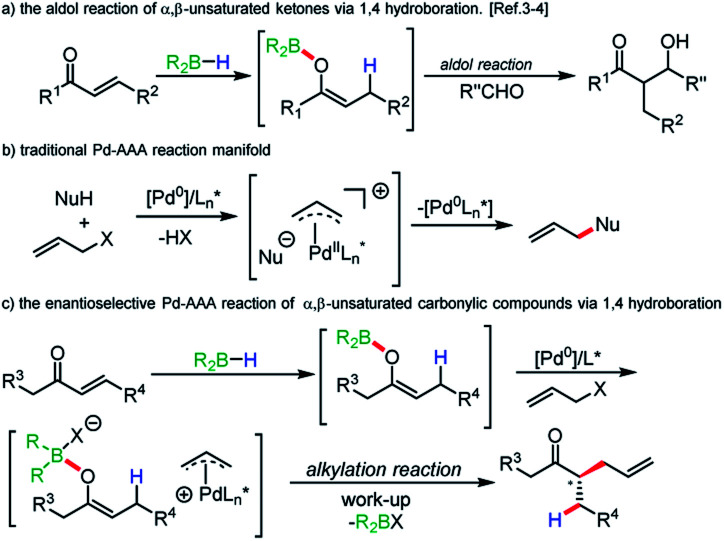
Research background and our goal to regio- and enatioselective reductive allylic alkylation.

Boron enolates have been shown to be of great utility in organic synthesis.^1^ Most noteworthy is the enantio- and diastereoselective aldol reaction. Synthesis of either geometric isomer has been shown to be achievable by judicious choice of base and borane.^2^ A less common method of their generation involves 1,4-hydroboration of α,β-unsaturated carbonyl compounds. First reports of this method using chiral boranes showed low enantioselectivities in subsequent aldol reactions.^3^ Recently, Roush and coworkers have accomplished impressive enantio- and diastereoselectivities in the synthesis of tertiary and quaternary α-carbon centers in aldol reactions when using chiral boranes (Scheme 1a).^4^

Beyond the aldol reaction, few reactions of boron enolates are routinely employed.^5^ Alkylation of boron enolates has been achieved by formation of the “ate” complex followed by treatment with alkyl halides.^6^ In palladium catalysis, a few examples involve forming boron enolates by protonolysis of triethylborane by acidic aldehydes or specific aryl ketones.^7^ Negishi has shown that the potassium enolate of cyclohexanone, when treated with triethylborane, undergoes clean alkylation, although it is unclear if it is the boron “ate” complex, or the potassium enolate that is undergoing alkylation.^8^ More recently, Saegusa oxidation protocols have been developed using enol boranes in analogy to silicon enolates.^9^ Additionally, a dual chiral catalytic rearrangement of allyl esters has been achieved using boron ester enolates and palladium catalysis.^10^

Following our long standing interests in enantioselective Pd-AAA reactions (Scheme 1b),^11^ we became interested in incorporating neutral enol boranes as pronucleophiles in transition metal catalysis, as a means of overcoming limitations imposed by decarboxylative AAA methods.^12^ In particular, the formation of geometrically-controlled tetrasubstituted enol carbonates has remained challenging. Work by Marek and Stoltz has shown that geometrically-selective synthesis of tetrasubstituted amide enolates is achievable by elaboration of ynamides.^13^ However, in these methods, asymmetric α-allylation reactions require the prior synthesis of enol carbonates or enol silyl ethers. It would be advantageous to remove this step to allow for a more direct method of alkylation. Additionally, decarboxylative allylic alkylation of unactivated esters remains a challenge, and asymmetric methods have relied on achiral auxiliaries at the ester oxidation state.^14^

The regioselective allylic alkylation of ketones has only been achieved for ketones differentiated by a bulky substituent. We wished to explore a reactivity strategy that allowed for the direct formation of neutral enol boranes, and to demonstrate their potential for achieving regio- and stereoselectivity for otherwise difficult substrates (Scheme 1c).

In this scenario, 1,4-hydroboration would allow for regioselective generation of enolates that would be inaccessible by acid/base chemistry. Additionally, the thermal 1,4-hydroboration of aliphatic enones is known to give excellent regioselectivity. When employing 1,4-hydroboration, solutions of pure boron enolates could be produced in the absence of ammonium salts produced when reacting dialkylboron chlorides or triflates with ketones in the presence of amine bases.^15^ Certain issues could be anticipated when employing boron enolates in a Pd-catalyzed process. Off-cycle events such as transmetallation of the alkyl group to palladium are possible. Additionally, from the balanced reaction, it can be seen that upon reaction with an activated allyl source, a Lewis-acidic by-product is formed. If activation of the boron enolate by the leaving group is required for reactivity, then the build-up of a Lewis-acid by-product could cause product inhibition, particularly if the by-product is more Lewis-acidic than the starting boron enolate.

## Results and discussion

At the outset, we commenced our efforts by examining the coupling of α,β-unsaturated ketone **1a** and allyl acetate **2a**. A comprehensive ligand screening was undertaken to determine important structural and electronic properties required for reactivity (Table 1). It was observed that Trost ligands performed with little conversion, possibly due to the presence of acidic amide hydrogen atoms. The efficiency of this process appeared to be highly dependent on ligand structure and electronics. Ligands **L11** and **L12** bearing electron-deficient phosphines proved to be differential in reactivity, as the product was formed in higher than 95% conversion for both ligands. Unfortunately, low enantioselectivity was observed for this process.

**Table 1:** Ligand screening for reductive allylic alkylation of enones^a^

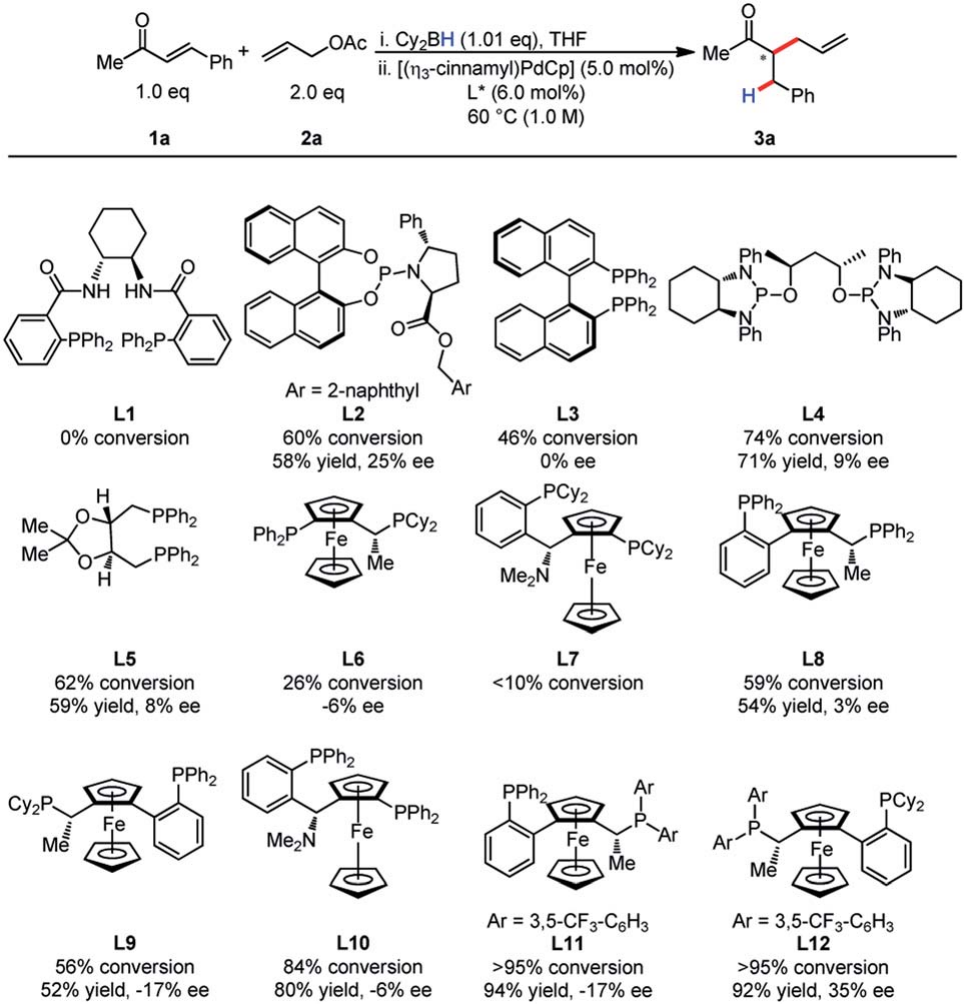

a Reactions were conducted on 0.20 mmol scale.

**Scheme 2:**
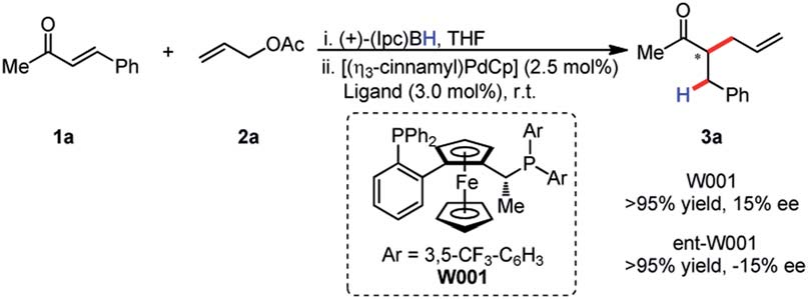
Absence of matched/mis-matched effect with chiral borane.

It was postulated that if the boron atom is involved in the enantio-determining step of this reaction, then the use of a chiral borane could aid in inducing enantioselectivity. When using (+)-Ipc_2_BH and employing both enantiomers of **L11** (W001), neither a matched nor mismatched effect was observed, and the stereoselectivity was determined entirely by the chirality of the ligand (Scheme 2).

**Scheme 3:**
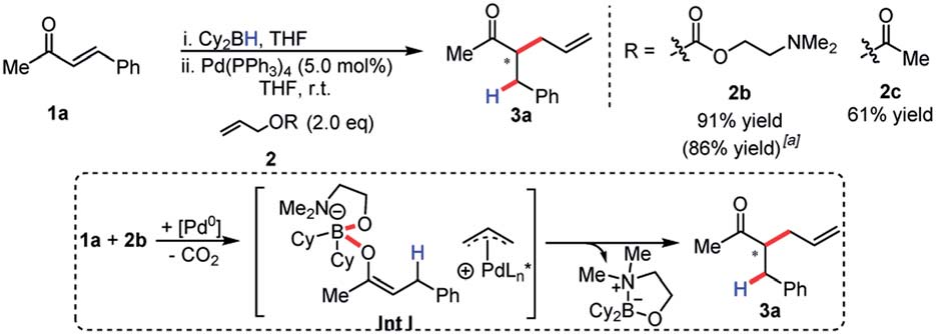
Effect of leaving group on efficiency of ketone alkylation.^a^ Yield for 10.0 mmol scale.

Although enantioselectivity proved elusive in this process, the reactivity remained intriguing, as neutral boron enolates have been used in only a limited way in transition-metal catalyzed enolate allylation. Interestingly, Pd(PPh_3_)_4_ was an active catalyst in the reaction. The reaction was found to display limited conversion when using allyl acetate. It was postulated that the boron Lewis acid could be a source of inhibition in the reaction. Inspired by the work of Hooz employing lithium *N*,*N*-dimethylethanolamine alkoxide in ionic alkylations of boron enolates,^6*a*^ we synthesized a specialized pro-electrophile **2b**, with the aim of sequestering boron (Scheme 3). Gratifyingly, this resulted in excellent yield (91%) in the model reaction comparing with allyl acetate, which presumably is due to the enhanced nucleophilicity of **Int I**. The established reaction proved as a useful starting point for accomplishing high reactivity of ester enolates at room temperature and a preparatively useful method for synthesizing acyclic all-carbon quaternary stereocenters (see below).

**Scheme 4:**
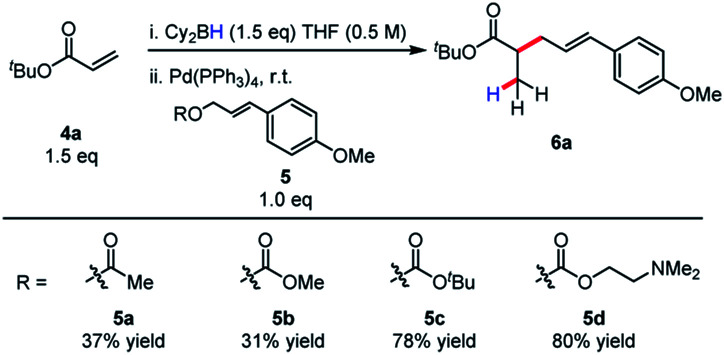
Effect of leaving group on efficiency of reductive allylic alkylation of boryl ketene acetals.

Allylic alkylation of unactivated esters remains a major challenge in Pd-AAA chemistry. In the first demonstration of ester enolate alkylation by Tsuji in 1984, silyl ketene acetals were reacted with allylic carbonates in the presence of Pd(0) and diphenylphosphinoethane (dppe) in refluxing dioxane.^16*a*^ Following this work, room temperature reactivity was established when using diphenylphospinoferrocene (dppf) as ligand.^16*b*^ More recently, Ito^17^ and Tunge^18^ have demonstrated the methods for the decarboxylative allylic alkylation of allyl malonates. Interestingly, the identity of the leaving group also has a strong influence on the conversion of this reaction, with the *tert*-butyl carbonate and the *N*,*N*-dimethylethanolamine groups affording the product in highest yield (Scheme 4). In the case of the *N*,*N*-dimethylethanolamine leaving group, the Lewis-acidic leaving group is sequestered in the process. Additionally, the higher basicity of the *tert*-butyl carbonate leaving group may account for higher reactivity and diminished by-product inhibition. It was discovered in this process that boryl ketene acetals react rapidly at room temperature under the previously discovered reaction conditions.

Invariably, other ester alkylations require aryl esters or electron-withdrawing α-substituents for reactivity to be observed (Table 2). To the best of our knowledge, this is the only case of unactivated ester allylation proceed rapidly at room temperature. In all cases observed, the reaction is complete within minutes.

**Table 2:** A borane-mediated reductive allylic alkylation of acrylates^a^

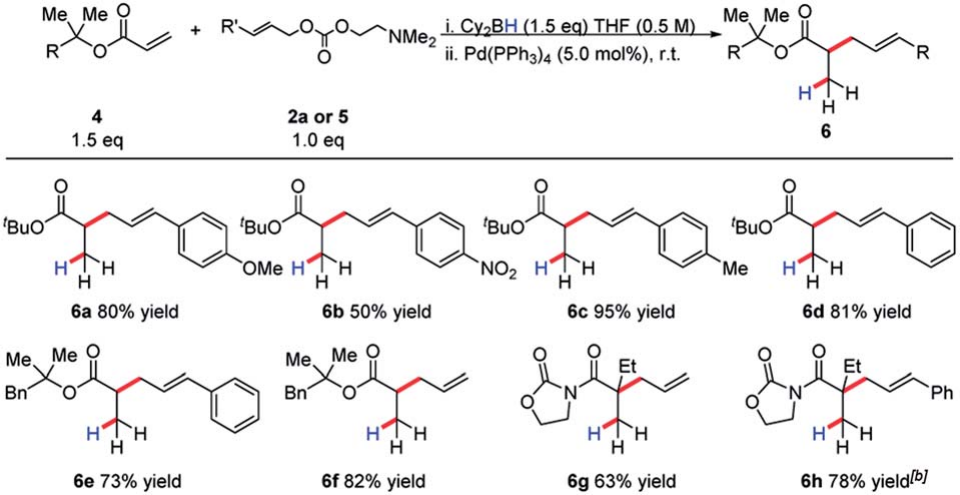

a Reactions were conducted on a range of scales and details see ESI.

b Using the *tert*-butyl carbonate as leaving group.

**Scheme 5:**
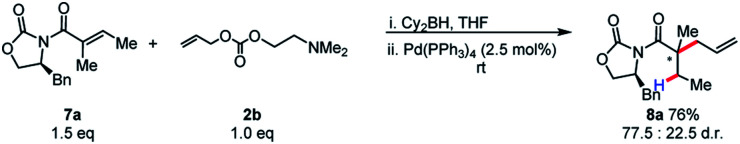
Initial results for chiral auxiliary-controlled diastereoselective allylic alkylation of boryl-ketene aminals.

We became interested in applying this method for the challenging synthesis of acyclic quaternary stereocenters.^19^ Within allylic alkylation methodology, this has been accomplished for a variety of systems,^20^ although alkylation of esters has remained limited. A chiral auxiliary approach was chosen due to the limitations in catalyst-controlled asymmetric induction in the previously described systems. It was envisioned that upon 1,4-hydroboration of a tiglic acid-derived substrate, high diastereoselectivity could be effected by auxiliary-based control. The model system was chosen to demonstrate alkylation at a carbon bearing two sterically similar substituents.^21^ The stereospecificity of the 1,4-hydroboration should prove useful in this context, since the facial differentiation should be controlled by the chiral auxiliary. Our initial screening was with the phenyl alaninol derived auxiliary, giving good yield and modest diastereoselectivity for allylation (Scheme 5).

Because of the 1,3-allylic strain between the α-methyl substituent and the auxiliary, the population of the *S-cis* conformer is likely dependent on the identity of the auxiliary. This potentially explains the lower reactivity for other substrates. Gratifyingly, simply increasing the steric bulk local to the auxiliary by employing phenyl glycinol-derived auxiliary afforded the product in high diastereoselectivity, and this auxiliary was chosen for further studies.

**Scheme 6:**
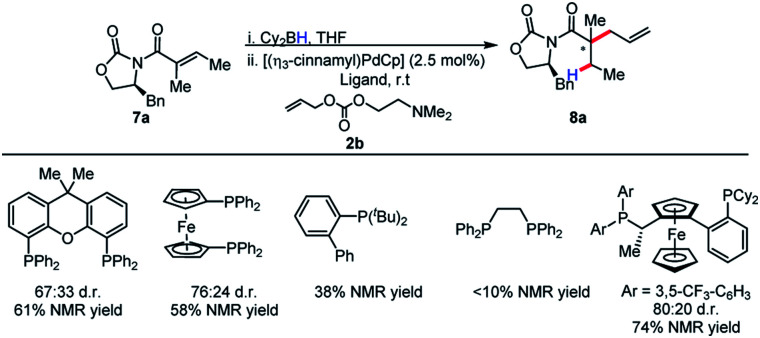
Screening of ligands in auxiliary-controlled allylation.

Although our initial results were with the chiral Walphos ligand, the absence of a pronounced match/mismatch effect led us to reason that the selectivity was solely a result of the auxiliary control. Therefore, a screen of achiral ligands was pursued. Interestingly, achiral ligands afforded the product in diminished reactivities and diastereoselectivities in comparison with chiral Walphos ligand (Scheme 6). Therefore, the chiral Walphos ligand was employed in subsequent studies.

**Scheme 7:**
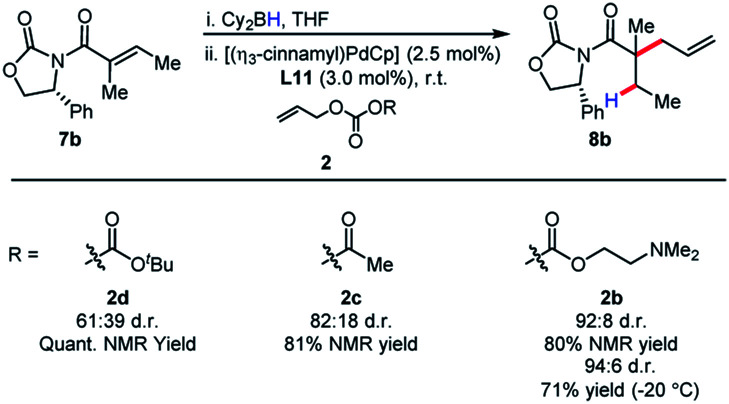
Effect of the leaving group on diastereoselectivity.

**Scheme 8:**
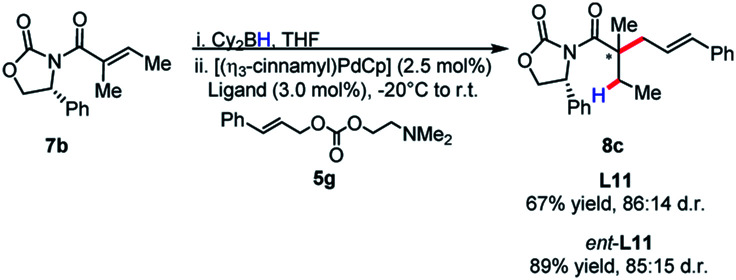
Absence of match/mismatch effect in stereoselectivity of cinnamylation.

The identity of the leaving group had a substantial effect on diastereoselectivity, with the *N*,*N*-dimethylethanolamine carbonate affording the product in the highest diastereoselectivity (Scheme 7). If the reaction proceeds *via* a boron–ate complex, then the dependence of the leaving group on diastereoselectivity would explain these results. The reactions were observed to proceed rapidly, with carbon dioxide extrusion and precipitation of the boron/ethanolamine adduct occurring upon addition of electrophile to the reaction. It was reasoned that beginning the reaction at −20 °C could potentially control any possible exotherm, these observation extended to cinnamylation as shown in Scheme 8.

The substrate scope revealed efficient 1,4-hydroboration and alkylation for substrates bearing a methylene group at the γ-position (Table 3). Branched substitution at this position (R^1^ = Ph, Cy, ^*i*^Pr) proved unreactive in the 1,4-hydroboration process. The sensitivity of the substrate to these structural features can be explained by considering that R^1^ engages with the α-methyl substituent with a considerable amount of strain. This likely causes substituents such as a benzene ring to turn out of conjugation with the π-system, effectively hindering the 1,4-hydroboration event.

**Table 3:** Scope of auxiliary-controlled diastereoselective reductive allylic alkylation for the synthesis of acyclic quaternary stereocenters^a^

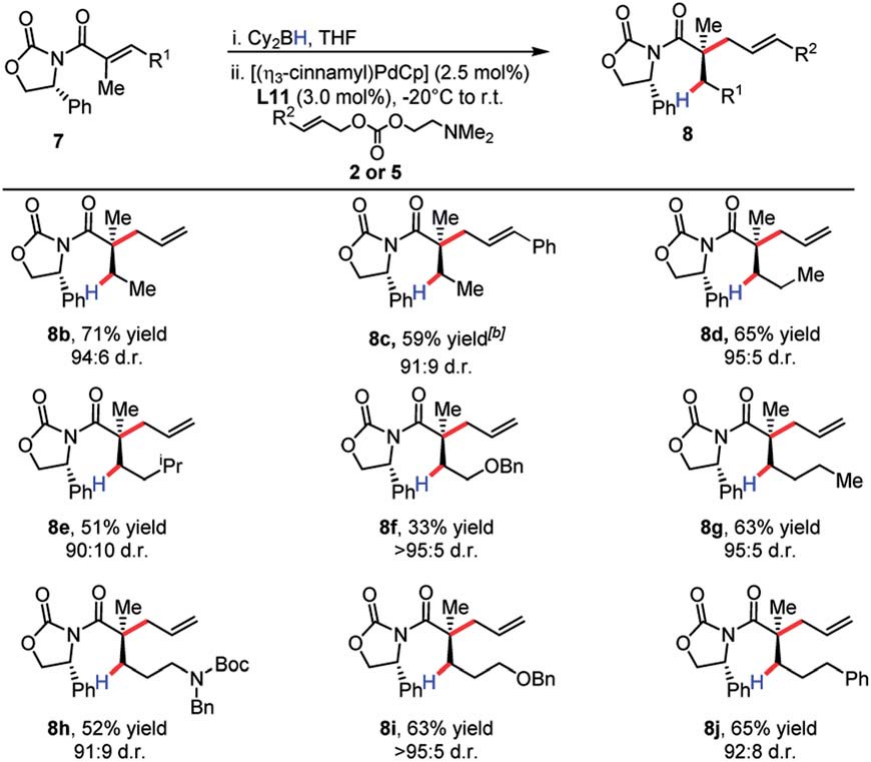

a Reactions were conducted on a range of scales and details see ESI.

b Using Pd(PPh_3_)_4_ as catalyst.

**Scheme 9:**
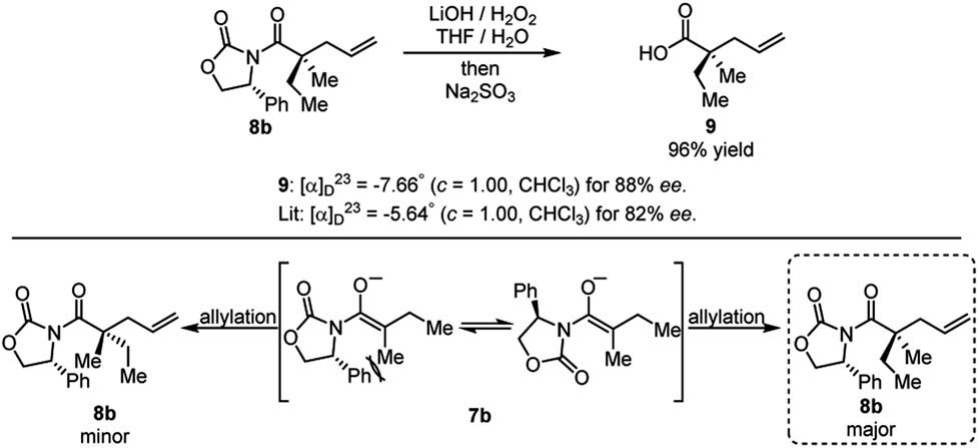
Cleavage of auxiliary and determination of absolute stereochemistry.

The product of the model reaction was converted to corresponding acid, and the absolute stereochemistry was determined by comparison to the optical rotation of the known compound **9** (Scheme 9).^22^ The model for the stereochemical outcome is based on alkylation of an enolate species with the rotamer maximizing dipole cancellation.

The reaction could proceed from the boron “ate” complex that results from leaving group abstraction. In contrast, transmetallation to palladium could conceivably occur prior to alkylation; however, the effect of the leaving group is difficult to rationalize for this reaction pathway, as the boron Lewis acid is not expected to be involved in the diastereo-determining step.

The Lewis acidity of boron enolates explains the higher reactivity in comparison to their silyl counterparts. Abstraction of leaving group upon ionization would be predicted to be much more favorable, leading to a high pre-equilibrium concentration of activated enol borane. The reductive elimination is much more rapid in comparison to reactions of other metal enolate species due to the high effective concentrations afforded by ion-pairing of the active nucleophile and the π-allyl species. Electron-deficient ligands should have a two-fold effect on rate-acceleration: they increase the electrophilicity of the π-allyl species and are less prone to poisoning by the Lewis acidic by-product. This is better understood when recognizing that σ-donation is diminished (ability to act as a Lewis base with boron) and π-back donating ability is increased (*i.e.* higher affinity for palladium in comparison to boron). The effect of leaving group on conversion of ester enolates strongly suggests that the by-product has an inhibitory effect on reaction conversion.

## Conclusions

The reactivity of neutral enol boranes was studied in Pd-allylic alkylation. Important ligand and leaving group effects were discovered that proved important in the development of room temperature ester alkylation, and a preparatively useful diastereoselective alkylation for the synthesis of acyclic quaternary stereocenters bearing sterically similar α-substituents. It is hoped that this work lays the groundwork for understanding the utility of boron enolates in other transition-metal catalyzed processes.

## Conflicts of interest

The authors declare no competing financial interests.

## Supplementary Material

SC-011-C9SC05970A-s001
