# Supplementary material for: A borane-mediated palladium-catalyzed reductive allylic alkylation of α,β-unsaturated carbonyl compounds
Source: Chem Sci. 2020 Jan 13;11(8):2136–40. doi: 10.1039/c9sc05970a (PMC8150111; doi:10.1039/c9sc05970a)
Supplement: SC-011-C9SC05970A-s001 [file SC-011-C9SC05970A-s001.pdf]

Electronic Supplementary Material (ESI) for Chemical Science.
This journal is © The Royal Society of Chemistry 2020

A Borane-Mediated Palladium-Catalyzed Reductive Allylic Alkylation of α,β-Unsaturated Carbonyl Compounds

Supporting information

Barry M. Trost,* Zhijun Zuo,‡ Johnathan E. Schultz,‡ Nagaraju Anugula and Katherine A. Carr

‡These authors contributed equally to this work

Department of Chemistry, Stanford University, Stanford, CA 94305-5080 (USA),

E-mail: bmtrost@stanford.edu

Table of Contents:

A. General information: S2

B. Synthesis of α,β-Unsaturated Substrates S2

C. General procedure for reductive allylic alkylation reactions S8

D. Synthesis of Electrophiles S16

E. Large Scale preparation of 3a: S19

F. Determination of Absolute Stereochemistry S19

G. NMR spectra: S21

A. General information:

Anhydrous solvents were obtained by elution through alumina columns under a nitrogen atmosphere. Dioxane, THF, DCE, triethylamine, and Hunig's base were distilled from conventional stills with proper drying agents. Commercial reagents were used without purification unless stated otherwise. All reactions were run under an atmosphere of argon unless noted. TLC was performed on pre-coated glass plates. Flash silica gel chromatography was performed with silica gel 60, 230-400 mesh and is indicated as "FSGC" in the procedures. ^{1}H-NMR (7.26 ppm for $CHCl_3$ as internal standard) and ^{13}C-NMR (77.5 ppm for $CDCl_3$ as internal standard) were recorded on Varian UI-500 (500 MHz), Varian MERC-400 (400 MHz). Enantiomeric excesses were determined using chiral HPLC analyses on a Thermo Separation Products Spectra Series P-100 or 200 and UV100 (254 nm) using Chiralcel columns (OJ-H, IA, IB, or IC) eluting with the heptane/*i*-propanol mixtures indicated.

Diastereomeric ratios of products were determined by comparing the integrals of the products with the minor diastereomer peaks, most commonly by the protons on the carbons vicinal to the quaternary stereocenter.

Auxiliary-containing substrates were synthesized by coupling of an auxiliary with the corresponding carboxylic acid or acid chloride.

B. Synthesis of α,β-Unsaturated Substrates

2-Methyl-1-phenylpropan-2-yl acrylate (4b)

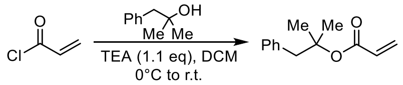

To an ice-cooled, stirring solution of 1-phenyl-2-methyl-2-propanol (7.7 mL, 5.0 mmol, 1.0 eq, *d*=0.974) and trimethylamine (7.7 mL, 55. 0 mmol, 1.1 eq, *d*=0.726) was added acryloyl chloride (4.5 mL, 55.0 mmol, 1.1 eq, *d*=1.114) in a dropwise manner. The reaction was allowed to reach room temperature, and the mixture was removed of most of the solvent. The mixture was suspended in 50.0 mL of diethyl ether and was filtered through a short pad of silica. The silica pad was washed with an additional 50 mL of diethyl ether, and the mixture was removed of solvent. Purification by FSGC (10% diethyl ether in pentane) afforded a clear, colorless oil (2.66 g, 26% yield).

R_f = 0.60 (90 / 10 petroleum ether / EtOAc)

^{1}H NMR (300 MHz, Chloroform-*d*): δ 7.34 – 7.14 (m, 5H), 6.31 (dd, *J* = 17.3, 1.7 Hz, 1H), 6.04 (dd, *J* = 17.3, 10.3 Hz, 1H), 5.73 (dd, *J* = 10.3, 1.7 Hz, 1H), 3.10 (s, 2H), 1.49 (s, 6H).

^{13}C NMR (75 MHz, $CDCl_3$): δ 165.65, 137.17, 130.56, 130.23, 129.54, 127.92, 126.42, 82.39, 46.50, 25.96.

IR (Thin film, NaCl): ν_{max} = 3063, 3031, 2979, 2938, 1720, 1635, 1619, 1496, 1454, 1402, 1385, 1367, 1298, 1257, 1197, 1175, 1121, 1048, 985, 915 cm^{-1}.

HRMS: $C_{13}H_{16}O_2$ [M + Na^+] calc: 227.1048, found: 227.1054.

(*E*)-3-(2-methylbut-2-enoyl)oxazolidin-2-one (4c)

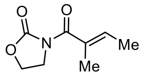

Tiglic acid (2.002 g, 20.0 mmol, 1.0 eq), 2-oxazolidinone (1.742 g, 20.0 mmol, 1.0 eq) and DMAP (0.3665 g, 3.0 mmol, 0.15 eq) were dissolved in DCM (40.0 mL), and EDC•HCl (3.834 g, 20.0 mmol, 1.0 eq) was added in one portion. Triethylamine (2.79 mL, 20.0 mmol, 1.0 eq) was added and the reaction was stirred overnight. The reaction mixture was washed with saturated ammonium chloride, followed by water. The DCM solution was dried over Na_2SO_4, and the crude material was purified by flash silica gel chromatograpy, eluting with 50% ethyl acetate in petroleum ether to afford the product as a white solid (2.12 g, 63% yield).

R_f = 0.07 (80 / 20 petroleum ether / ethyl acetate)

^{1}H NMR (300 MHz, Chloroform-*d*): δ 6.18 (qq, *J* = 6.9, 1.5 Hz, 1H), 4.49 – 4.34 (m, 2H), 4.06 – 3.93 (m, 2H), 1.89 (p, *J* = 1.2 Hz, 3H), 1.79 (dq, *J* = 7.0, 1.2 Hz, 3H).

IR (thin film, NaCl): ν_{max} = 3624, 3547, 3333, 2986, 2922, 1785, 1679, 1478, 1444, 1385, 1347, 1310, 1219, 1116, 1039, 994, 964 cm^{-1}.

Analytical data are in accordance with the literature values.[1]

(*S*,*E*)-4-benzyl-3-(2-methylbut-2-enoyl)oxazolidin-2-one (7a)

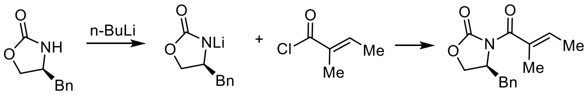

To a stirring solution of (*S*)-4-benzyl-2-oxazolidinone (1.78 g, 10.0 mmol, 1.0 eq) in THF was added n-butyllithium (4.0 mL, 10.0 mmol, 1.0 eq, 2.5 M in hexanes) in a dropwise manner at -78 ℃. After stirring for 30 minutes, tigloyl chloride (1.19 g, 10.0 mmol, 1.0 eq) was added dropwise as a neat liquid. The solution was then allowed to reach room temperature and was quenched by ammonium chloride. The THF was extracted by diethyl ether and the combined organic solvents were dried over magnesium sulfate. The liquid was filtered and evaporated to a crude solid. Purification by flash silica gel chromatography (10% diethyl ether in pentane eluted to 40% diethyl ether in pentane) afforded a fluffy, white solid (1.97 g, 75% yield).

Analytical data are in accordance with the literature values.[2]

R_f = 0.32 (80 / 20 petroleum ether / EtOAc)

^{1}H NMR (400 MHz, Chloroform-*d*): δ 7.30 (dd, *J* = 8.0, 6.3 Hz, 2H), 7.27 – 7.20 (m, 1H), 7.20 – 7.12 (m, 2H), 6.23 – 6.14 (m, 1H), 4.74 – 4.62 (m, 1H), 4.20 (t, *J* = 8.5 Hz, 1H), 4.10 (dd, *J* = 8.9, 5.5 Hz, 1H), 3.30 (dd, *J* = 13.5, 3.5 Hz, 1H), 2.80 (dd, *J* = 13.4, 9.2 Hz, 1H), 1.93 – 1.84 (m, 3H), 1.83 – 1.72 (m, 3H).

^{13}C NMR (100 MHz, $CDCl_3$): δ 171.50, 152.94, 134.95, 134.48, 131.42, 129.21, 128.62, 127.04, 66.14, 55.19, 37.20, 13.87, 13.05.

(*R*,*E*)-3-(2-methylbut-2-enoyl)-4-phenyloxazolidin-2-one (7b)

---

[1] Rana, N.K.; Singh, V.K. *Org. Lett*. **2011**, *13*, 6520-6523.
[2] Dobarro, A.; Velasco, D. *Tetrahedron*, **1996**, *52*, 13525-13530.

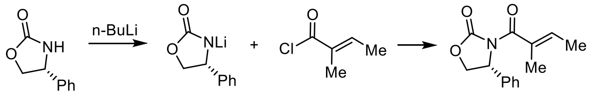

To a solution of (*R*)-(-)-4-phenyl-2-oxazolidinone (3.26 g, 20.0 mmol, 1.0 eq) in THF was added butyl lithium (8.4 mL, 21.0 mmol, 1.05 eq, 2.5 M in hexanes) in a dropwise manner (-78 ℃). The reaction was stirred for 20 minutes, and tigloyl chloride (2.61 mL, 22.0 mmol, 1.1 eq, *d* = 1.00) was added in a single portion. After 10 minutes the reaction was removed from the cooling bath and was allowed to warm to room temperature. The reaction mixture was poured into 40 mL of saturated ammonium chloride solution. The aqueous layer was extracted by ether (2 x 20 mL), and the organic solvent was dried with sodium sulfate, filtered, and evaporated to crude solid. Purification by FSGC (50% diethyl ether: 50% petroleum ether to pure ether) afforded a fluffy white solid (4.3825 g, 89% yield). When this procedure was performed on a 5.96 mmol scale of auxiliary, 1.22 g of product was isolated (83% yield).

R_f = 0.20 (80 / 20 petroleum ether / ethyl acetate)

^{1}H NMR (300 MHz, Chloroform-*d*): δ 7.47 – 7.28 (m, 5H), 6.34 (qq, *J* = 6.9, 1.4 Hz, 1H), 5.48 (dd, *J* = 8.9, 7.6 Hz, 1H), 4.70 (t, *J* = 8.9 Hz, 1H), 4.22 (dd, *J* = 8.9, 7.6 Hz, 1H), 1.87 (t, *J* = 1.2 Hz, 3H), 1.81 (dq, *J* = 7.0, 1.2 Hz, 3H).

IR (thin film, NaCl): ν_{max} = 3034, 2973, 1790, 1683, 1655, 1474, 1458, 1387, 1356, 1327, 1293, 1231, 1207, 1119, 1086, 1061, 963, 942 cm^{-1}.

Analytical data are in accordance with the literature values.[3]

(*R,E*)-3-(2-methylpent-2-enoyl)-4-phenyloxazolidin-2-one (7c)

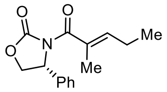

(*E*)-2-methylpent-2-enoic acid (810 mg, 6.3 mmol, 1.0 equiv) was dissolved in 30 mL of THF. The solution was cooled to 0 ℃ and triethylamine (0.87 mL, 6.3 mmol, 1.0 equiv) was added dropwise. To this solution was added pivoyl chloride (0.77 mL, 6.3 mmol, 1.0 equiv) and additional THF. In a separated flask containing (*R*)-4-phenyloxazolidin-2-one (1.02 g, 6.3 mmol, 1.0 equiv) was added 10 mL of THF. The flask was cooled to 0 ℃ and *n*-BuLi (3.15 mL, 6.3 mmol, 1.0 equiv) was added dropwise. The suspension was diluted with an additional 15 mL of THF and was added via syringe to the solution of mixed anhydride cooled in ice water. The reaction mixture was quenched by adding to a stirring biphasic water/diethyl ether mixture. The product was obtained by multiple extractions with diethyl ether. The organic extracts were dried over sodium sulfate, evaporated to dryness, and purified by flash silica gel chromatography eluting with petroleium ether/ethyl acetate (20:1). The product was isolated as white solid. (1.30 g, 80% yield).

R_f = 0.37 (70 / 30 petroleum ether / ethyl acetate)

^{1}H NMR (400 MHz, Chloroform-*d*): δ 7.42 – 7.29 (m, 5H), 6.19 (tq, *J* = 7.4, 1.5 Hz, 1H), 5.46 (dd, *J* = 8.9, 7.4 Hz, 1H), 4.68 (t, *J* = 8.9 Hz, 1H), 4.20 (dd, *J* = 8.9, 7.4 Hz, 1H), 2.27 – 2.12 (m, 2H), 1.86 (s, 3H), 1.04 (t, *J* = 7.6 Hz, 3H).

---

[3] Soloshonok, V.A.; Ueki, H.; Tiwari, R.; Cai, C.; Hruby, V.J. *J. Org. Chem.* **2004**, *69*, 4984-4990.

^{13}C NMR (100 MHz, $CDCl_3$): δ 171.08, 153.38, 142.70, 137.74, 129.83, 129.02, 128.67, 126.17, 69.65, 58.32, 21.72, 13.00, 12.64.

IR (thin film, NaCl): ν_{max} = 3035, 2967, 2933, 2874, 1783, 1685, 1494, 1473, 1459, 1390, 1356, 1329, 1311, 1285, 1230, 1206, 1100, 1086, 1059, 1005, 937, 916 cm^{-1}.

HRMS: $C_{15}H_{17}NO_3$ $[M+H^+]$ calc: 260.1281, found 260.1276.

(*R*,*E*)-3-(2,5-dimethylhex-2-enoyl)-4-phenyloxazolidin-2-one (7d)

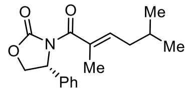

(*E*)-2,5-dimethylhex-2-enoic acid (0.71 g, 5.0 mmol, 1.0 eq) was dissolved in THF (10.0 mL) and was cooled in an ice water bath. Triethylamine (0.70 mL, 5.0 mmol, 1.0 eq, *d* = 0.726) was added dropwise. After stirring for 10 min., pivoyl chloride (0.62 mL, 5.0 mmol, 1.0 eq, *d* = 0.979) was added dropwise. In a separate flask containing (*R*)-(-)-4-phenyl-2-oxazolidinone (0.82 g, 5.0 mmol, 1.0 eq) dissolved in THF (10 mL) was added n-butyllithium (2.0 mL, 5.0 mmol, 1.0 eq, 2.5 M in hexanes) dropwise at 0 ℃. Both solutions were diluted to 25.0 mL of THF to solve solubility issues. The solution of lithiated auxiliary was transferred via syringe to the solution of pre-formed mixed anhydride. The crude reaction mixture was washed with water and extracted with diethyl ether. The crude reaction product was purified by flash silica gel chromatography (gradient of 90:10 PE:EA to pure EA) to afford the product as a white solid (1.20 g, 83% yield).

R_f = 0.33 (80 / 20 petroleum ether / ethyl acetate)

^{1}H NMR (400 MHz, Chloroform-*d*): δ 7.42 – 7.27 (m, 5H), 6.26 (tq, *J* = 7.5, 1.4 Hz, 1H), 5.48 (dd, *J* = 8.9, 7.6 Hz, 1H), 4.70 (td, *J* = 8.9, 0.5 Hz, 1H), 4.23 (ddd, *J* = 8.9, 7.5, 0.5 Hz, 1H), 2.14 – 2.01 (m, 2H), 1.86 (dd, *J* = 1.4, 0.9 Hz, 3H), 1.77 (dp, *J* = 13.4, 6.7 Hz, 1H), 0.94 (dd, *J* = 6.6, 4.7 Hz, 6H).

^{13}C NMR (100 MHz, $CDCl_3$) δ: 171.16, 153.45, 140.73, 137.80, 131.07, 129.14, 128.80, 126.26, 69.73, 58.44, 37.56, 28.31, 22.48, 13.34.

IR (thin film, NaCl): ν_{max} = 2958, 2931, 2871, 1789, 1685, 1652, 1459, 1389, 1356, 1329, 1294, 1232, 1208, 1100, 1085, 1061, 1046, 1004, 911 cm^{-1}.

HRMS: $C_{17}H_{21}NO_3$ $[M+H^+]$ calc: 288.1600, found: 288.1600.

(*R*,*E*)-3-(4-(benzyloxy)-2-methylbut-2-enoyl)-4-phenyloxazolidin-2-one (7e)

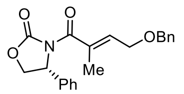

(*E*)-4-(benzyloxy)-2-methylbut-2-enoic acid (2.06 g, 10.0 mmol) was dissolved in 80 mL of dry THF under a nitrogen atmosphere. Triethylamine (1.4 mL, 10.0 mmol) was added at 0 ℃. To this reaction mixture, pivoyl chloride (120.8 mg, 10 mmol) was added in dropwise. In another round bottom flask was dissolved (*R*)-4-methyloxazolidin-2-one (1.63 g, 10.0 mmol) in 80 mL of dry THF under a nitrogen atmosphere. A solution of n-butyllithium (3.74 mL, 10.0 mmol, 2.67 M in hexanes) was added in dropwise. The reaction mixture was stirred for 20 minutes. The resulting solution was added to former reaction mixture in a dropwise manner for about 10 minutes at 0 ℃. The reaction mixture was allowed to warm to r.t. and was

stirred overnight. After completion of reaction, the solvent was evaporated under vacuum. Saturated ammonium chloride solution was added followed by water. The mixture was extracted with ethyl acetate, washed with brine, dried over Na_2SO_4, concentrated, and purified by flash silica gel column chromatography eluting with 50 % dichloromethane in petroleum ether. The product was recrystallized in acetonitrile to afford 1.34 g of white crystalline solid product (38% yield). melting point 118-120 ℃.

R_f = 0.4 (20 / 80 ethyl acetate / petroleum ether)

^{1}H NMR (400 MHz, Chloroform-*d*): δ 7.41 – 7.29 (m, 10H), 6.31 – 6.20 (m, 1H), 5.47 (dd, *J* = 8.8, 7.1 Hz, 1H), 4.71 (t, *J* = 8.9 Hz, 1H), 4.55 (d, *J* = 2.5 Hz, 2H), 4.29 – 4.14 (m, 3H), 1.86 (q, *J* = 1.1 Hz, 3H).

^{13}C NMR (100 MHz, $CDCl_3$): δ 170.6, 153.5, 138.0, 137.9, 136.1, 132.7, 129.4, 129.1, 128.6, 128.2, 128.0, 126.5, 72.6, 70.1, 66.4, 58.5, 13.9.

IR (thin film, NaCl): ν_{max} = 2916, 1783, 1686, 1386, 1205, 1100, 1042, 698 cm^{-1}.

Analytical data are in accordance with the literature values.[4]

(*R*,*E*)-3-(2-methylhex-2-enoyl)-4-phenyloxazolidin-2-one (7f)

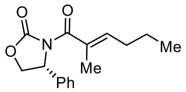

(*E*)-2-methylhex-2-enoic acid (810 mg, 6.3 mmol, 1.0 equiv) was dissolved in 30 mL of THF. The solution was cooled to 0 ℃ and triethylamine (0.87 mL, 6.3 mmol, 1.0 equiv) was added dropwise. To this solution was added pivoyl chloride (0.77 mL, 6.3 mmol, 1.0 equiv) and additional THF. In a separate flask containing (*R*)-4-phenyloxazolidin-2-one (1.02 g, 6.3 mmol, 1.0 equiv) was added 10 mL of THF. The flask was cooled to 0 ℃ and *n*-butyllithium (3.15 mL, 6.3 mmol, 1.0 equiv) was added dropwise. The suspension was diluted with an additional 15 mL of THF and was added via syringe to the solution of mixed anhydride cooled in ice water. The reaction mixture was quenched by adding to a stirring biphasic water/diethyl ether mixture. The product was obtained by multiple extractions with diethyl ether. The organic extracts were dried over sodium sulfate, evaporated to dryness, and purified by flash silica gel chromatography eluting with petroleium ether/ethyl acetate (20:1). The product was isolated as white solid. (1.50 g, 87% yield).

R_f = 0.20 (80 / 20 petroleum ether / ethyl acetate)

^{1}H NMR (500 MHz, Chloroform-*d*): δ 7.39 – 7.28 (m, 5H), 6.22 (tq, *J* = 7.4, 1.5 Hz, 1H), 5.45 (dd, *J* = 8.9, 7.4 Hz, 1H), 4.65 (t, *J* = 8.9 Hz, 1H), 4.17 (dd, *J* = 8.9, 7.4 Hz, 1H), 2.22 – 2.09 (m, 2H), 1.86 (d, *J* = 1.4 Hz, 3H), 1.46 (p, *J* = 7.4 Hz, 2H), 0.93 (t, *J* = 7.4 Hz, 3H).

^{13}C NMR (126 MHz, $CDCl_3$): δ 170.92, 153.28, 141.08, 137.72, 130.43, 128.88, 128.51, 126.06, 69.54, 58.20, 30.25, 21.46, 13.61, 13.03.

IR (thin film, NaCl): ν_{max} = 3351, 3057, 3035, 3010, 2950, 2933, 2863, 1793, 1771, 1682, 1494, 1460, 1389, 1357, 1329, 1293, 1232, 1255, 1206, 1100, 1087, 1061, 999, 967, 910 cm^{-1}.

HRMS: $C_{16}H_{19}NO_3$ $[M+H^+]$ calc: 274.1438, found 274.1432.

[4] Yamada, T.; Sakaguchi, K.; Shinada, T.; Ohfune, Y.; Soloshonok, V.A. *Tetrahedron Asymmetry*, **2008**, *19*, 2789-2795.

***Tert*-butyl (*R,E*)-benzyl(4-methyl-5-oxo-5-(2-oxo-4-phenyloxazolidin-3-yl)pent-3-en-1-yl)carbamate (7g)**

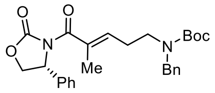

(*E*)-5-(benzyl(tert-butoxycarbonyl)amino)-2-methylpent-2-enoic acid (1.28 g, 4.0 mmol) was dissolved in 40 mL of dry THF under nitrogen atmosphere. Triethylamine (0.56 mL, 4.0 mmol) was added at 0 ℃. Pivoyl chloride (0.5 mL, 4.0 mmol) was added in dropwise manner. In another round bottom flask was dissolved (*R*)-4-methyloxazolidin-2-one (652.6 mg, 4 mmol) in 40 mL of dry THF under a nitrogen atmosphere. A solution of *n*-BuLi (1.5 mL, 4.0 mmol, 2.67 M in hexanes) was added dropwise. The reaction mixture was stirred for 5 minutes. The resulting solution was added to the former reaction mixture in a dropwise manner for about 10 minutes at 0 ℃. The reaction mixture was allowed to warm to r.t. and was stirred overnight. After completion of the reaction, the solvent was evaporated under vacuum. Water was added, and the reaction mixture was extracted with ethyl acetate, washed with brine, concentrated, and purified by flash silica gel column chromatography eluting with 10 % ethyl acetate in petroleum ether to afford 1.07 g of white solid product in 58% yield. melting point 95-97 ℃.

$\mathbf{R_f}$ = 0.50 (30 / 70 ethyl acetate / petroleum ether)

^{1}H NMR (400 MHz, Benzene-d_6): δ7.43 – 7.26 (m, 8H), 7.26 – 7.16 (m, 2H), 6.36 (s, 1H), 5.15 (dd, *J* = 8.8, 6.7 Hz, 1H), 4.58 (s, 2H), 3.96 (t, *J* = 8.8 Hz, 1H), 3.75 (dd, *J* = 8.8, 6.7 Hz, 1H), 3.44 (dt, *J* = 10.7, 4.6 Hz, 2H), 2.53 – 2.36 (m, 2H), 2.06 – 2.02 (m, 3H), 1.64 (s, 9H).

^{13}C NMR (75 MHz, $CDCl_3$) δ: 170.75, 153.29, 137.72, 129.14, 128.80, 128.68, 128.46, 127.71, 127.68, 127.63, 127.52, 127.13, 126.20, 125.88, 79.86, 69.75, 58.31, 28.39, 13.23.

IR (thin film, NaCl): ν_{max} = 3064, 3032, 2976, 2929, 1786, 1691, 1495, 1456, 1414, 1387, 1366, 1297, 1204, 1160, 1122, 1081, 1044, 969, 924 cm^{-1}.

HRMS: $C_{27}H_{32}N_2O_5$ [M+H$^+$] calc: 465.2384, found 465.2379.

(*R,E*)-3-(5-(benzyloxy)-2-methylpent-2-enoyl)-4-phenyloxazolidin-2-one (7h)

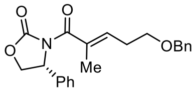

(*E*)-5-(benzyloxy)-2-methylpent-2-enoic acid (220.2 mg, 1.0 mmol) was dissolved in 10 mL of dry THF under nitrogen atmosphere. Triethylamine (0.14 mL, 1.0 mmol) was added at 0 ℃. To this reaction mixture was added (0.12 mL, 1.0 mmol) of trimethylacetyl chloride in a dropwise manner. In another round bottom flask was dissolved (163.2 mg, 1.0 mmol) (*R*)-4-methyloxazolidin-2-one in 10 mL of dry THF under nitrogen atmosphere. To this flask was added *n*-BuLi (0.4 mL, 1.0 mmol, 2.5 M in hexanes) in dropwise manner. The reaction mixture was stirred for 5 minutes. The resulting solution was added to the former reaction mixture in a dropwise manner for 10 minutes at 0 ℃. The reaction mixture was allowed to stir overnight after slowly warming to r.t. After completion of the reaction, the solvent was evaporated under vacuum. Water was added, and the reaction mixture was extracted with ethyl acetate, washed with brine,

concentrated and purified by flash silica gel column chromatography by using 30% ethyl acetate in petroleum ether to afford (182 mg, 50% yield) of white solid product, melting point 92-94 ℃.

R$_f$ = 0.55 (30 % ethyl acetate in petroleum ether);

^{1}H NMR (400 MHz, Chloroform-*d*): δ7.38 – 7.28 (m, 10H), 6.25 (td, *J* = 7.2, 1.4 Hz, 1H), 5.47 (dd, *J* = 8.8, 7.3 Hz, 1H), 4.69 (t, *J* = 8.9 Hz, 1H), 4.52 (s, 2H), 4.22 (dd, *J* = 8.9, 7.3 Hz, 1H), 3.58 (t, *J* = 6.8 Hz, 2H), 2.60 – 2.43 (m, 2H), 1.89 (d, *J* = 1.3 Hz, 3H).

^{13}C NMR (100 MHz, $CDCl_3$): δ171.11, 153.62, 138.53, 138.07, 137.18, 132.32, 129.43, 129.01, 128.63, 127.99, 127.84, 126.55, 73.20, 70.01, 68.63, 58.64, 29.47, 13.69.

IR (thin film, NaCl): ν_{max} = 2909, 2855, 1792, 1681, 1288, 1207, 1097 cm^{-1}.

Analytical data are in accordance with the literature values.[5]

(*R*,*E*)-3-(2-methyl-5-phenylpent-2-enoyl)-4-phenyloxazolidin-2-one (7i)

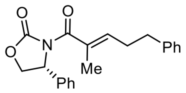

To a r.t. solution of (*R*)-(-)-4-phenyl-2-oxazolidinone (2.38 g, 14.6 mmol, 1.0 eq) in THF (100 mL) was added *n*-BuLi (5.90 mL, 14.7 mmol, 1.01 eq, 2.5 M in hexanes) dropwise. After 30 min., neat acid chloride (3.05 g, 14.6 mmol, 1.0 eq) was added. The solution was then removed of solvent, packed on silica gel, and purified by flash silica gel chromatography (gradient of 90:10 PE:EA to pure EA) to afford the product as a solid (2.69 g, 55% yield).

R$_f$ = 0.23 (80 / 20 petroleum ether / ethyl acetate)

^{1}H NMR (400 MHz, Chloroform-*d*): δ 7.44 – 7.25 (m, 7H), 7.25 – 7.16 (m, 3H), 6.31 – 6.21 (m, 1H), 5.47 (dd, *J* = 8.9, 7.4 Hz, 1H), 4.69 (t, *J* = 8.9 Hz, 1H), 4.22 (dd, *J* = 8.9, 7.4 Hz, 1H), 2.78 (t, *J* = 7.8 Hz, 2H), 2.58 – 2.47 (m, 2H), 1.85 (s, 3H).

^{13}C NMR (100 MHz, $CDCl_3$): δ 170.91, 153.39, 141.06, 139.85, 137.70, 131.00, 129.07, 128.73, 128.33, 126.17, 125.97, 69.69, 58.30, 34.27, 30.24, 13.16.

IR (thin film, NaCl): ν_{max} = 3060, 3028, 2923, 2859, 1787, 1685, 1495, 1475, 1455, 1387, 1359, 1294, 1233, 1206, 1107, 1085, 1061, 1044, 1005, 927 cm^{-1}.

HRMS: $C_{21}H_{21}NO_3$ [M+H$^+$] calc: 336.1594, found: 336.1587.

C. General procedure of reductive allylic alkylation reactions

Procedure A:

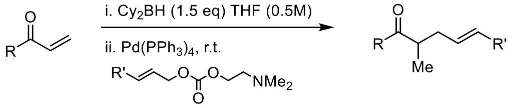

Dicyclohexylborane (1.5 eq) was obtained from the glovebox by addition to an oven-dried microwave vial that was cooled in the vacuum chamber of the glovebox. The solid was suspended in freshly distilled THF, and the corresponding acrylate (1.5 eq) was added dropwise while cooling on an ice bath. After addition, ice was removed from the water/ice mixture, and the water was allowed to warm to r.t. Upon warming to

[5] Inui, M.; Hosokawa, S.; Nakazaki, A.; Kobayshi, S. *Tet. Lett.* **2005**, *46*, 3245-3248.

r.t., a clear solution was obtained. The hydroboration was allowed to stir for an additional 30 minutes to ensure complete hydroboration. Solid $Pd(PPh_3)_4$ (0.05 eq) was quickly added by removing the septum with a stream of argon above the solution. After replacement of the septum, a stream of argon was passed over the solution with a needle as an outlet. Cinnamyl *N,N*-dimethyl ethanolamine carbonate (1.0 eq) was added dropwise. Precipitate was observed within minutes. The reaction was allowed to react for 1 h. to ensure complete conversion. The solution was diluted with 90%/10% diethyl ether/pentane solution and passed through a short plug of silica. The solvent was removed under rotary evaporation, and the oil was purified by flash silica gel chromatography (90/10 diethyl ether/pentane) to afford the products.

Procedure B:

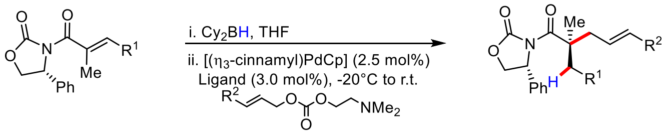

The standard procedure was employed using auxiliary substrate (1.0 eq), dicyclohexylborane (1.1 eq), CpPdcinnamyl (0.02 eq), W001-1 (0.02 eq), and allylic carbonate (1.5 eq) in dry THF. This solution was added to solid dicyclohexylborane at r.t. After 1 hr. of stirring, the solution was gradually heated to 40 ℃, and the solution turned clear after 20 min. The solution was allowed to cool to r.t., and was then cooled on a salt/ice bath (-20 ℃). Catalyst solution was generated in THF was added to the solution of boron enolate. The reaction mixture was allowed to equilibrate to temperature, and allylic carbon was added dropwise over 5 min. The solution was allowed to warm overnight, and the product was purified by flash silica gel chromatography to afford the product.

***tert*-butyl (*E*)-5-(4-methoxyphenyl)-2-methylpent-4-enoate (6a)**

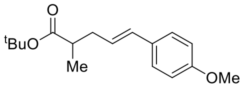

a clear oil (44.2 mg, 80% yield) for 0.20 mmol scale.

$\mathbf{R_f}$ = 0.39 (90 / 10 petroleum ether / ethyl acetate)

^{1}H NMR (400 MHz, Chloroform-*d*): δ 7.29 – 7.21 (m, 2H), 6.88 – 6.78 (m, 2H), 6.35 (d, *J* = 15.7 Hz, 1H), 6.01 (dtd, *J* = 15.4, 6.9, 1.3 Hz, 1H), 3.83 – 3.74 (s, 3H), 2.55 – 2.42 (m, 2H), 2.33 – 2.23 (m, 1H), 1.42 (d, *J* = 4.4 Hz, 9H), 1.17 – 1.10 (m, 3H).

^{13}C NMR (100 MHz, $CDCl_3$): δ 175.48, 158.70, 131.09, 130.25, 127.06, 125.27, 113.81, 79.96, 55.19, 40.55, 37.24, 28.05, 16.71.

IR (thin film, NaCl): ν_{max} = 2976, 2934, 2837, 1727, 1608, 1578, 1512, 1459, 1391, 1367, 1287, 1249, 1153, 1037, 967 cm^{-1}.

HRMS: $C_{17}H_{20}O_3$ $[M+H^+]$ calc: 277.1798 found 277.1805

***tert*-butyl (*E*)-2-methyl-5-(4-nitrophenyl)pent-4-enoate (6b)**

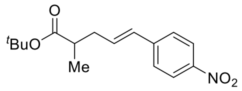

as an orange oil (29.3 mg, 50% yield) for 0.20 mmol scale.

R_f = 0.66 (90 : 10 petroleum ether : EtOAc)

^{1}H NMR (400 MHz, Chloroform-*d*) δ: 8.18 – 8.11 (m, 2H), 7.48 – 7.41 (m, 2H), 6.48 (d, *J* = 16.0 Hz, 1H), 6.42 – 6.31 (m, 1H), 2.60 – 2.45 (m, 2H), 2.41 – 2.30 (m, 1H), 1.45 – 1.41 (m, 9H), 1.17 (d, *J* = 6.8, 3H).

^{13}C NMR (101 MHz, $CDCl_3$) δ: 175.01, 146.81, 143.87, 132.99, 129.98, 129.39, 126.44, 123.98, 80.33, 40.19, 37.27, 28.06, 16.93.

IR (thin film, NaCl): ν_{max} = 2976, 2934, 2874, 1727, 1597, 1519, 1457, 1367, 1343, 1255, 1224, 1153, 1110, 970 cm^{-1}.

HRMS: $C_{16}H_{21}NO_4$ [M+H$^+$] calc: 292.1549, found 292.1544.

***tert*-butyl (*E*)-2-methyl-5-(p-tolyl)pent-4-enoate (6c)**

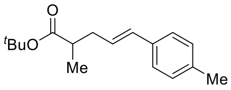

a light yellow oil (49.3 mg, 95% yield) for 0.20 mmol scale.

R_f = 0.79 (90 : 10 petroleum ether : EtOAc)

^{1}H NMR (400 MHz, Chloroform-*d*): δ 7.25 – 7.21 (m, 2H), 7.13 – 7.07 (m, 2H), 6.39 (d, *J* = 15.7 Hz, 1H), 6.16 – 6.05 (m, 1H), 2.56 – 2.43 (m, 2H), 2.33 (s, 3H), 2.32 – 2.25 (m, 1H), 1.41 (s, 9H), 1.16 (d, *J* = 6.8 Hz, 3H).

^{13}C NMR (100 MHz, $CDCl_3$): δ 175.46, 136.72, 134.67, 131.59, 129.13, 126.47, 125.88, 80.00, 40.51, 37.25, 28.07, 21.12, 16.74.

IR (thin film, NaCl): ν_{max} = 3441, 2976, 2935, 2878, 1773, 1727, 1609, 1514, 1456, 1368, 1254, 1155, 1020, 968, 932 cm^{-1}.

HRMS: $C_{17}H_{24}O_2$ [M$^+$] calc: 260.1776, found: 260.1771.

***tert*-Butyl (E)-2-methyl-5-phenylpent-4-enoate (6d)**

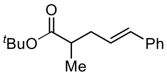

a clear, colorless oil (39.9 mg, 81% yield) for 0.20 mmol scale.

R_f = 0.48 (90/10 petroleum ether/ethyl acetate)

^{1}H NMR (400 MHz, Chloroform-*d*) δ: 7.46 (d, 1H), 7.21-7.39 (m, 5H), 6.15-6.25 (m, 1H), 2.51-2.61 (m, 2H), 2.29-2.42 (m, 1H), 1.56-1.39 (s, 9H), 1.27-1.14 (d, 3H).

^{13}C NMR (100 MHz, $CDCl_3$): δ 175.42, 137.45, 131.76, 128.45, 127.56, 127.02, 125.99, 80.06, 40.47, 37.24, 28.08, 16.77.

IR (thin film, NaCl): ν_{max} = 3081, 3027, 2976, 2932, 1728, 1456, 1391, 1367, 1253, 1221, 1152, 1031, 966 cm^{-1}.

HRMS: $C_{16}H_{22}O_2$ [H$^+$] calc: 247.1693, found 247.1699.

2-methyl-1-phenylpropan-2-yl (*E*)-2-methyl-5-phenylpent-4-enoate (6e)

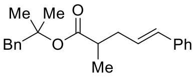

as a clear, colorless oil (235.4 mg, 73% yield) for 1.0 mmol scale.

R_f = 0.45 (90 / 10 petroleum ether / ethyl acetate)

^{1}H NMR (400 MHz, Chloroform-*d*): δ 7.36 – 7.31 (m, 5H), 7.31 – 7.26 (m, 2H), 7.26 – 7.21 (m, 3H), 6.38 (dt, *J* = 15.9, 1.3 Hz, 1H), 6.17 (dt, *J* = 15.8, 7.0 Hz, 1H), 3.14 – 3.04 (m, 2H), 2.61 – 2.49 (m, 2H), 2.40 – 2.30 (m, 1H), 1.52 – 1.47 (m, 6H), 1.21 (d, *J* = 6.6 Hz, 3H).

^{13}C NMR (101 MHz, $CDCl_3$): δ 175.35, 137.31, 137.13, 131.79, 130.62, 128.38, 127.80, 127.32, 126.97, 126.37, 125.96, 81.78, 46.80, 40.57, 37.12, 25.84, 25.81, 16.78.

IR (thin film, NaCl): ν_{max} = 3083, 3061, 3028, 2975, 2933, 2877, 2850, 1948, 1880, 1802, 1726, 1600, 1494, 1454, 1383, 1367, 1329, 1280, 1268, 1169, 1119, 1074, 1032, 966, 915 cm^{-1}.

HR-MS: $C_{22}H_{26}O_2$ $[M+Na^+]$ calc: 345.1830, found: 345.1826.

2-methyl-1-phenylpropan-2-yl 2-methylpent-4-enoate (6f)

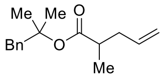

a colorless oil (202.5 mg, 82% yield) for 1.0 mmol scale. The product was found to be difficult to obtain high purity. A second purification was performed to afford pure product (117.8 mg)

R_f = 0.52 (90 / 10 petroleum ether / ethyl acetate)

^{1}H NMR (300 MHz, Chloroform-*d*): δ 7.38 – 7.16 (m, 5H), 5.87 – 5.64 (m, 1H), 5.11 – 4.97 (m, 2H), 3.08 (t, *J* = 2.9 Hz, 3H), 2.50 – 2.30 (m, 2H), 2.30 – 2.05 (m, 1H), 1.47 (d, *J* = 2.8 Hz, 6H), 1.16 – 1.05 (m, 2H).

^{13}C NMR ^{13}C NMR (75 MHz, $CDCl_3$): δ 175.51, 137.20, 135.66, 130.67, 127.83, 126.40, 116.59, 81.81, 46.81, 40.17, 37.88, 25.88, 16.63.

IR (thin film, NaCl): ν_{max} = 3064, 3030, 2976, 2934, 1728, 1642, 1495, 1455, 1383, 1368, 1279, 1256, 1188, 1172, 1120, 1031, 993, 916 cm^{-1}.

HRMS: $C_{16}H_{22}O_2$ $[M-H]^-$ calc: 245.1541, found: 245.1536.

3-(2-ethyl-2-methylpent-4-enoyl)oxazolidin-2-one (6g)

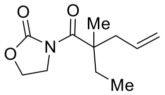

a colorless oil (63% yield by NMR with mesitylene as an internal standard) for 0.50 mmol scale.

R_f = 0.58 (80 / 20 petroleum ether / EtOAc)

^{1}H NMR (400 MHz, Chloroform-*d*): δ 5.71 (ddt, *J* = 16.8, 10.1, 7.4 Hz, 1H), 5.10 – 5.00 (m, 2H), 4.38 (td, *J* = 8.0, 0.6 Hz, 2H), 4.07 – 3.98 (m, 2H), 2.87 (ddd, *J* = 14.1, 7.6, 1.2 Hz, 1H), 2.36 (ddt, *J* = 14.1, 7.2, 1.3 Hz, 1H), 2.19 – 2.07 (m, 1H), 1.77 – 1.66 (m, 1H), 1.29 (s, 3H), 0.86 – 0.80 (m, 3H).

^{13}C NMR (100 MHz, $CDCl_3$): δ 177.16, 152.33, 134.38, 117.75, 62.16, 49.35, 45.28, 40.40, 28.61, 21.75, 9.02.

IR (thin film, NaCl): ν_{max} = 3077, 2969, 2923, 2880, 1779, 1684, 1477, 1383, 1254, 1204, 1109, 1045, 999, 942, 918 cm^{-1}.

HRMS: $C_{11}H_{17}NO_3$ $[M+H^+]$, calc: 212.1287, found: 212.1283.

(*4S*)- (E)-3-(2-ethyl-2-methyl-5-phenylpent-4-enoyl)oxazolidin-2-one (6h)

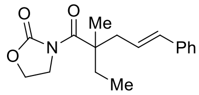

a colorless oil (111.9 mg, 78% yield) for 0.50 mmol scale.

$\mathbf{R_f}$ = 0.52 (90 / 10 petroleum ether / ethyl acetate)

^{1}H NMR (400 MHz, $CDCl_3$): δ 7.31 (dd, *J* = 11.8, 7.0 Hz, 4H), 7.21 (d, *J* = 6.9 Hz, 1H), 6.43 (d, *J* = 15.8 Hz, 1H), 6.18 – 6.07 (m, 1H), 4.39 – 4.32 (m, 2H), 4.06 – 3.98 (m, 2H), 3.01 (dd, *J* = 13.9, 7.9 Hz, 1H), 2.54 (dd, *J* = 14.1, 7.2 Hz, 1H), 2.18 (dd, *J* = 14.3, 7.4 Hz, 1H), 1.79 (dd, *J* = 14.2, 7.4 Hz, 1H), 1.37 (s, 3H), 0.87 (t, *J* = 7.5 Hz, 3H).

^{13}C NMR (100 MHz, $CDCl_3$): δ 177.42, 137.68, 133.07, 128.72, 127.38, 126.36, 126.30, 62.48, 50.05, 45.56, 39.97, 29.03, 22.18, 9.35.

IR (thin film, NaCl): ν_{max} = 3078, 2930, 2880, 1781, 1690, 1480, 1383, 1226, 1110, 948 cm^{-1}.

HRMS: $C_{17}H_{22}NO_3$ [M+H$^+$], calc: 288.1600, found: 288.1610.

(*4S*)-4-benzyl-3-(2-ethyl-2-methylpent-4-enoyl)oxazolidin-2-one (8a)

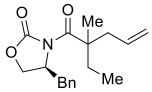

a clear, colorless oil (114.4 mg, 76% yield, 77.5:22.5 d.r.) for 0.50 mmol scale.

$\mathbf{R_f}$ = 0.61 (90 / 10 petroleum ether / ethyl acetate)

^{1}H NMR (300 MHz, Chloroform-*d*): δ 7.41 – 7.17 (m, 5H), 5.80 (m, 1H), 5.21 – 5.01 (m, 2H), 4.71 (ddt, *J* = 10.0, 6.5, 3.4 Hz, 1H), 4.22 – 4.05 (m, 2H), 3.32 (dd, *J* = 13.1, 3.3 Hz, 1H), 3.04 (dd, *J* = 14.0, 7.6 Hz, 1H), 2.65 (dd, *J* = 13.1, 10.4 Hz, 1H), 2.40 (ddt, *J* = 14.0, 7.2, 1.2 Hz, 1H), 2.16 (dq, *J* = 14.8, 7.5 Hz, 1H), 1.76 (dq, *J* = 14.1, 7.6 Hz, 1H), 1.36 (s, 3H), 0.89 (t, *J* = 7.4 Hz, 3H).

^{13}C NMR (100 MHz, $CDCl_3$): δ 176.74, 152.27, 152.21, 135.71, 135.64, 134.33, 134.25, 129.31, 128.83, 127.14, 117.95, 117.71, 66.08, 57.86, 49.47, 49.36, 40.49, 40.38, 38.01, 28.85, 28.58, 21.98, 21.88, 9.03, 8.96.

IR (thin film, NaCl): ν_{max} = 3540, 3344, 3065, 3028, 2971, 2933, 2880, 1780, 1685, 1639, 1604, 1455, 1382, 1348, 1289, 1225, 1108, 1048, 1018, 996, 919 cm^{-1}.

HRMS: $C_{17}H_{21}NO_3$ [M+H$^+$] calc: 302.1756, found: 302.1749.

(4*R*)-3-(2-ethyl-2-methylpent-4-enoyl)-4-phenyloxazolidin-2-one (8b)

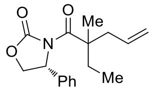

a clear, colorless oil (17.5 mg, 71% yield, 94:6 d.r.) for 0.10 mmol scale.

$\mathbf{R_f}$ = 0.45 (70 / 30 petroleum ether / ethyl acetate)

^{1}H NMR (400 MHz, Chloroform-*d*): δ 7.41 – 7.27 (m, 5H), 5.62 – 5.49 (m, 1H), 5.46 (dd, *J* = 8.6, 4.6 Hz, 1H), 5.02 (m, 1H), 4.98 – 4.90 (m, 1H), 4.69 – 4.60 (m, 1H), 4.27 – 4.17 (m, 1H), 2.80 (ddt, *J* = 14.1, 7.3, 1.3 Hz, 1H), 2.43 (ddt, *J* = 14.2, 7.7, 1.3 Hz, 1H), 2.06 (dt, *J* = 14.7, 7.5 Hz, 1H), 1.71 – 1.57 (m, 1H), 1.26 (s, 3H), 0.68 (td, *J* = 7.5, 0.6 Hz, 3H).

^{13}C NMR (100 MHz, $CDCl_3$): δ 176.69, 152.64, 139.07, 133.98, 128.87, 128.48, 126.10, 117.78, 69.76, 59.94, 49.12, 40.56, 28.86, 21.22, 8.73.

IR (thin film, NaCl): ν_{max} = 3540, 3074, 3035, 2974, 2938, 2881, 1780, 1690, 1639, 1495, 1458, 1382, 1318, 1224, 1106, 1083, 1054, 1002, 944, 919 cm^{-1}.

HRMS: $C_{17}H_{21}NO_3$ [H^+] calc: 288.1594 found 288.1600.

(4*R*)-3-((E)-2-ethyl-2-methyl-5-phenylpent-4-enoyl)-4-phenyloxazolidin-2-one (8c)

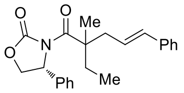

a clear, colorless oil (210.0 mg, 59% yield, 91:9 d.r.) 1.0 mmol scale.

$\mathbf{R_f}$ = 0.37 (90 / 10 petroleum ether / ethyl acetate)

^{1}H NMR (400 MHz, Chloroform-*d*): δ 7.33 – 7.17 (m, 10H), 6.43 (d, *J* = 15.6 Hz, 1H), 5.94 (dt, *J* = 15.3, 7.5 Hz, 1H), 5.48 (dd, *J* = 8.6, 4.6 Hz, 1H), 4.66 (t, *J* = 8.7 Hz, 1H), 4.23 (dd, *J* = 8.8, 4.7 Hz, 1H), 3.13 (dd, *J* = 14.3, 7.5 Hz, 1H), 2.51 (ddd, *J* = 14.2, 7.4, 1.4 Hz, 1H), 2.20 (dq, *J* = 14.8, 7.5 Hz, 1H), 1.70 (dt, *J* = 14.1, 7.4 Hz, 1H), 1.32 (s, 3H), 0.77 (t, *J* = 7.4 Hz, 3H).

^{13}C NMR (100 MHz, $CDCl_3$): δ 176.63, 152.73, 139.05, 137.27, 132.79, 128.99, 128.41, 128.34, 127.02, 126.10, 126.06, 125.85, 69.86, 60.04, 49.78, 39.98, 29.05, 21.91, 8.89.

IR (thin film, NaCl): ν_{max} = 3030, 2967, 2919, 2879, 1779, 1689, 1494, 1457, 1382, 1317, 1198, 1103, 1082, 1047, 970 cm^{-1}.

HRMS: $C_{23}H_{25}NO_3$ [M+H^+] calc: 364.1913, found: 364.1903.

(4*R*)-3-(2-methyl-2-propylpent-4-enoyl)-4-phenyloxazolidin-2-one (8d)

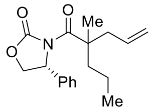

a white solid (97.8 mg, 65% yield, >95:5 d.r.) 0.50 mmol scale.

$\mathbf{R_f}$ = 0.48 (90 / 10 petroleum ether / ethyl acetate)

^{1}H NMR (500 MHz, Chloroform-*d*): δ 7.42 – 7.28 (m, 5H), 5.55 (ddt, *J* = 17.3, 10.1, 7.3 Hz, 1H), 5.47 (dd, *J* = 8.6, 4.5 Hz, 1H), 5.02 (dq, *J* = 17.0, 1.7 Hz, 1H), 4.97 – 4.91 (m, 1H), 4.67 (t, *J* = 8.7 Hz, 1H), 4.25 (dd, *J* = 8.8, 4.5 Hz, 1H), 2.85 – 2.77 (m, 1H), 2.42 (ddd, *J* = 14.1, 7.5, 1.3 Hz, 1H), 2.00 (ddd, *J* = 13.7, 12.3, 4.6 Hz, 1H), 1.57 (ddd, *J* = 13.7, 12.3, 5.9 Hz, 1H), 1.27 (s, 3H), 1.14 (ttd, *J* = 14.5, 7.2, 4.6 Hz, 1H), 1.05 – 0.92 (m, 1H), 0.84 (t, *J* = 7.2 Hz, 3H).

^{13}C NMR (126 MHz, $CDCl_3$): δ 176.84, 152.69, 139.14, 134.05, 128.96, 128.58, 126.18, 117.88, 69.82, 60.00, 48.92, 40.98, 38.50, 22.01, 17.72, 14.54.

IR (thin film, NaCl): ν_{max} = 3068, 2960, 2929, 2873, 1781, 1690, 1639, 1457, 1381, 1316, 1222, 1107, 1082, 1048, 996, 917 cm^{-1}.

HRMS: $C_{18}H_{23}NO_3$ [M+H^+] calc: 302.1751, found: 302.1746.

(4*R*)-3-(2-allyl-2,5-dimethylhexanoyl)-4-phenyloxazolidin-2-one (8e)

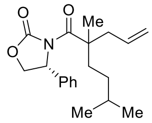

a colorless oil (33.3 mg, 51% yield, 90:10 d.r.) 0.20 mmol scale.

R_f = 0.59 (90 / 10 petroleum ether / ethyl acetate)

^{1}H NMR (400 MHz, Chloroform-*d*): δ 7.40 – 7.29 (m, 5H), 5.57 (ddt, *J* = 17.3, 10.1, 7.4 Hz, 1H), 5.47 (dd, *J* = 8.5, 4.4 Hz, 1H), 5.07 – 5.00 (m, 1H), 4.95 (ddt, *J* = 10.0, 2.2, 1.0 Hz, 1H), 4.66 (t, *J* = 8.7 Hz, 1H), 4.29 – 4.23 (m, 1H), 2.80 (ddt, *J* = 14.1, 7.2, 1.3 Hz, 1H), 2.40 (ddt, *J* = 14.0, 7.5, 1.2 Hz, 1H), 1.98 (ddd, *J* = 13.7, 12.7, 4.5 Hz, 1H), 1.65 (ddd, *J* = 13.7, 12.7, 4.5 Hz, 1H), 1.48 – 1.35 (m, 1H), 1.27 (s, 6H), 0.96 (tdd, *J* = 12.8, 6.5, 4.5 Hz, 1H), 0.84 – 0.75 (m, 6H).

^{13}C NMR (101 MHz, $CDCl_3$): δ 176.95, 152.66, 139.12, 134.07, 128.97, 128.61, 126.28, 117.92, 69.82, 60.02, 48.82, 41.08, 34.01, 33.52, 29.69, 28.40, 22.55, 22.49, 21.89.

IR (thin film, NaCl): ν_{max} = 3081, 3039, 2954, 2926, 2867, 1775, 1694, 1469, 1458, 1386, 1355, 1330, 1255, 1234, 1207, 1115, 1087, 1061, 1038, 999, 919 cm^{-1}.

HRMS: $C_{20}H_{27}NO_3$ [M+Na^+] calc: 352.1883, found: 352.1881.

(*4R*)-3-(2-(2-(benzyloxy)ethyl)-2-methylpent-4-enoyl)-4-phenyloxazolidin-2-one (8f)

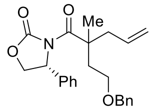

a white solid (52.0 mg, 33% yield, >95:5 d.r.) 0.40 mmol scale.

R_f = 0.6 (80 / 20 petroleum ether / ethyl acetate)

^{1}H NMR (400 MHz, Chloroform-*d*): δ 7.40 – 7.26 (m, 8H), 7.17 – 7.15 (m, 2H), 5.50 (dd, *J* = 17.0, 10.1 Hz, 1H), 5.56 – 5.45 (m, 1H), 5.05 – 5.01 (m, 1H), 4.93-4.90 (m, 1H), 4.81 (dd, *J* = 8.7, 4.1 Hz, 1H), 4.38 (d, *J* = 10.7 Hz, 1H), 4.28 (d, *J* = 10.7 Hz, 1H), 3.86 (dd, *J* = 8.6, 4.1 Hz, 1H), 3.78 (t, *J* = 8.6 Hz, 1H), 3.57 – 3.53 (m, 2H), 3.12 – 3.04 (m, 2H), 2.37 (dd, *J* = 14.0, 7.4 Hz, 1H), 1.57 – 1.50 (m, 1H), 1.23 (s, 3H).

^{13}C NMR (100 MHz, $CDCl_3$): δ 175.95, 153.17, 139.98, 138.44, 133.52, 128.80, 128.42, 128.31, 128.17, 127.80, 125.95, 118.20, 73.47, 69.52, 67.10, 59.90, 47.08, 41.39, 36.04, 23.02.

IR (thin film, NaCl): ν_{max} = 2986, 2896, 1780, 1689, 1452, 1377, 1325, 1304, 1227, 1201, 1178, 1151, 1106, 1045 cm^{-1}

HRMS: $C_{24}H_{27}NO_4$ [M+H^+] calc: 394.2013, found: 394.2011.

(*4R*)-3-(2-allyl-2-methylhexanoyl)-4-phenyloxazolidin-2-one (8g)

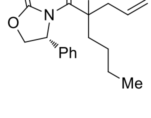

a white solid (99.2 mg, 63% yield, >95:5 d.r.) 0.50 mmol scale.

R_f = 0.19 (90 / 10 petroleum ether / ethyl acetate)

^{1}H NMR (400 MHz, Chloroform-*d*): δ 7.41 – 7.28 (m, 5H), 5.56 (ddt, *J* = 17.3, 10.0, 7.3 Hz, 1H), 5.47 (dd, *J* = 8.6, 4.5 Hz, 1H), 5.07 – 4.99 (m, 1H), 4.95 (ddt, *J* = 10.0, 2.2, 1.0 Hz, 1H), 4.67 (t, *J* = 8.7 Hz, 1H),

4.25 (dd, *J* = 8.8, 4.5 Hz, 1H), 2.80 (ddt, *J* = 14.1, 7.2, 1.2 Hz, 1H), 2.41 (ddt, *J* = 14.0, 7.5, 1.2 Hz, 1H), 1.99 (ddd, *J* = 13.7, 12.3, 4.6 Hz, 1H), 1.62 (ddd, *J* = 13.7, 12.4, 4.4 Hz, 1H), 1.27 (s, 3H), 1.25 – 1.17 (m, 2H), 1.07 (tddd, *J* = 12.5, 8.0, 6.4, 4.5 Hz, 1H), 1.00 – 0.85 (m, 1H), 0.82 (t, *J* = 7.3 Hz, 3H).

^{13}C NMR (100 MHz, $CDCl_3$): δ 176.93, 152.68, 139.10, 134.06, 128.95, 128.59, 126.23, 117.88, 69.81, 60.00, 48.83, 41.02, 35.95, 26.69, 23.09, 21.92, 13.97.

IR (thin film, NaCl): ν_{max} = 3074, 3035, 2958, 2929, 2871, 1781, 1690, 1639, 1495, 1457, 1381, 1316, 1211, 1106, 1082, 1049, 997, 923 cm-1.

HRMS: $C_{19}H_{25}NO_3$ [M+H$^+$] calc: 316.1907, found 316.1902.

***tert*-butyl benzyl(4-methyl-4-((*R*)-2-oxo-4-phenyloxazolidine-3-carbonyl)hept-6-en-1-yl)carbamate (8h)**

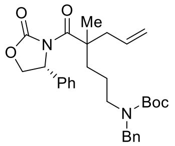

a colorless oil (52.1 mg, 52% yield, 91:9 d.r.) 0.20 mmol scale.

R_f = 0.45 (80 / 20 petroleum ether / ethyl acetate)

^{1}H NMR (400 MHz, Benzene-*d*$_6$): δ 7.11 (q, *J* = 7.3 Hz, 5H), 7.06 – 6.94 (m, 5H), 5.58 (ddt, *J* = 17.3, 10.1, 7.3 Hz, 1H), 4.99 (dq, *J* = 17.0, 1.4 Hz, 1H), 4.96 – 4.91 (m, 1H), 4.91 – 4.85 (m, 1H), 4.35 (d, *J* = 3.3 Hz, 2H), 4.28 (s, 2H), 3.83 – 3.70 (m, 1H), 3.51 (ddd, *J* = 8.6, 4.6, 1.7 Hz, 1H), 3.15 – 2.96 (m, 2H), 2.86 – 2.75 (m, 1H), 2.53 (dd, *J* = 14.1, 7.6 Hz, 1H), 2.05 (td, *J* = 13.5, 12.1, 3.9 Hz, 1H), 1.42 (s, 9H), 1.26 (s, 3H).

^{13}C NMR (100 MHz, Benzene-*d*$_6$): δ 176.70, 153.26, 140.27, 139.84, 134.92, 129.38, 129.05, 128.91, 127.63, 127.07, 118.35, 79.56, 69.87, 60.43, 53.67, 49.37, 41.94, 36.26, 34.51, 28.93, 24.13, 22.32.

IR (thin film, NaCl): ν_{max} = 3370, 3066, 2927, 2854, 1780, 1691, 1456, 1416, 1382, 1366, 1315, 1244, 1169, 1105, 1045 cm^{-1}.

HRMS: $C_{30}H_{38}N_2O_5$ [M+H$^+$] calc: 507.2854, found: 507.2848.

(*4R*)-3-(2-(3-(benzyloxy)propyl)-2-methylpent-4-enoyl)-4-phenyloxazolidin-2-one (8i)

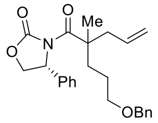

a yellow sticky solid (51.3 mg, 63% yield, >95:5 d.r.) 0.20 mmol scale.

R_f = 0.63 (80 / 20 petroleum ether / ethyl acetate)

^{1}H NMR (400 MHz, Chloroform-*d*): δ 7.37 – 7.26 (m, 10H), 5.57 – 5.52 (m, 1H), 5.43 (dd, *J* = 8.5, 4.4 Hz, 1H), 4.99 (dd, *J* = 34.7, 13.5 Hz, 2H), 4.61 (t, *J* = 8.7 Hz, 1H), 4.45 (s, 2H), 4.21 (dd, *J* = 8.7, 4.5 Hz, 1H), 3.39 (t, *J* = 6.5 Hz, 2H), 2.84 (dd, *J* = 14.1, 7.3 Hz, 1H), 2.46 (dd, *J* = 14.0, 7.5 Hz, 1H), 2.14 – 2.11 (m, 1H), 1.49 (dd, *J* = 11.8, 6.0 Hz, 2H), 1.28 (s, 3H).

^{13}C NMR (100 MHz, $CDCl_3$): δ 176.61, 152.72, 139.11, 138.46, 133.83, 128.97, 128.57, 128.31, 127.62, 127.48, 126.16, 118.07, 72.78, 70.38, 69.81, 60.04, 48.73, 40.86, 32.70, 29.68, 24.91, 21.91.

IR (thin film, NaCl): ν_{max} = 3370, 3066, 2925, 2854, 1777, 1716, 1603, 1453, 1383, 1316, 1279, 1207, 1113, 1070, 1027, 934 cm^{-1}.

HRMS: $C_{25}H_{29}NO_4$ $[M+H^+]$ calc: 408.2169, found: 408.2167.

(4*R*)-3-(2-methyl-2-(3-phenylpropyl)pent-4-enoyl)-4-phenyloxazolidin-2-one (8j)

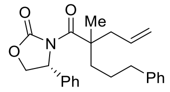

a clear, colorless oil (23.6 mg, 65% yield, 92:8 d.r.) 0.10 mmol scale.

$\mathbf{R_f}$ = 0.56 (70 / 30 petroleum ether / ethyl acetate)

^{1}H NMR (300 MHz, Chloroform-*d*): δ 7.43 – 7.27 (m, 7H), 7.27 – 7.18 (m, 1H), 7.18 – 7.11 (m, 2H), 5.60 (ddt, *J* = 17.2, 10.0, 7.3 Hz, 1H), 5.48 (dd, *J* = 8.6, 4.7 Hz, 1H), 5.08 (ddt, *J* = 17.0, 2.5, 1.4 Hz, 1H), 5.01 (ddt, *J* = 10.0, 2.1, 1.0 Hz, 1H), 4.64 (t, *J* = 8.7 Hz, 1H), 4.25 (dd, *J* = 8.8, 4.7 Hz, 1H), 2.86 (ddt, *J* = 14.1, 7.2, 1.3 Hz, 1H), 2.58 (t, *J* = 7.7 Hz, 2H), 2.49 (ddt, *J* = 14.0, 7.5, 1.2 Hz, 1H), 2.14 (ddd, *J* = 13.7, 12.4, 4.4 Hz, 1H), 1.75 (ddd, *J* = 13.7, 12.5, 4.5 Hz, 1H), 1.51 (ttd, *J* = 12.3, 7.7, 4.5 Hz, 1H), 1.41 – 1.21 (m, 4H).

^{13}C NMR (75 MHz, $CDCl_3$): δ 176.65, 152.57, 142.07, 138.91, 133.82, 128.87, 128.50, 128.22, 128.11, 126.13, 125.62, 117.93, 69.70, 59.92, 48.73, 40.92, 36.05, 35.96, 26.41, 21.70.

IR (thin film, NaCl): ν_{max} = 3063, 3028, 2975, 2933, 2864, 1952, 1780, 1689, 1639, 1603, 1495, 1455, 1382, 1318, 1195, 1104, 1082, 1045, 1001, 921 cm^{-1}.

HRMS: $C_{24}H_{27}NO_3$ $[H^+]$ calc: 378.2064, found: 378.2067

D. Synthesis of Electrophiles

2-(Dimethylamino)ethyl 1H-imidazole-1-carboxylate (S1)

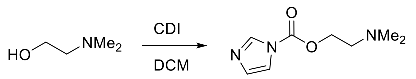

To a stirring suspension of 1,1'-carbonyldiimidazole (13.68g, 90 mmol, 2.0 eq) in 100 mL dichloromethane was added *N*,*N*-dimethylaminoethanol (5.0mL, 4.44g, 49 mmol, 1.0 eq, d-0.886) dropwise. The reaction turned clear upon completion of the addition of *N*,*N*-dimethylaminoethanol. After 30 minutes, the solution was quickly washed twice with 20 mL water. The organic layer was dried over sodium sulfate and evaporated to a clear oil (8.62g, 96% yield) of purity to obviate further purification. The reaction was similarly performed on a 100 mmol scale with two 40 mL washes to afford the product in lower yield (15.37g, 84% yield). It was observed that highest yields were obtained by rapid washing of the product followed by drying. Due to the sensitivity of the product, it was used in the next step without further purification.

This molecule has been previously synthesized by a similar procedure.[6]

^{1}H NMR (400 MHz, Chloroform-*d*): δ 8.15 (s, 1H), 7.43 (m, 1H), 7.07 (m, 1H), 4.50 (t, 2H), 2.71 (t, 2H), 2.32 (s, 6H).

[6] Funhoff, A.M.; Van Nostrum, C.F.; Janssen, A.P.C.A.; Fens, M.H.A.M.; Crommelin, D.J.A.; Hennink, W.E. *Pharmaceutical Research* **2004**, *21*, 170-176.

^{13}C NMR (100 MHz, $CDCl_3$): δ 148.67, 137.08, 130.57, 117.10, 65.89, 57.45, 45.70.

Allyl (2-(dimethylamino)ethyl) carbonate (2b)

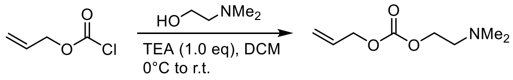

To an ice-cooled, stirring solution of trimethylamine (13.9 mL, 10.1 g, 100 mmol, 1.0 eq, *d* = 0.7255) and *N*,*N*-dimethylethanolamine (10.0 mL, 8.91 g, 100 mmol, 1.0 eq, *d*=0.890) in DCM was added allyl chloroformate (10.6 mL, 12.1g, 100 mmol, 1.0 eq, *d* = 1.134) at a constant rate over 5 min. The solution was triturated with 100 mL pentane and filtered of the ammonium by-product. After removal of solvent, the crude material was purified by FSGC (pure DCM) to afford the product as a light yellow oil (10.6721 g, 62% yield).

R_f = 0.20 (70 / 30 petroleum ether / ethyl acetate)

^{1}H NMR (300 MHz, Chloroform-*d*): δ 5.37 – 5.26 (m, 1H), 5.26 – 5.16 (m, 1H), 4.58 (dd, *J* = 5.8, 1.4 Hz, 2H), 4.19 (t, *J* = 5.8, 2H), 2.55 (t, *J* = 5.8, 2H), 2.24 (s, 6H).

^{13}C NMR (75 MHz, $CDCl_3$): δ 154.91, 131.45, 118.67, 68.28, 65.50, 57.50, 45.59.

IR (thin film, NaCl): ν_{max} = 3086, 2949, 2823, 2772, 1748, 1650, 1457, 1398, 1377, 1338, 1254, 1159, 1099, 1042, 967 cm^{-1}.

HRMS: $C_8H_{15}NO_3$ [$M+H^+$] calc: 174.1130, found: 174.1122.

(*E*)-2-(dimethylamino)ethyl (3-(4-methoxyphenyl)allyl) carbonate (5d)

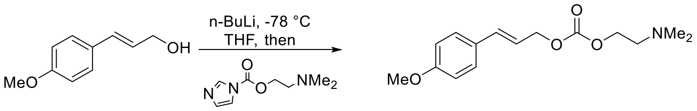

To a stirring solution of 4-methoxy cinnamyl alcohol (0.492g, 3.0 mmol, 1.0 eq) in THF at -78^0 C was added a solution of n-butyllithium (1.20 mL, 3.0 mmol, 1.0 eq, 2.5 M in hexanes) dropwise. The solution was allowed to stir for 20 minutes, and acyl imidazole (1.099g, 6.0 mmol, 2.0 eq) was added dropwise. The solution was allowed to stir at -78^0 C until precipitate was observed. The reaction was warmed to r.t., and 40 mL of water was added, followed by 40 mL of diethyl ether. The organic layer was separated, and the aqueous phase was extracted with 40 mL diethyl ether. The solvent was dried over anhydrous magnesium sulfate, filtered, and evaporated to an oil. The product was purified by flash silica gel chromatography (pure DCM to 95% DCM / 5% triethylamine) to afford a clear liquid (0.3404 g, 41% yield).

R_f = 0.55 (50 / 50 petroleum ether / ethyl acetate)

^{1}H NMR (400 MHz, Chloroform-*d*): δ 7.32 – 7.25 (m, 2H), 6.84 – 6.78 (m, 2H), 6.65 – 6.53 (m, 1H), 6.12 (dt, *J* = 15.8, 6.6 Hz, 1H), 4.72 (dd, *J* = 6.6, 1.3 Hz, 2H), 4.24 – 4.15 (t, J=5.8Hz, 2H), 3.76 (s, 3H), 2.56 (t, *J* = 5.8 Hz, 2H), 2.25 (s, 6H).

^{13}C NMR (100 MHz, $CDCl_3$): δ 159.45, 154.94, 134.34, 128.59, 127.75, 119.92, 113.80, 68.43, 65.43, 57.46, 55.05, 45.55.

IR (thin film, NaCl): ν_{max} = 3367, 2933, 2838, 1654, 1606, 1512, 1463, 1249, 1176, 1088, 1030, 971 cm^{-1}.

HRMS: $C_{15}H_{21}NO_4$ [M^+] calc: 279.14706, found: 279.14700.

(*E*)-2-(dimethylamino)ethyl (3-(4-nitrophenyl)allyl) carbonate (5e)

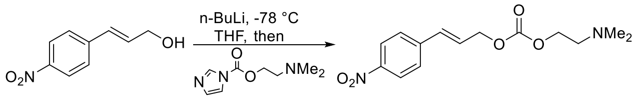

The product was obtained by the previously described procedure, using 4-nitrocinnamyl alcohol (0.5372 g, 3.0 mmol, 1.0 eq). An orange oil was obtained (0.2563 g, 29%). The product was found to be approximately 90% pure by NMR and was used in alkylation reactions without further purification.

R_f = 0.09 (50 / 50 petroleum ether / ethyl acetate)

^{1}H NMR (400 MHz, Chloroform-*d*): δ 8.16 – 8.11 (m, 2H), 7.49 – 7.45 (m, 2H), 6.71 (dt, *J* = 16.0, 1.4 Hz, 1H), 6.42 (dt, *J* = 16.0, 5.9 Hz, 1H), 4.79 (dd, *J* = 5.9, 1.4 Hz, 2H), 4.22 (t, *J* = 5.7 Hz, 2H), 2.61 – 2.51 (t, *J* = 5.7 Hz, 2H), 2.25 (s, 6H).

^{13}C NMR (100 MHz, $CDCl_3$): δ 154.82, 147.08, 142.33, 131.35, 127.36, 127.04, 123.86, 67.26, 65.69, 57.49, 45.57.

IR (thin film, NaCl): ν_{max} = 2953, 2864, 2923, 2773, 1747, 1598, 1519, 1457, 1344, 1261, 1109, 1040, 959 cm^{-1}.

HRMS: $C_{14}H_{18}N_2O_5$ [M+H+] calc: 295.1294, found: 295.1290.

(*E*)-2-(dimethylamino)ethyl (3-(p-tolyl)allyl) carbonate (5f)

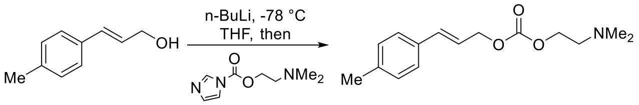

The product was obtained by the previously described procedure, using 4-methylcinnamyl alcohol (0.4446 g, 3.0 mmol, 1.0 eq). A light yellow oil was obtained (0.1786 g, 23%).

^{1}H NMR (400 MHz, Chloroform-*d*): δ 7.27 (d, *J* = 8.0 Hz, 2H), 7.12 (d, *J* = 8.0 Hz, 2H), 6.64 (d, *J* = 15.8 Hz, 1H), 6.23 (m, 1H), 4.76 (d, *J* = 6.6 Hz, 2H), 4.24 (t, *J* = 5.8 Hz, 2H), 2.60 (t, *J* = 5.8 Hz, 2H), 2.33 (s, 3H), 2.28 (s, 6H).

^{13}C NMR (100 MHz, $CDCl_3$): δ 155.03, 137.98, 134.67, 133.17, 129.21, 126.50, 121.25, 68.42, 65.55, 57.55, 45.65, 21.16.

IR (thin film, NaCl): ν_{max} = 2947, 2822, 2771, 1747, 1514, 1456, 1398, 1382, 1338, 1261, 1101, 1041, 967 cm^{-1}.

HRMS: $C_{16}H_{21}NO_4$ [$M+H^+$] calc: 292.1549, found: 292.1544.

Cinnamyl (2-(dimethylamino)ethyl) carbonate (5g)

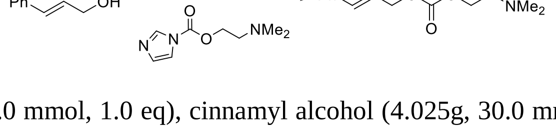

Acyl imidazole (5.496g, 30.0 mmol, 1.0 eq), cinnamyl alcohol (4.025g, 30.0 mmol, 1.0 eq), and potassium hydroxide (0.301g, 6.00 mmol, 0.2 eq) were dissolved in toluene. Temperature ramping revealed no product at 60 ^{0}C, so the reaction was heated further to 90 ^{0}C, where the reaction was observed to proceed to full completion within 10 minutes. The reaction mixture was allowed to reach room temperature, and 50

mL of diethyl ether was added. The solution was washed with saturated sodium bicarbonate (50 mL) and water (50 mL). The solution was dried over sodium sulfate and evaporated to a crude oil. Purification by FSGC (pure diethyl ether) slowly eluted the product as a light yellow oil (4.92g, 66% yield).

R_f = 0.64 (50 / 50 petroleum ether / ethyl acetate)

^{1}H NMR (300 MHz, Chloroform-*d*): δ 7.45 – 7.24 (m, 7H), 6.72 (dd, *J* = 15.9, 4.4 Hz, 1H), 6.39 – 6.26 (m, 1H), 4.84 – 4.78 (m, 2H), 4.31 – 4.24 (m, 2H), 2.63 (t, *J* = 5.8 Hz, 2H).

^{13}C NMR (75 MHz, $CDCl_3$): δ 155.05, 135.99, 134.77, 134.61, 128.55, 128.11, 126.63, 122.37, 68.28, 65.62, 57.58, 45.69.

IR (thin film, NaCl): ν_{max} = 3307, 3058, 3027, 2861, 1736, 1655, 1598, 1493, 1450, 1376, 1093, 1070, 1016, 970, 916 cm^{-1}.

HRMS: $C_{14}H_{19}NO_3$ [$M+H^+$] calc: 250.1443, found: 250.1452.

E. Large preparation of 3a:

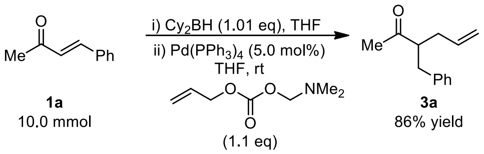

Dicyclohexylborane (1.9 g, 11 mmol) was obtained from the glovebox by addition to an oven-dried microwave vial that was cooled in the vacuum chamber of the glovebox. The solid was suspended in freshly distilled THF (13.0 mL), and neat 4-phenylbut-3-en-2-one acrylate **1a** (1.46 g, 10 mmol) was added dropwise while cooling on an ice bath. After addition, ice was removed from the water/ice mixture, and the water was allowed to warm to r.t. Upon warming to r.t., a clear solution was obtained. The hydroboration was allowed to stir for an additional 30 minutes to ensure complete hydroboration. Solid $Pd(PPh_3)_4$ (577.5 mg, 0.5 mmol) was quickly added by removing the septum with a stream of argon above the solution. After replacement of the septum, a stream of argon was passed over the solution with a needle as an outlet. Allyl *N,N*-dimethyl ethanolamine carbonate **2b** (1.79 g, 11 mmol) was added dropwise. Precipitate was observed within minutes. The reaction was allowed to react for 1 h. to ensure complete conversion. The solution passed through a short silica plug of silica. The solvent was removed under rotary evaporation, and the oil was purified by flash silica gel chromatography to afford colorless oil (1.6 g, 86% yield).

F. Determination of Absolute Stereochemistry

(*R*)-2-ethyl-2-methylpent-4-enoic acid 9

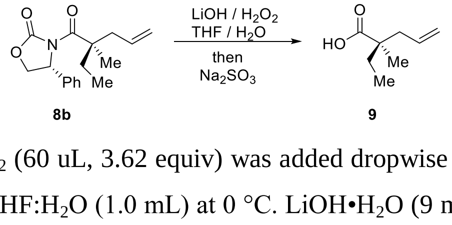

A solution of 30% aqueous H_2O_2 (60 uL, 3.62 equiv) was added dropwise to a solution of substrate **8b** (40 mg, 0.14 mmol, 1.0 eq.) in 4:1 THF:H_2O (1.0 mL) at 0 °C. LiOH•H_2O (9 mg, 1.6 eq.) in H_2O (0.5 mL) was added to the reaction mixture. After 1 h, Na_2SO_3 (70 mg, 4.1 eq.) was added and THF was removed from the slurry under vacuum. The residue was partitioned between DCM and H_2O. The aqueous layers were collected and acidified to pH 1 with 1N HCl. The aqueous layer was extracted with Et_2O, and the combined

organic layers were dried over $MgSO_4$. The solvents were removed in vacuo and the residue was purified by flash column chromatography, eluting with ethyl acetate / hexane mixture (20 : 80) to provide a clear oil **9** (19.0 mg, 96% yield).

R_f = 0.84 (75 : 25 petroleum ether : ethyl acetate)

^{1}H NMR (400 MHz, Chloroform-*d*): δ 5.83 – 5.68 (m, 1H), 5.15 – 5.00 (m, 2H), 2.41 (ddt, *J* = 13.7, 7.1, 1.3 Hz, 1H), 2.21 (ddt, *J* = 13.7, 7.8, 1.1 Hz, 1H), 1.76 – 1.63 (m, 1H), 1.52 (dq, *J* = 13.7, 7.5 Hz, 1H), 1.29 (s, 1H), 1.13 (s, 3H), 0.89 (t, *J* = 7.5 Hz, 3H).

^{13}C NMR (100 MHz, $CDCl_3$): δ 183.39, 133.82, 118.12, 77.00, 46.17, 42.59, 31.36, 20.59, 8.88.

$[\alpha]_D^{23}$ = -7.66^0 (c = 1.00, $CHCl_3$) for 88% *ee* sample.

Literature value for (*R*)-Product[7]:

$[\alpha]_D^{24}$ = -5.64^0 (c = 0.99, $CHCl_3$) for 82% *ee* sample.

[7] Doyle, A. G.; Jacobsen, E. N. *Angew. Chem. Int. Ed*. **2007**, *46*, 3701-3705.

G. NMR spectra:

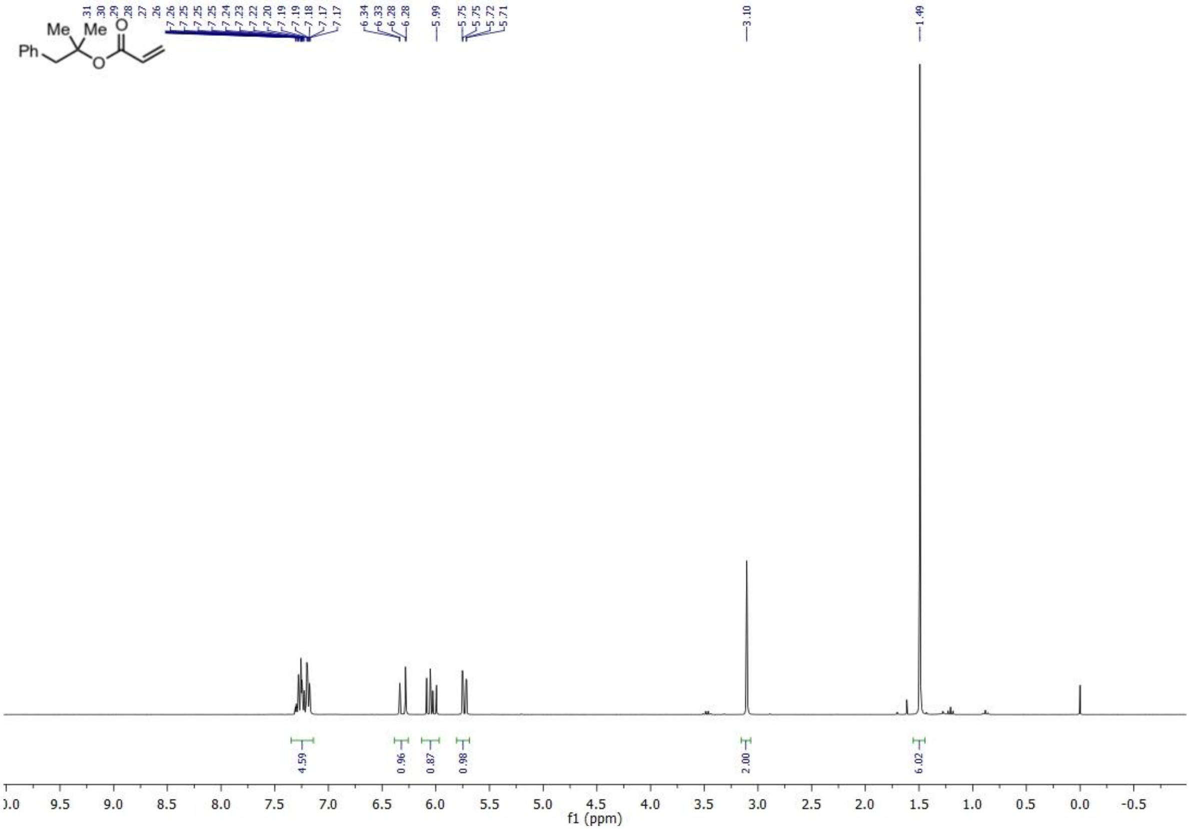

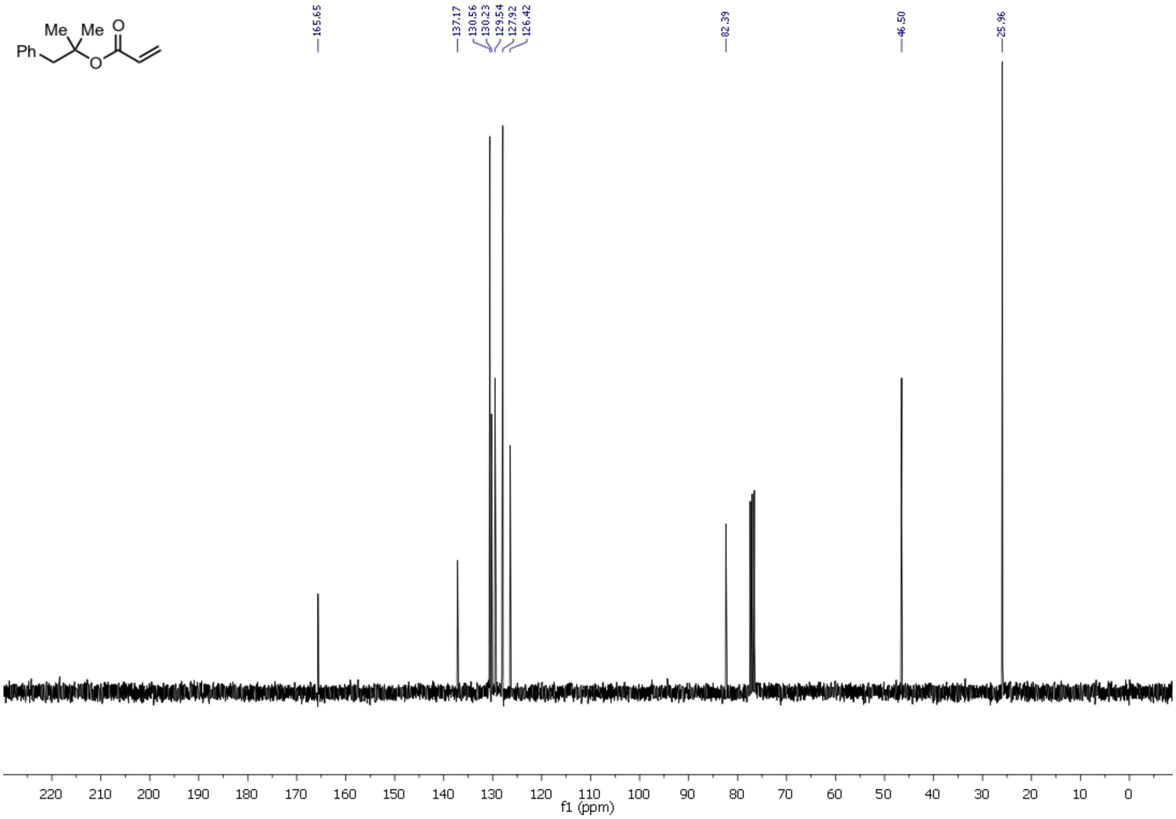

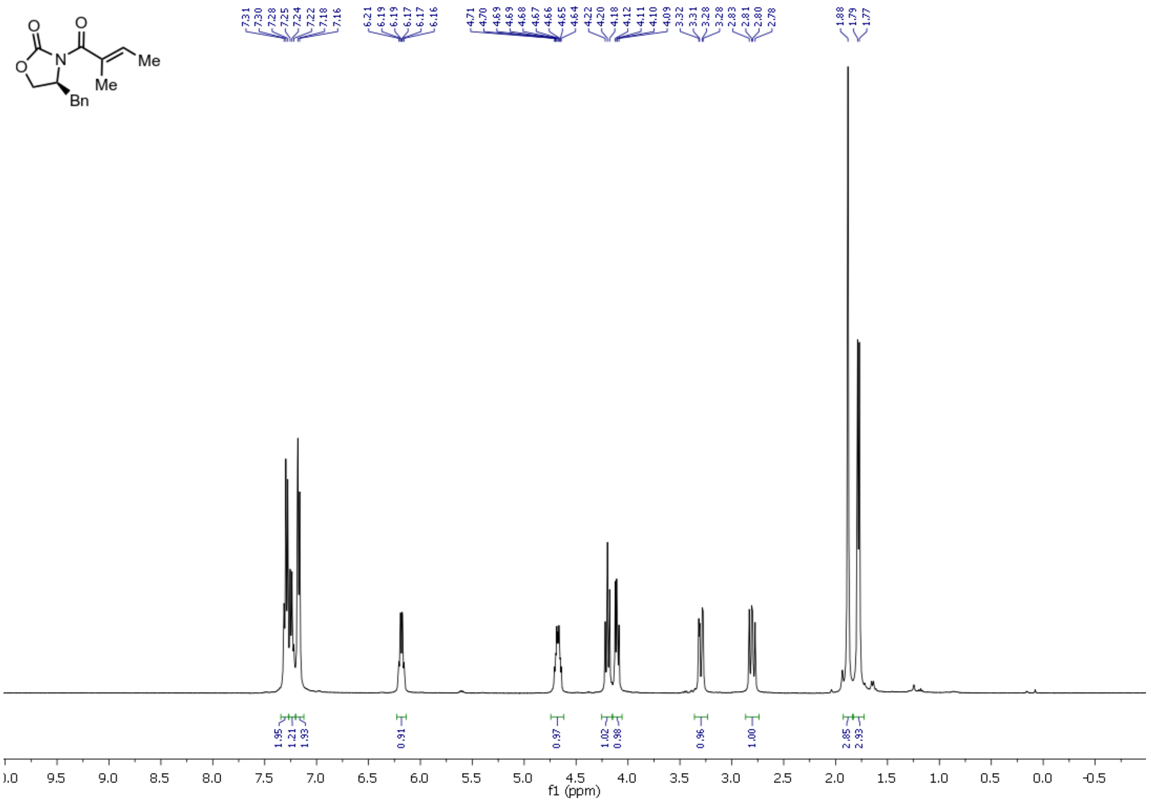
O
N
O
Me
Me
Bn
7.31 7.30 7.28 7.25 7.24 7.22 7.18 7.16
6.21 6.19 6.19 6.17 6.17 6.16
4.71 4.70 4.69 4.69 4.68 4.67 4.66 4.65 4.64
4.22 4.20 4.18 4.12 4.11 4.10 4.09
3.32 3.31 3.28 3.28
2.83 2.81 2.80 2.78
1.88 1.79 1.77
1.95 1.21 1.93
0.91
0.97
1.02 0.98
0.96
1.00
2.85 2.93
f1 (ppm)

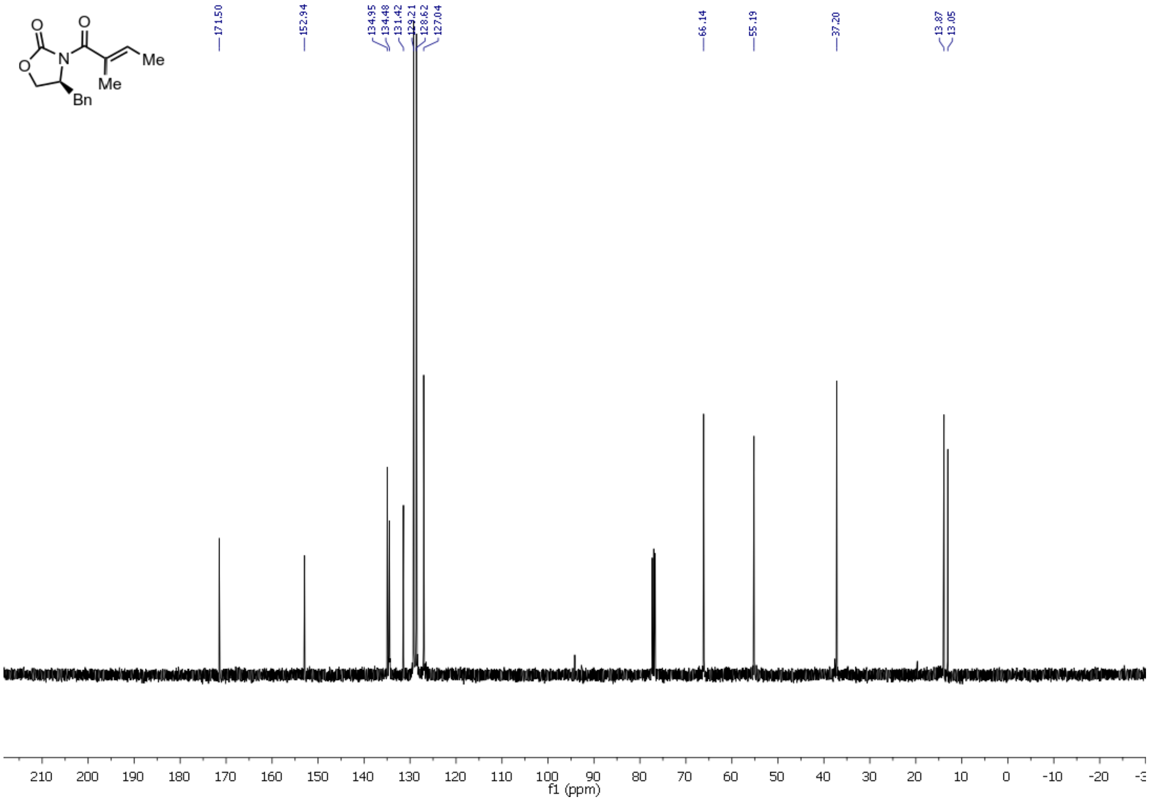
O
N
O
Me
Me
Bn
171.50
152.94
134.95 134.48 131.42 129.21 128.62 127.04
66.14
55.19
37.20
13.87 13.05
f1 (ppm)

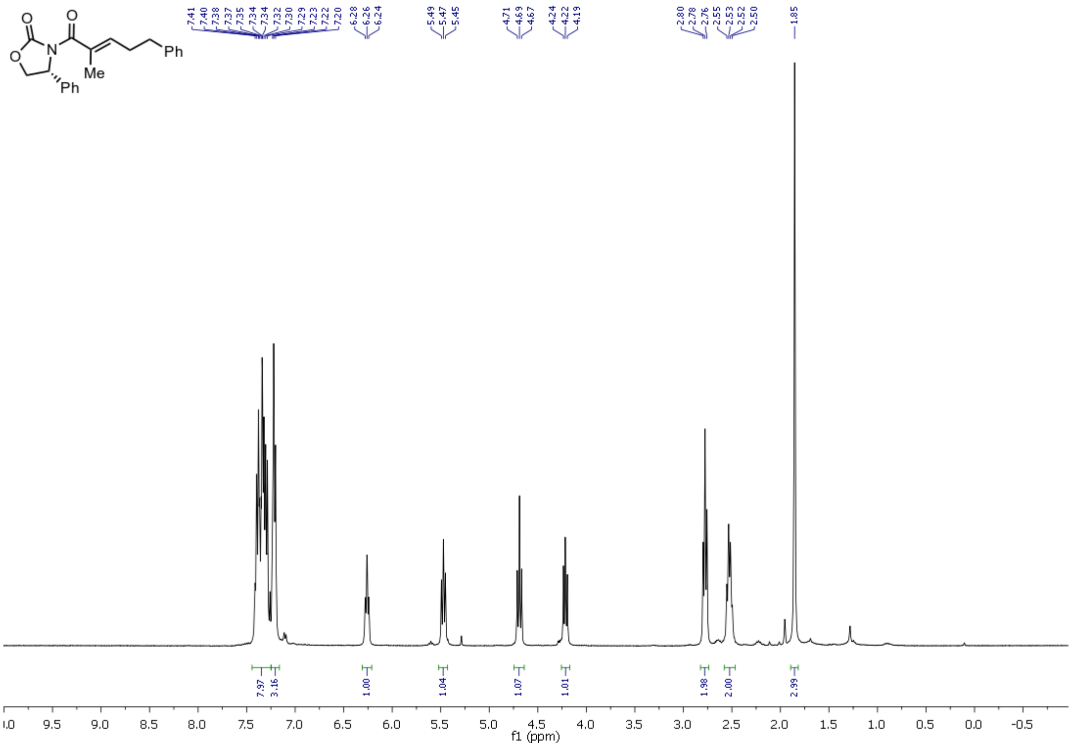

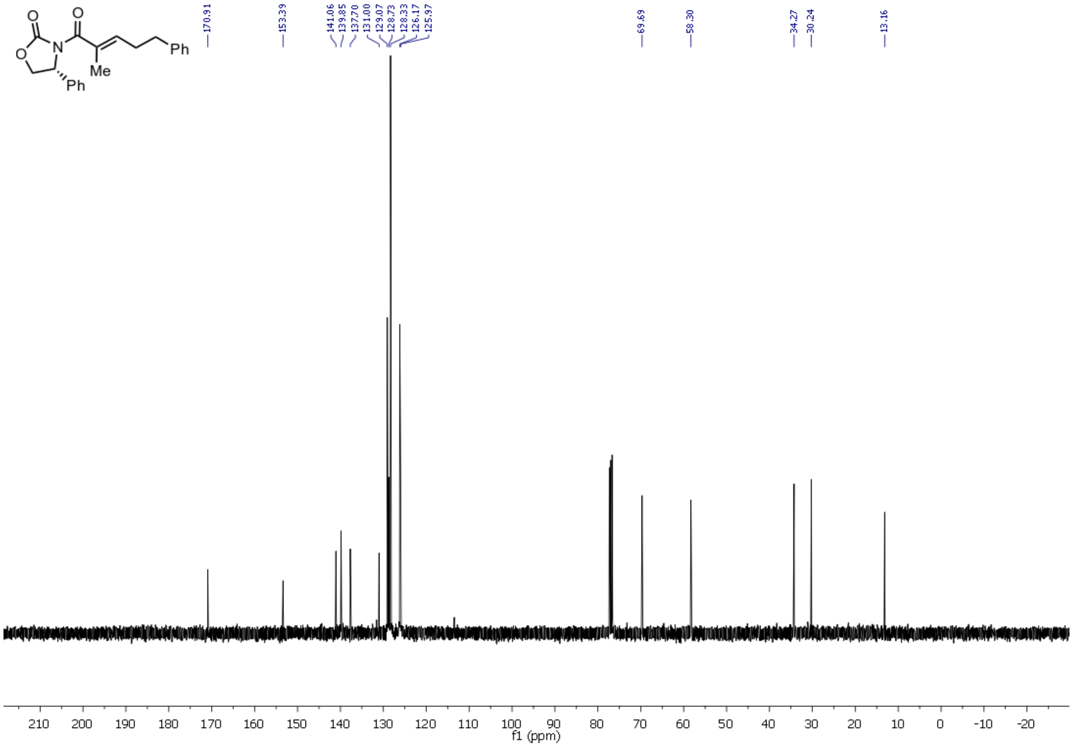

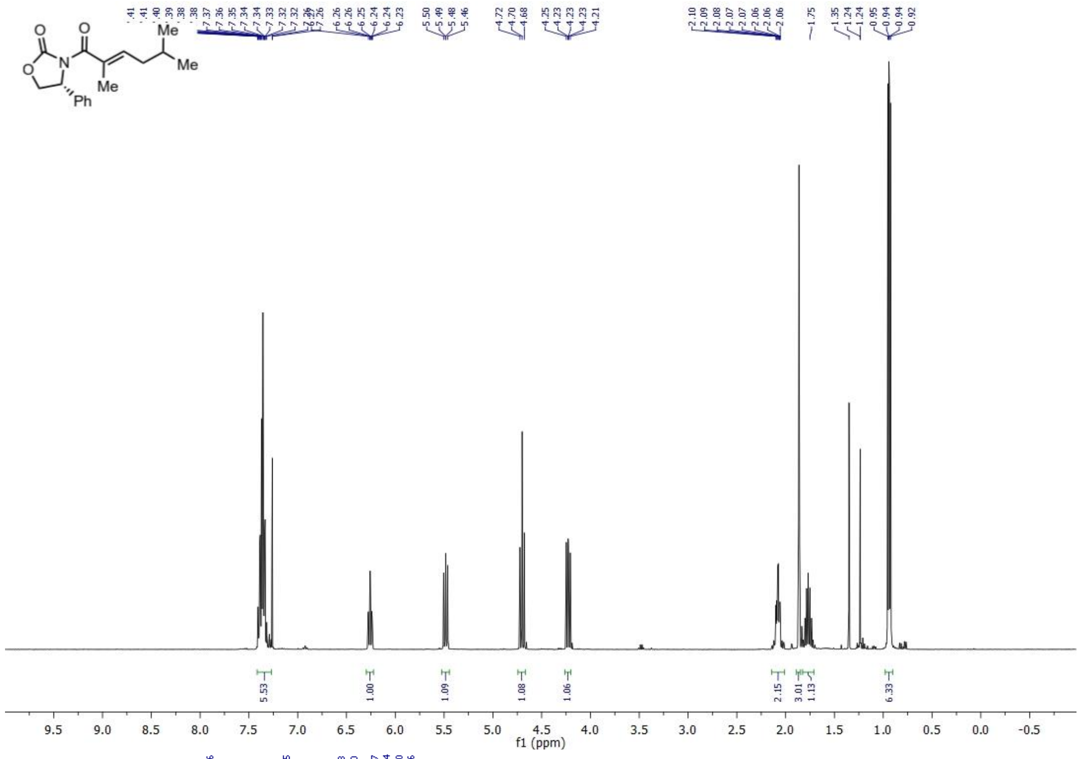

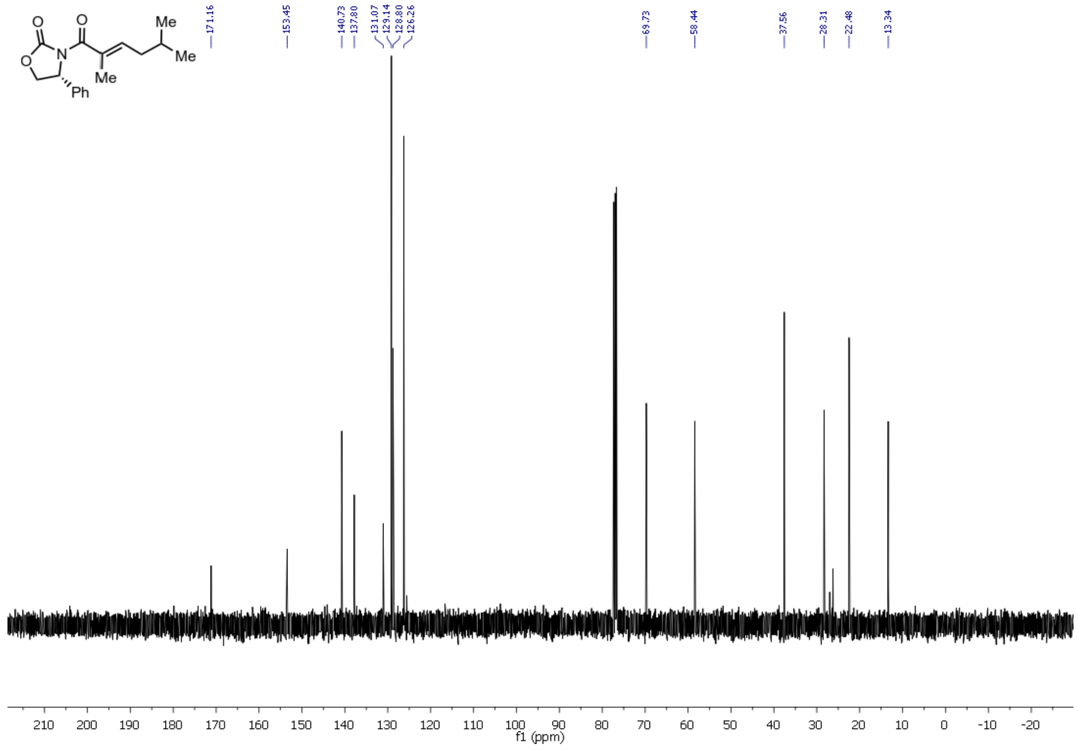

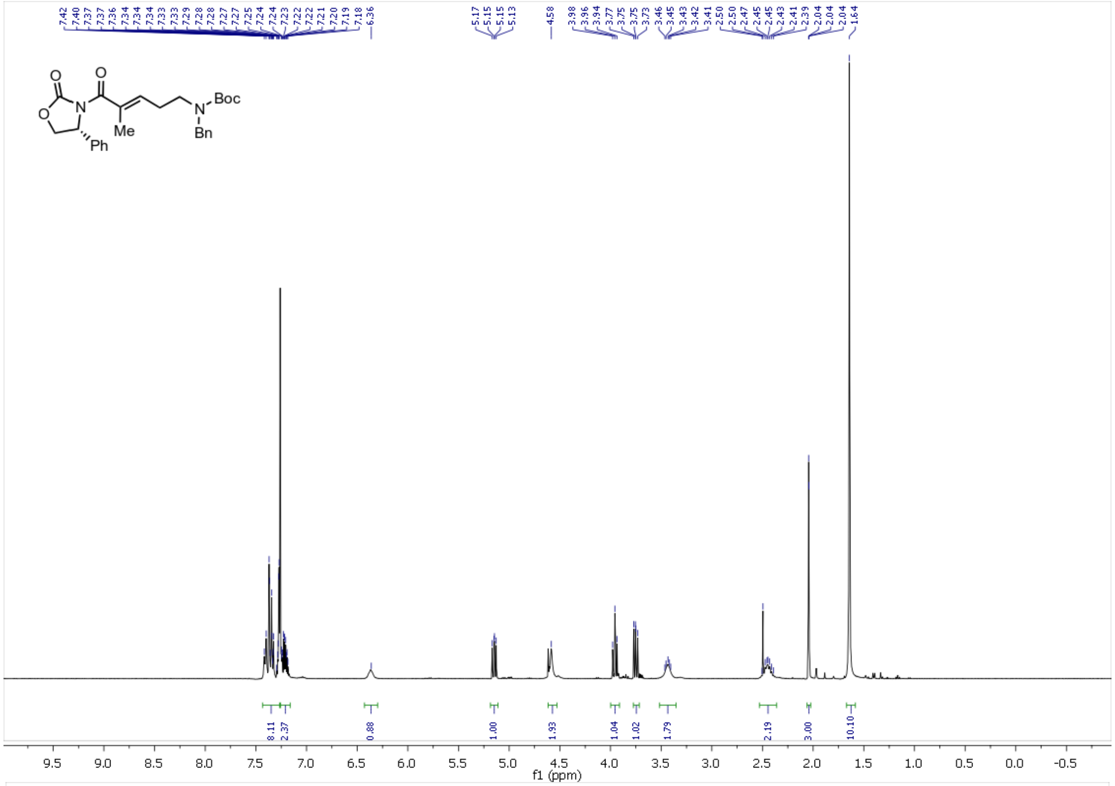
Boc
Bn
Me
Ph
f1 (ppm)

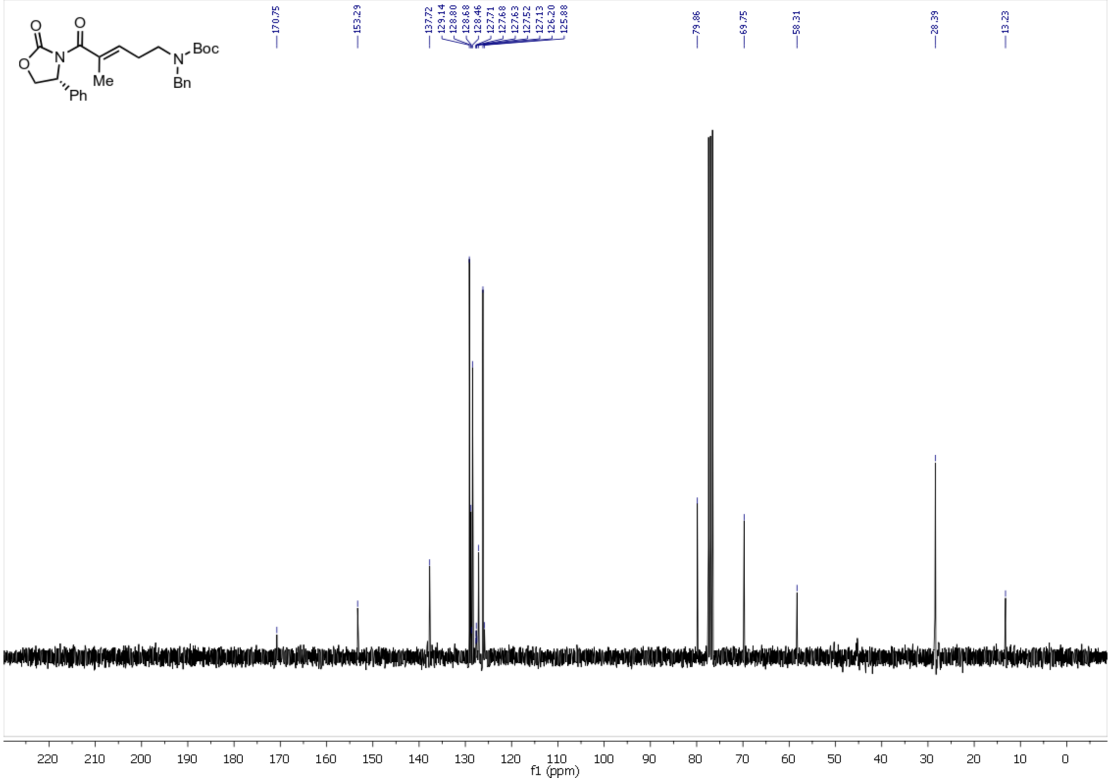
Boc
Bn
Me
Ph
f1 (ppm)

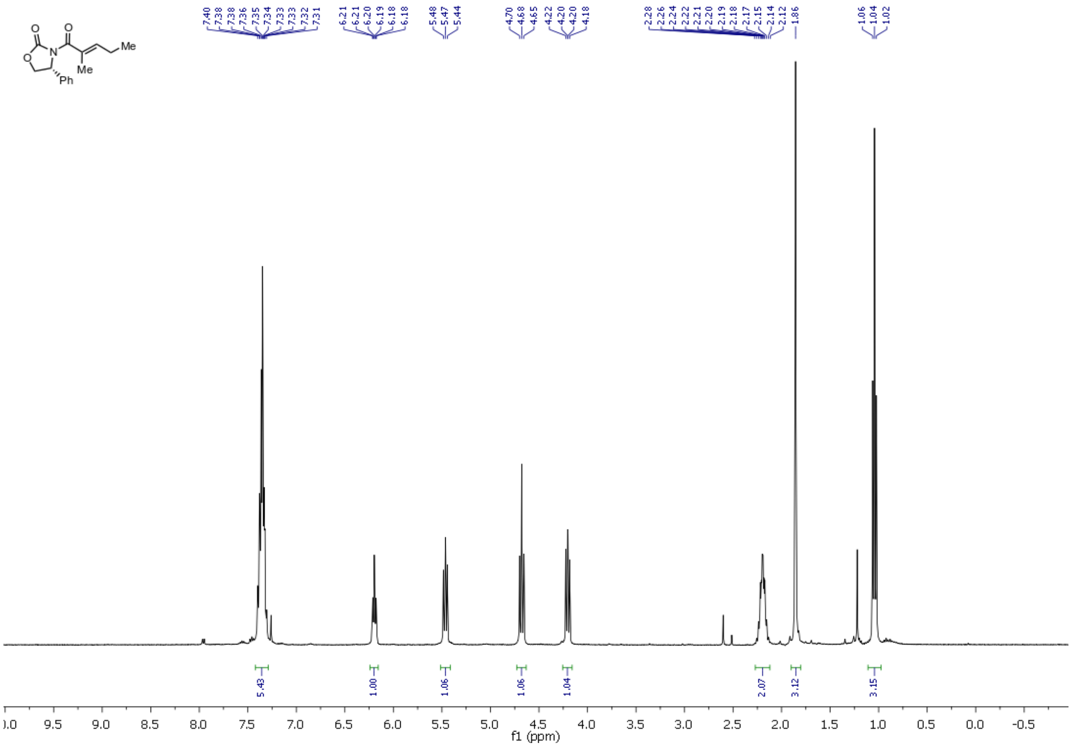

Me
Ph
7.40 7.38 7.38 7.36 7.35 7.34 7.33 7.33 7.32 7.31
6.21 6.21 6.20 6.19 6.18 6.18
5.48 5.47 5.44
4.70 4.68 4.65
4.22 4.20 4.20 4.18
2.28 2.26 2.24 2.22 2.21 2.20 2.19 2.18 2.17 2.15 2.14 2.12
1.86
1.06 1.04 1.02
5.43 1.00 1.06 1.06 1.04 2.07 3.12 3.15
f1 (ppm)

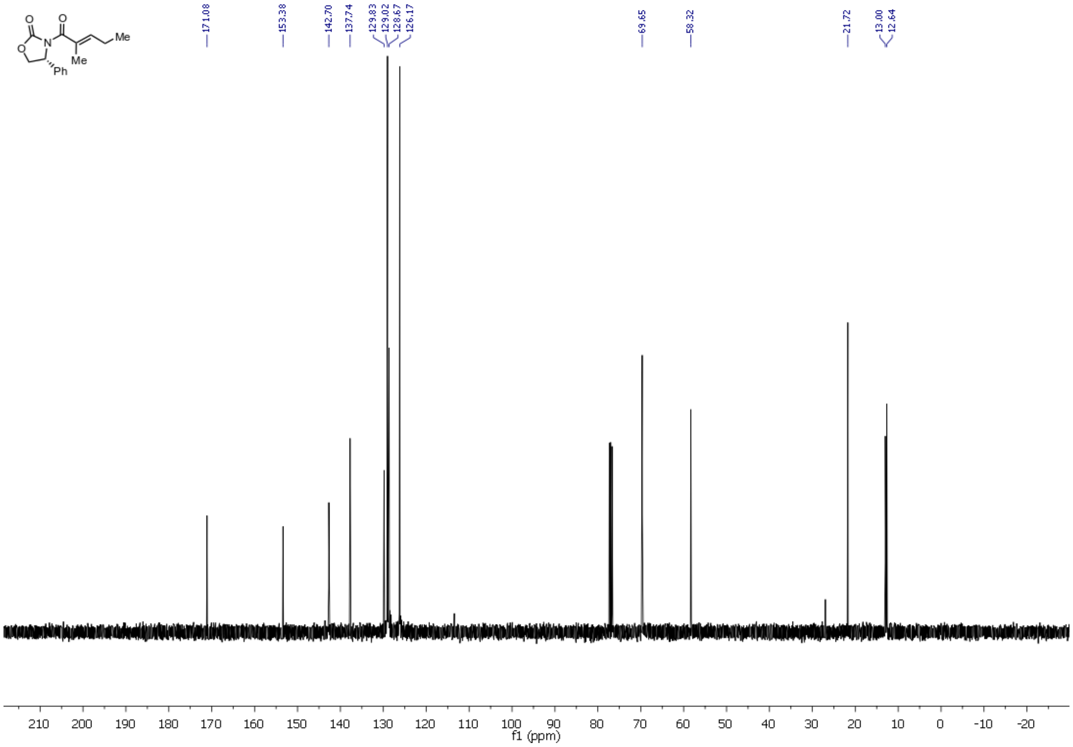

Me
Ph
171.08
153.38
142.70
137.74
129.83 129.02 128.67 126.17
69.65
58.32
21.72
13.00 12.64
f1 (ppm)

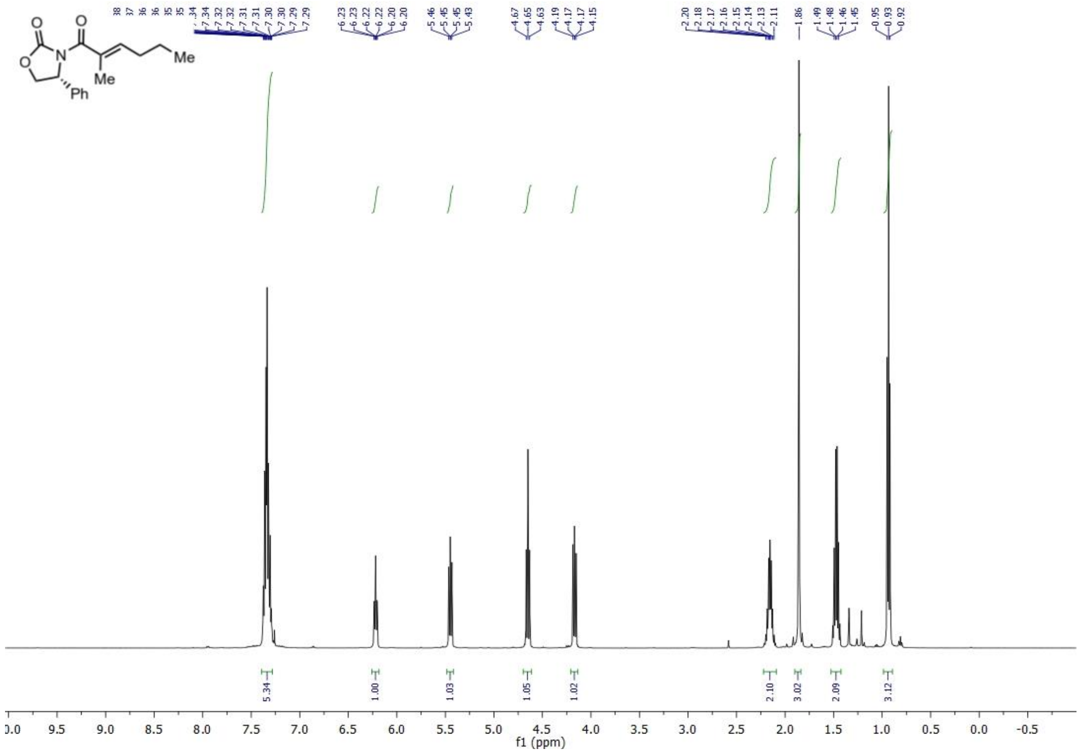

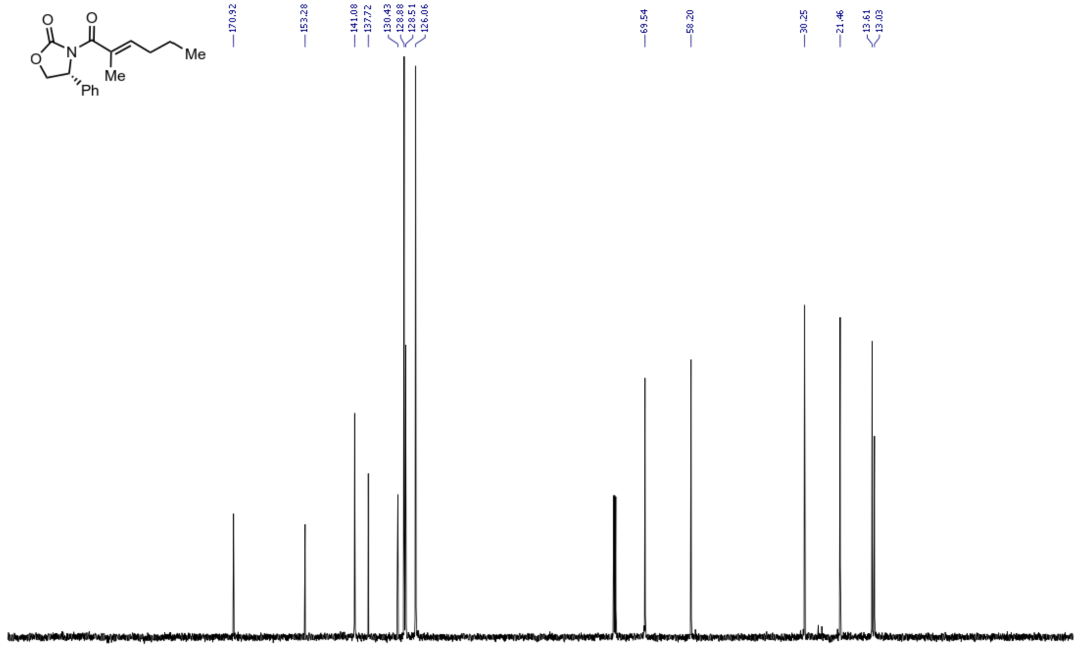

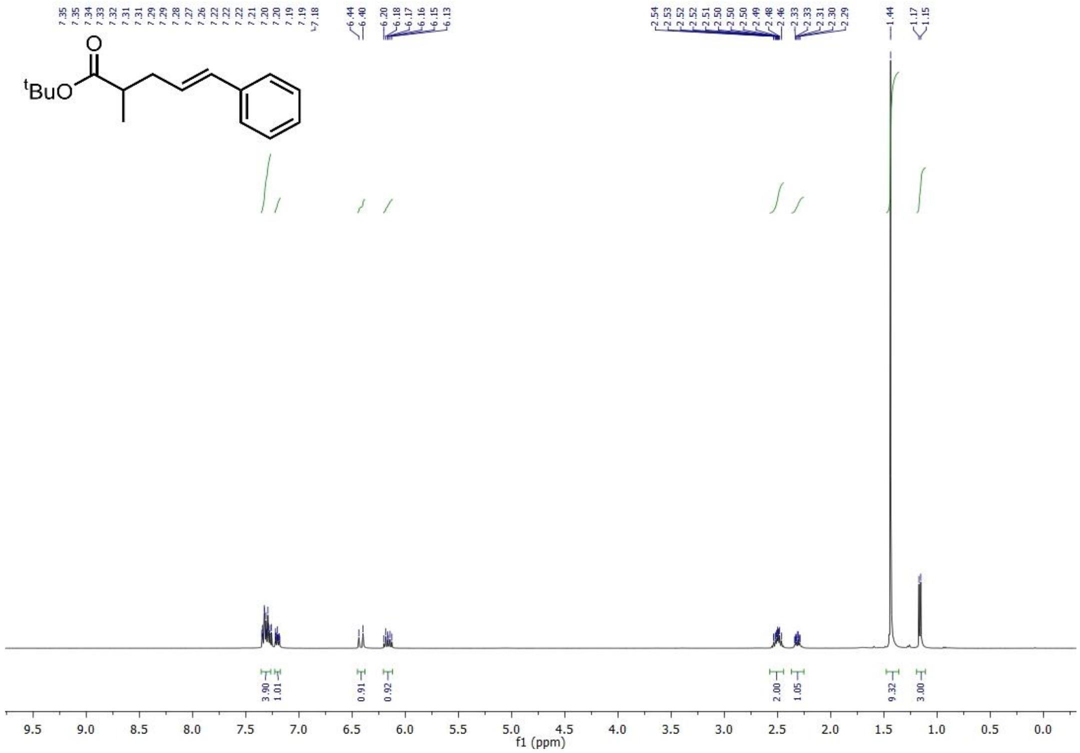

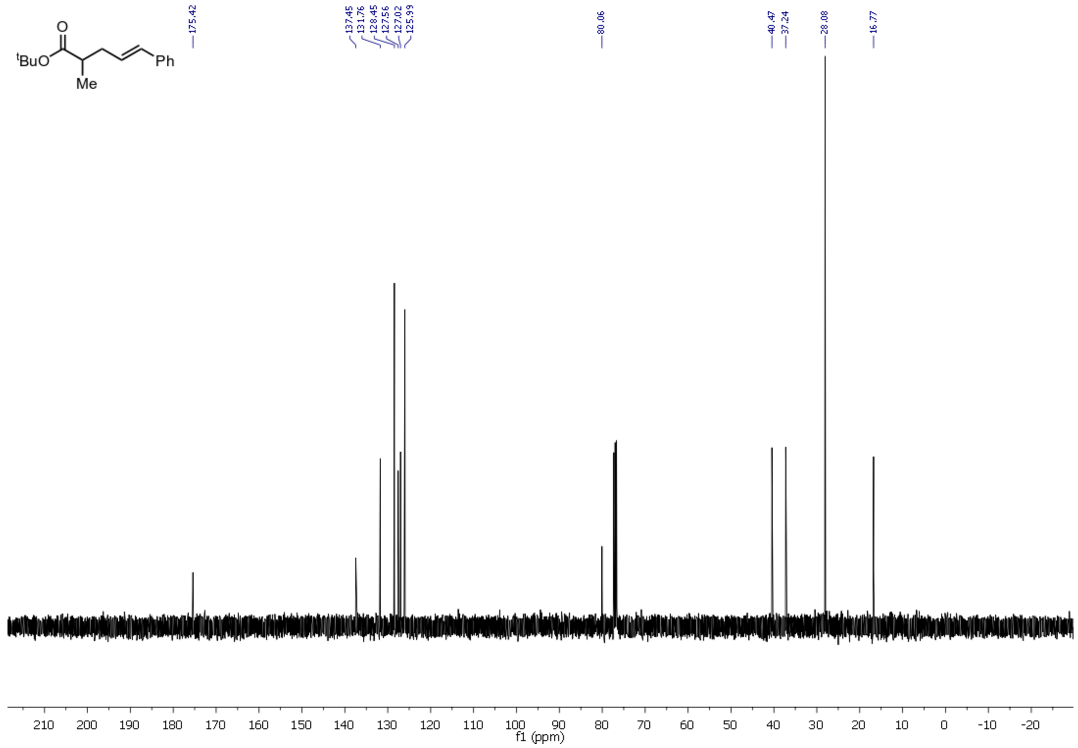

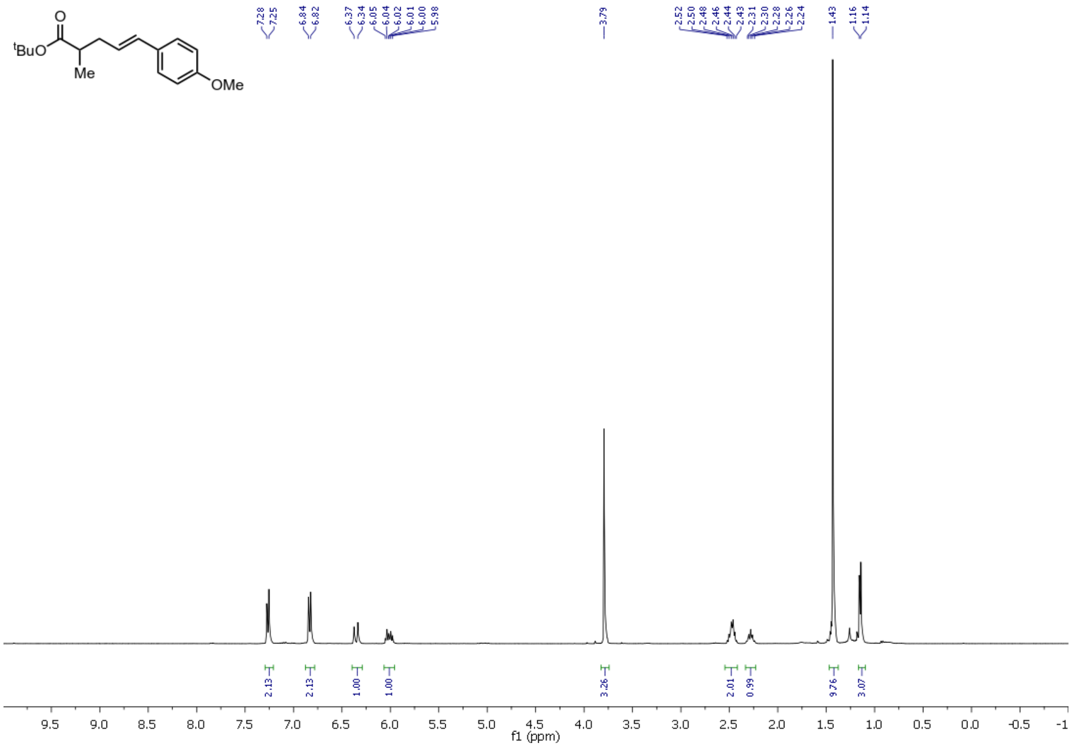
tBuO
O
Me
OMe
7.28
7.25
6.84
6.82
6.37
6.34
6.05
6.04
6.02
6.01
6.00
5.98
3.79
2.52
2.50
2.48
2.46
2.44
2.43
2.31
2.30
2.28
2.26
2.24
1.43
1.16
1.14
2.13
2.13
1.00
1.00
3.26
2.01
0.99
9.76
3.07
f1 (ppm)

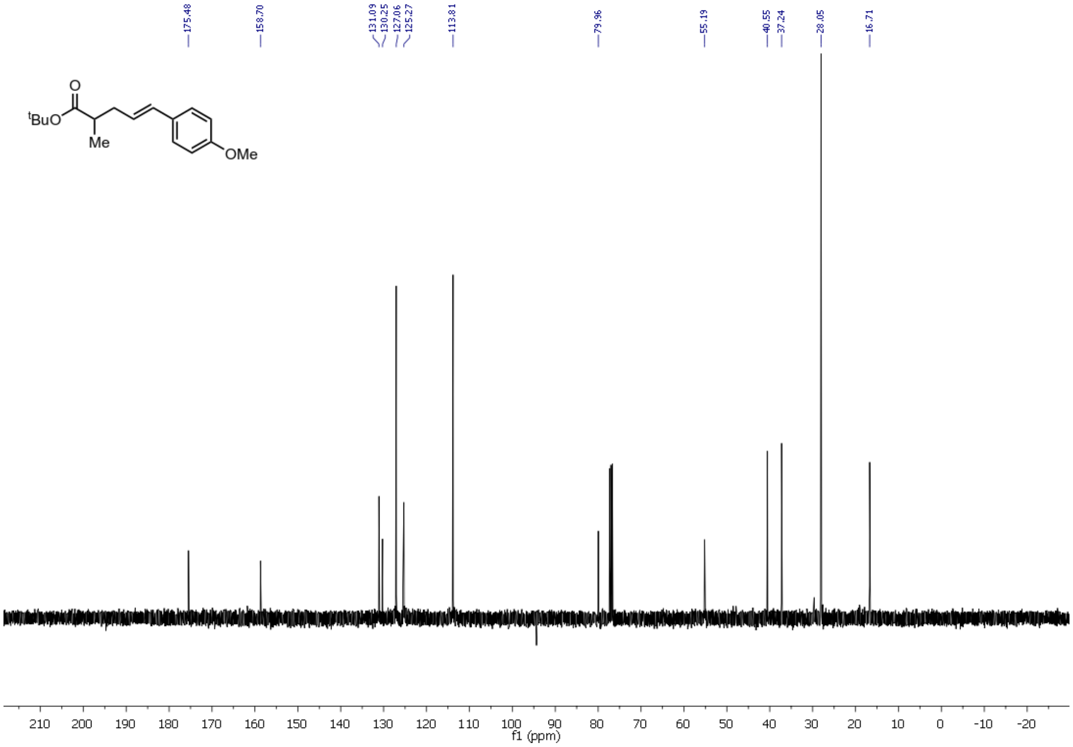
175.48
158.70
131.09
130.25
127.06
125.27
113.81
79.96
55.19
40.55
37.24
28.05
16.71
tBuO
O
Me
OMe
f1 (ppm)

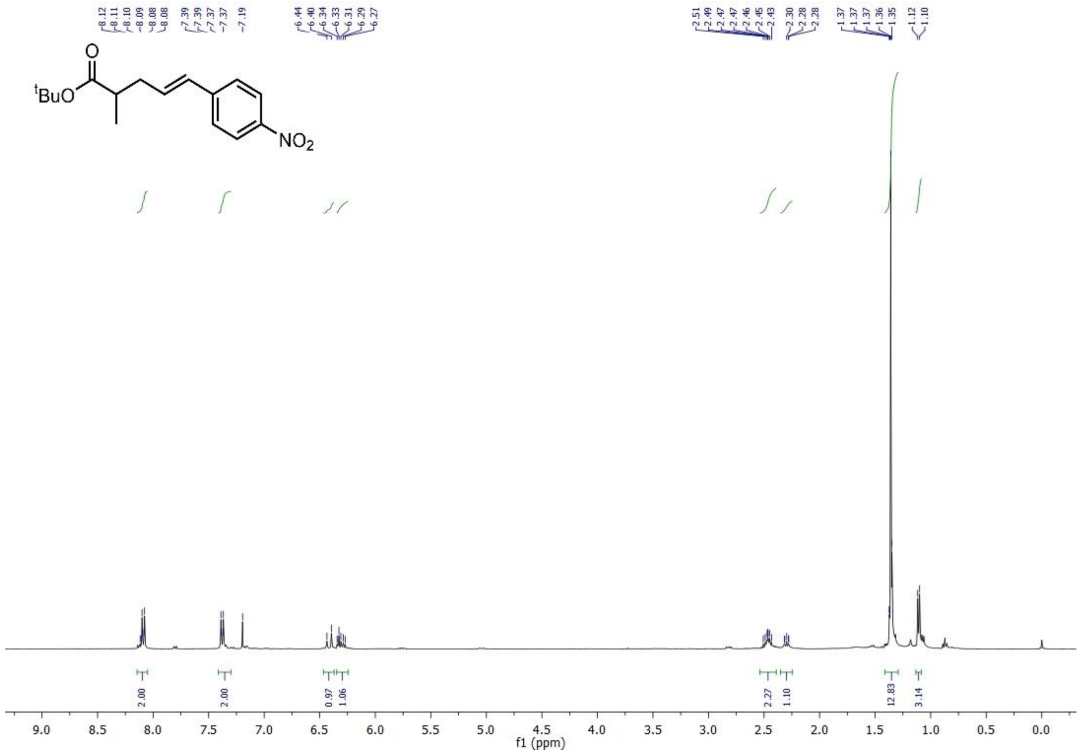

8.12 8.11 8.10 8.09 8.08 8.08 7.39 7.39 7.37 7.37 7.19
6.44 6.40 6.34 6.33 6.31 6.29 6.27
2.51 2.49 2.47 2.47 2.46 2.45 2.43 2.30 2.28 2.28 1.37 1.37 1.37 1.36 1.35 1.12 1.10
^{t}BuO
NO$_2$
2.00
2.00
0.97
1.06
2.27
1.10
12.83
3.14
9.0 8.5 8.0 7.5 7.0 6.5 6.0 5.5 5.0 4.5 4.0 3.5 3.0 2.5 2.0 1.5 1.0 0.5 0.0
f1 (ppm)

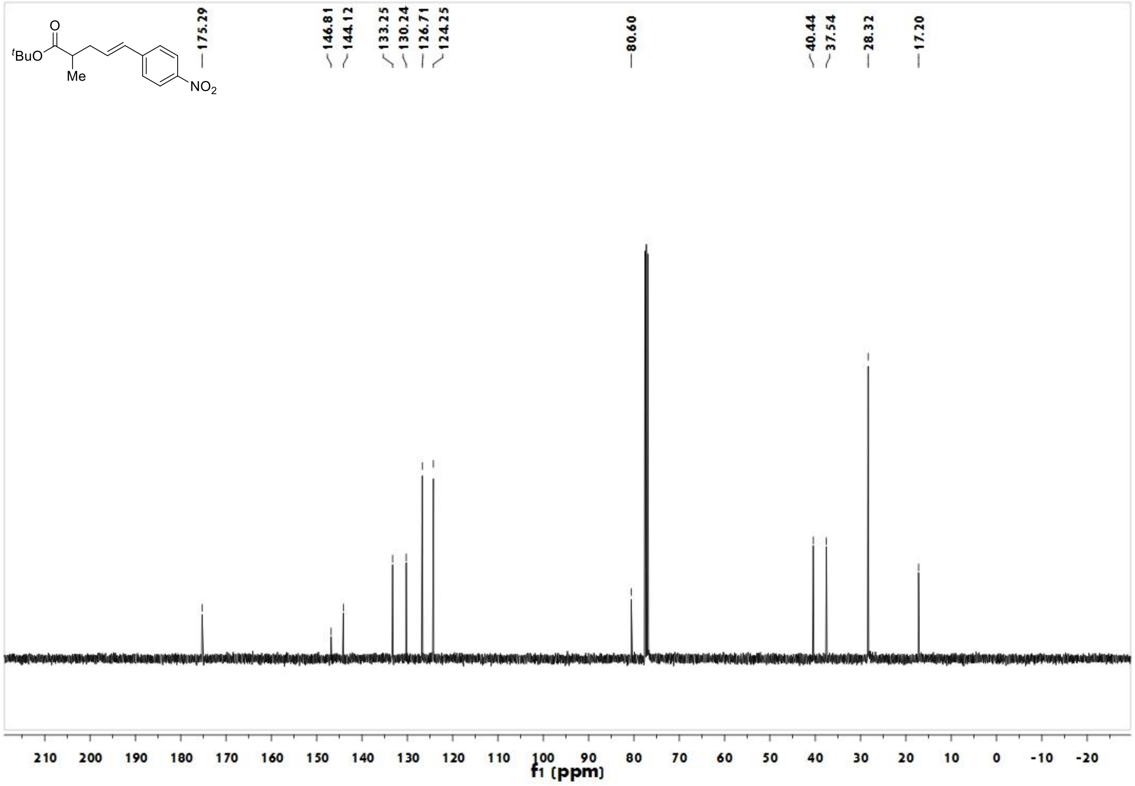

^{t}BuO
Me
NO$_2$
175.29
146.81
144.12
133.25
130.24
126.71
124.25
80.60
40.44
37.54
28.32
17.20
210 200 190 180 170 160 150 140 130 120 110 100 90 80 70 60 50 40 30 20 10 0 -10 -20
f1 (ppm)

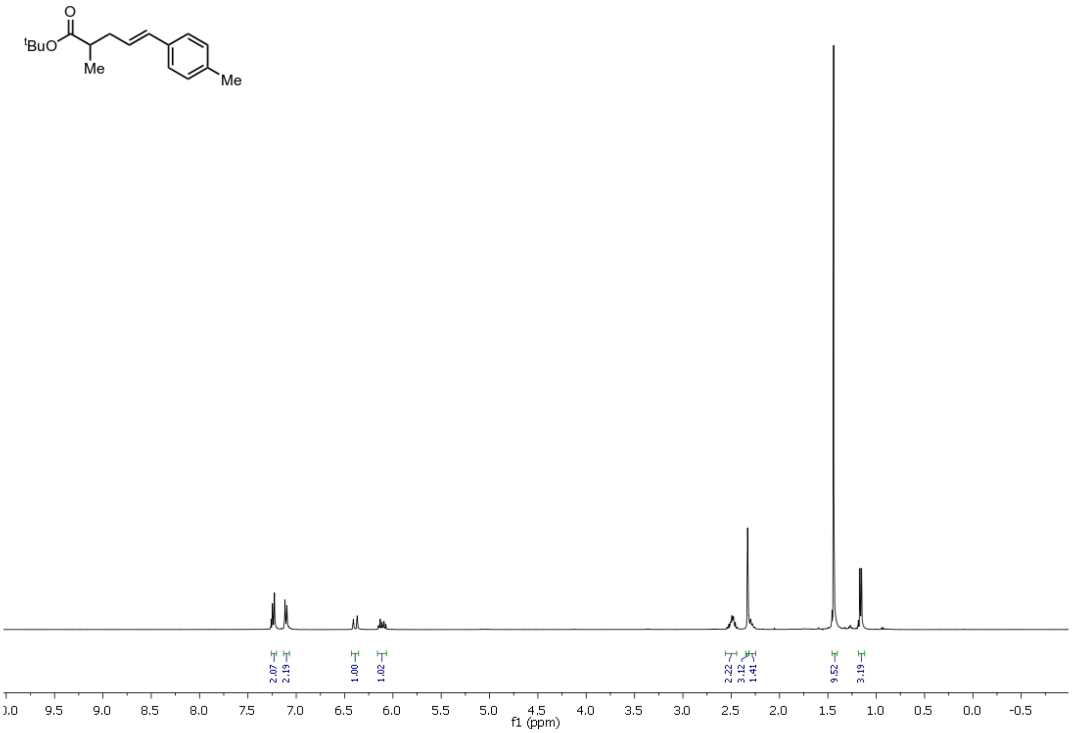

tBuO
O
Me
Me
2.07
2.19
1.00
1.02
2.22
3.12
1.41
9.52
3.19
f1 (ppm)

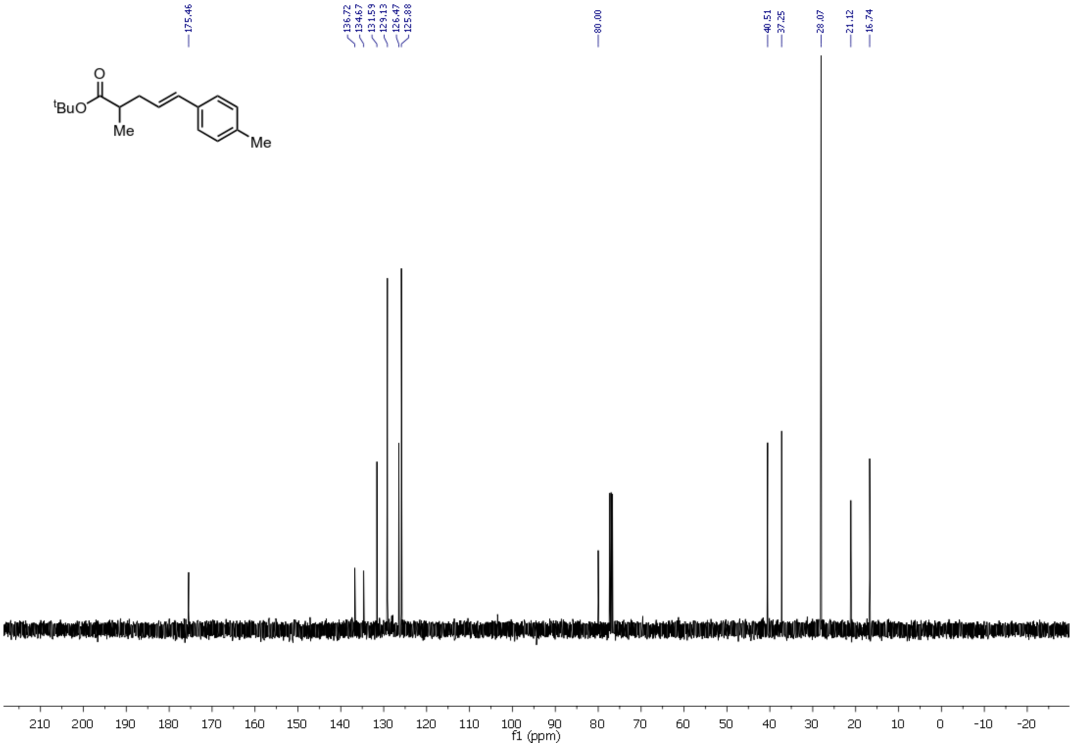

175.46
136.72
134.67
131.59
129.13
126.47
125.88
80.00
40.51
37.25
28.07
21.12
16.74
tBuO
O
Me
Me
f1 (ppm)

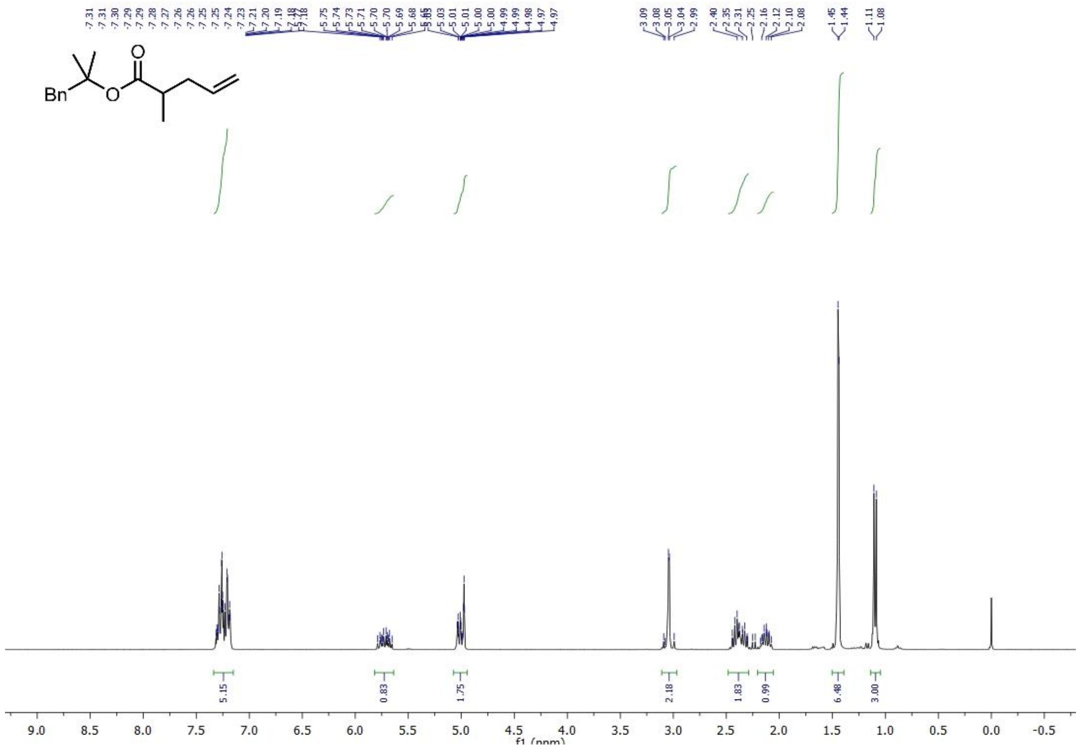
Bn
O
O
5.15
0.83
1.75
2.18
1.83
0.99
6.48
3.00
f1 (ppm)

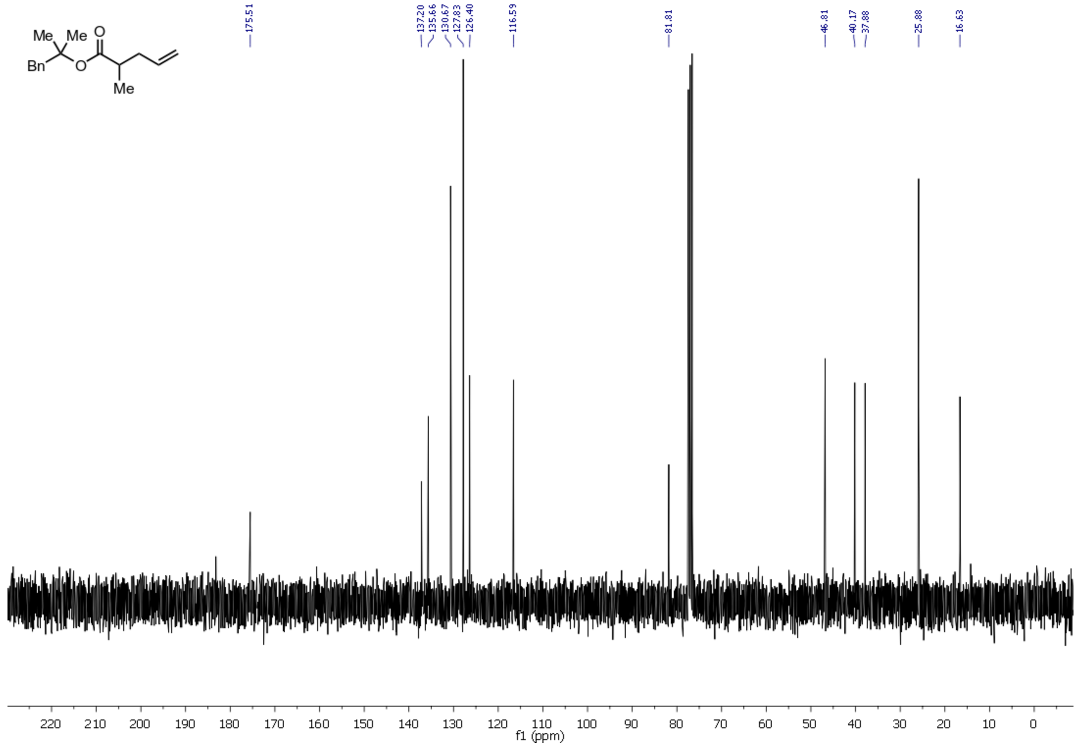
175.51
137.20
135.66
130.67
127.83
126.40
116.59
81.81
46.81
40.17
37.88
25.88
16.63
Me
Me
O
Bn
O
Me
f1 (ppm)

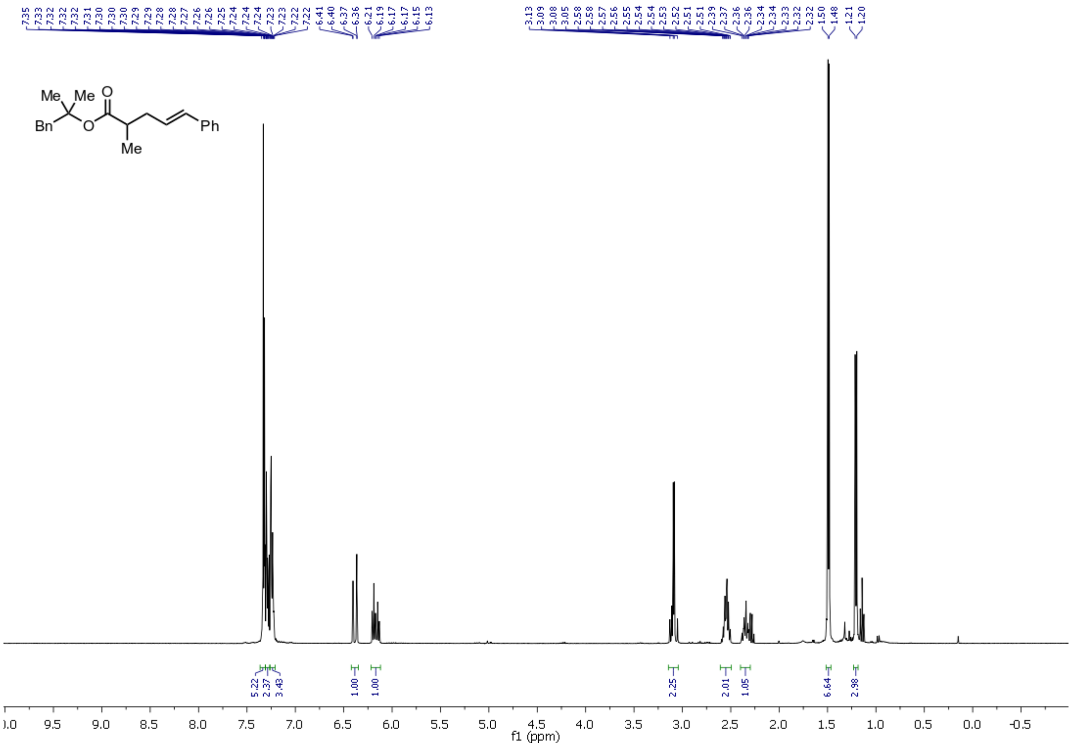

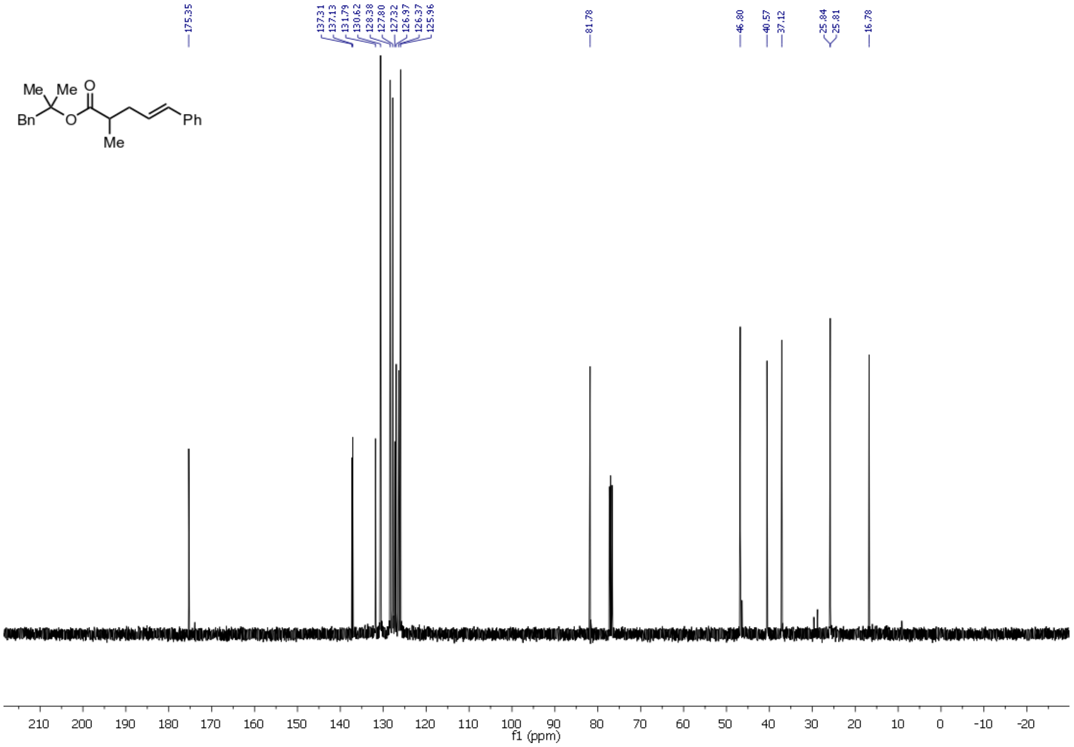

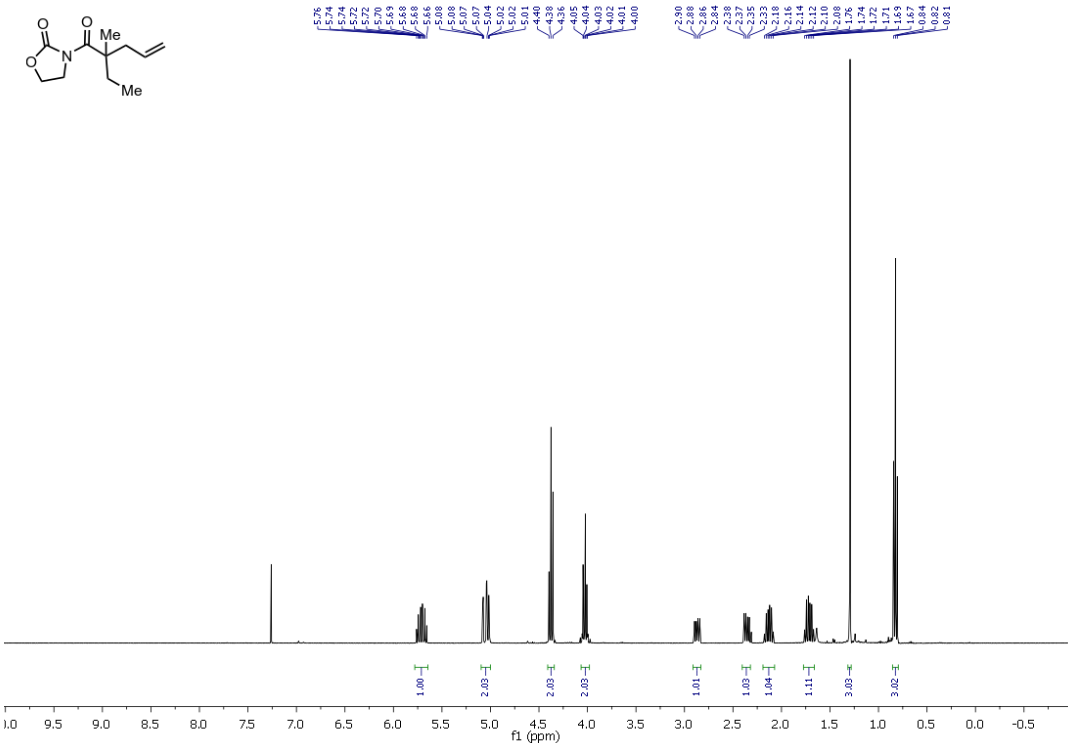

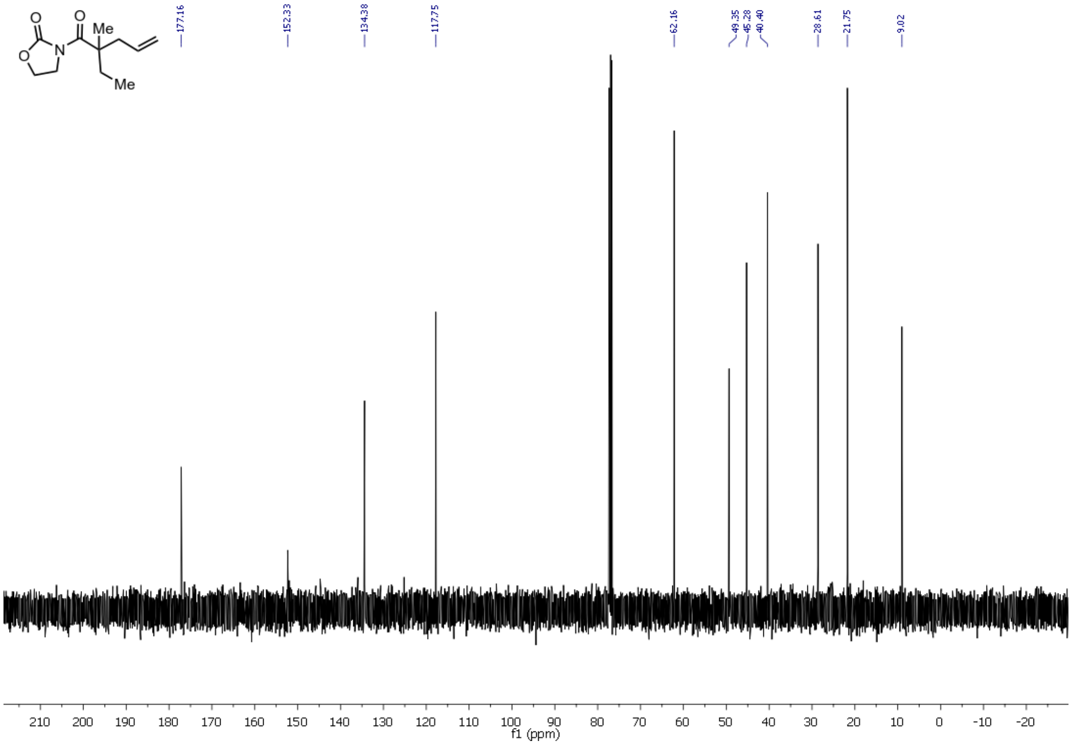

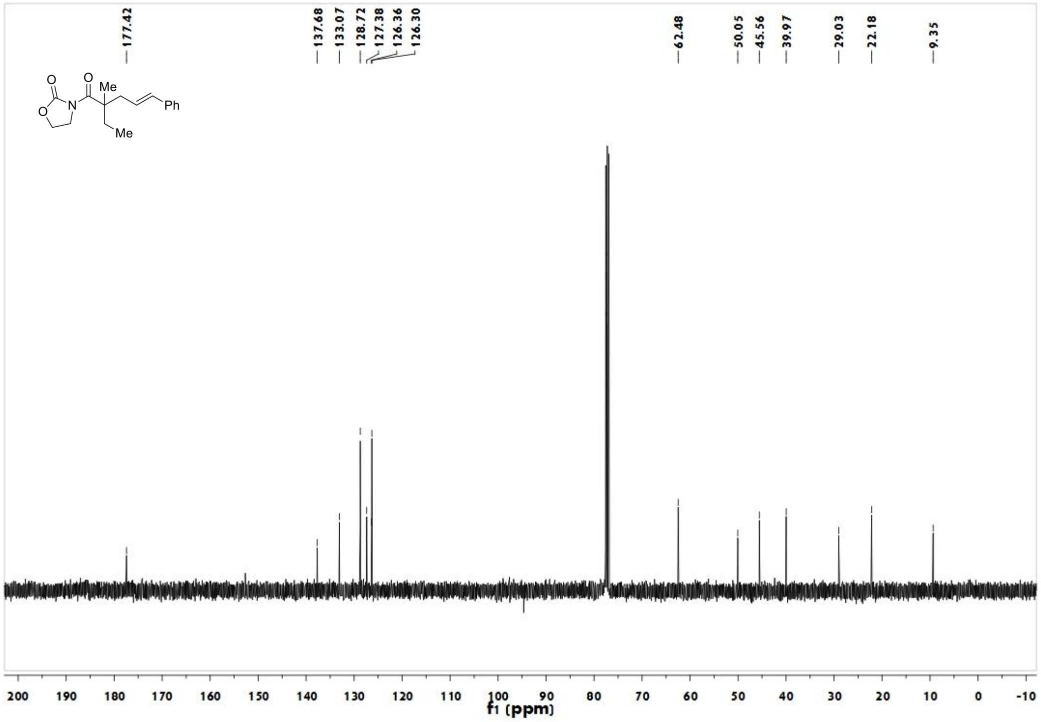

177.42
137.68
133.07
128.72
127.38
126.36
126.30
62.48
50.05
45.56
39.97
29.03
22.18
9.35
Me
Ph
Me
f1 (ppm)

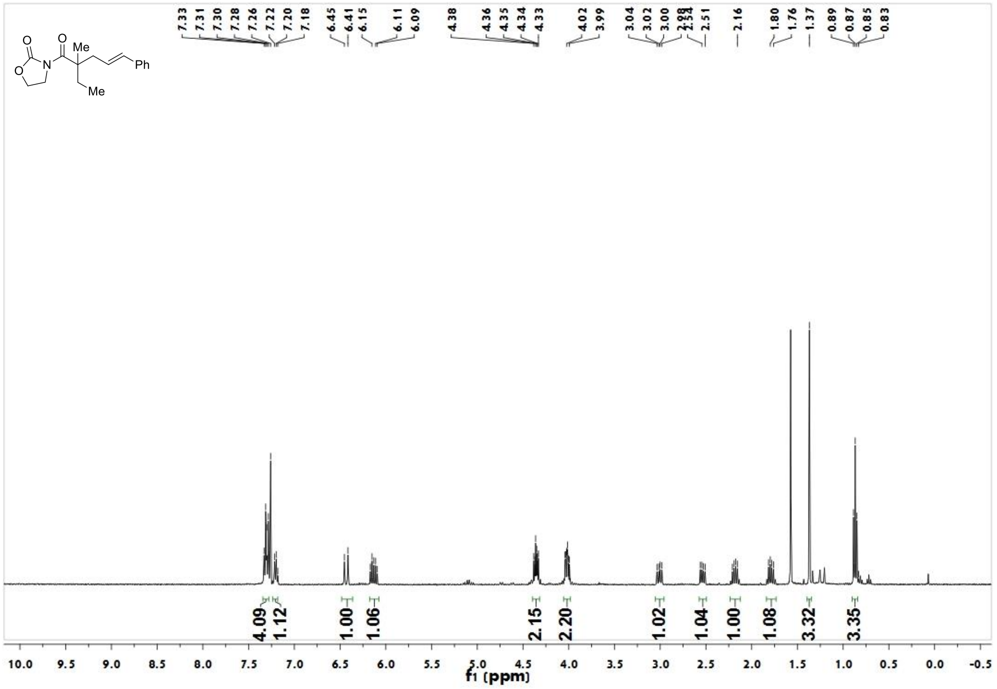

Me
Ph
Me
4.09
1.12
1.00
1.06
2.15
2.20
1.02
1.04
1.00
1.08
3.32
3.35
f1 (ppm)

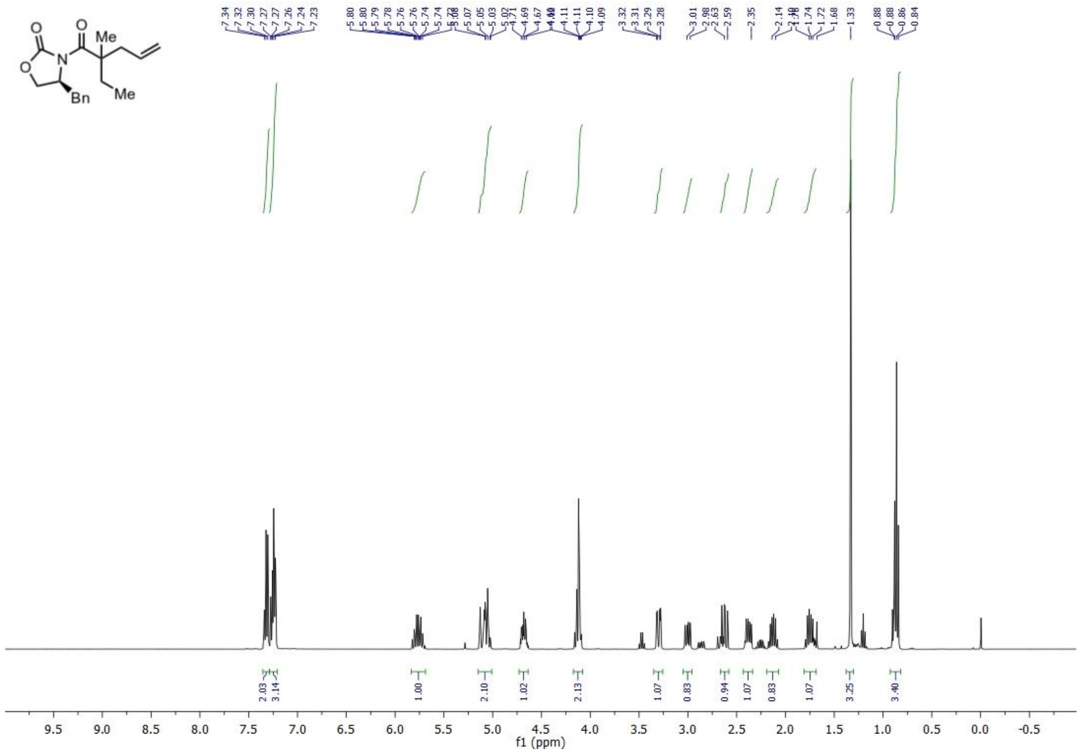
Me
Bn
Me
f1 (ppm)

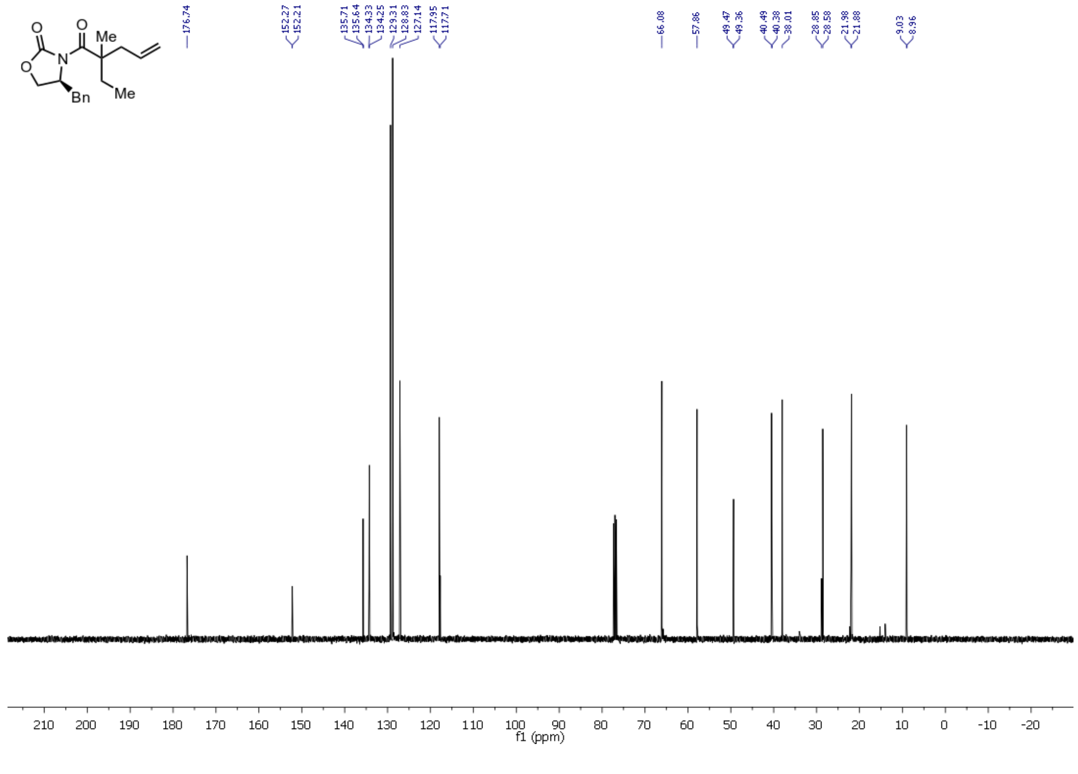
Me
Bn
Me
f1 (ppm)

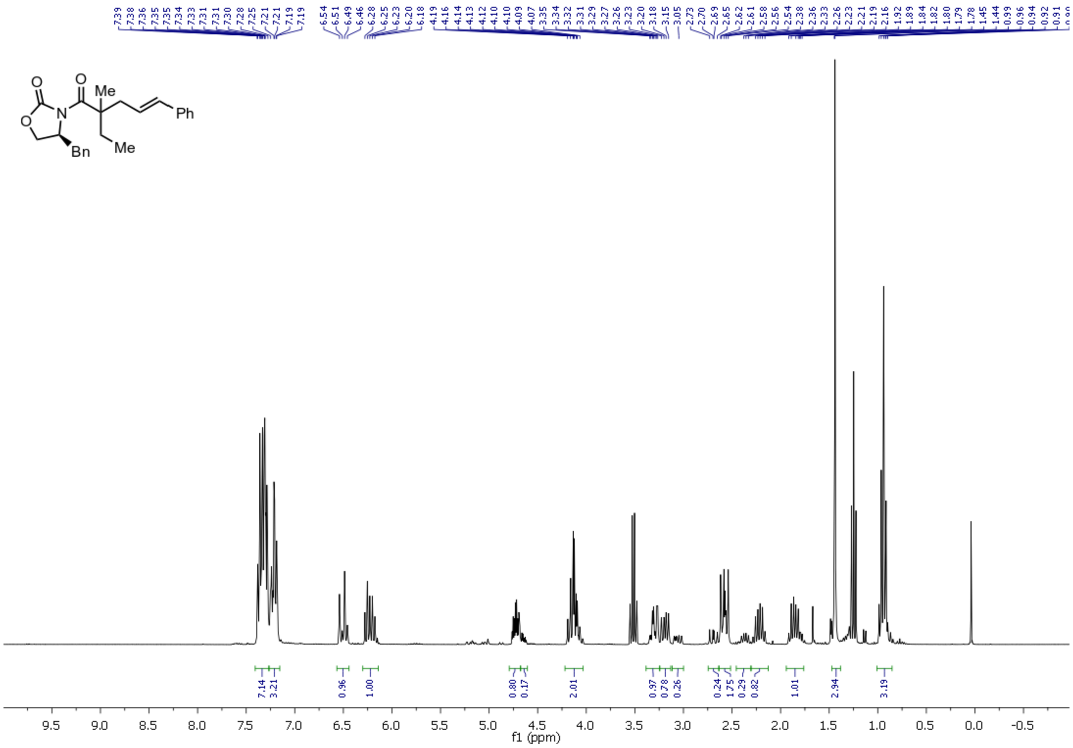
O
O
Me
N
Ph
O
Bn
Me
f1 (ppm)

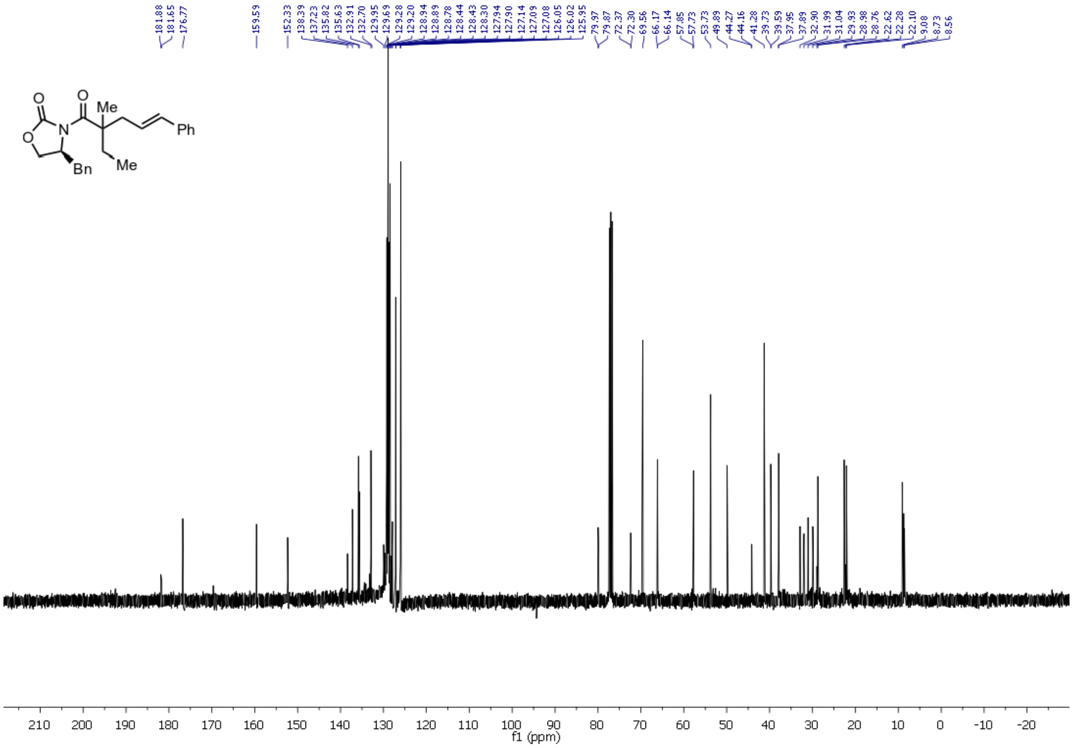
O
O
Me
N
Ph
O
Bn
Me
f1 (ppm)

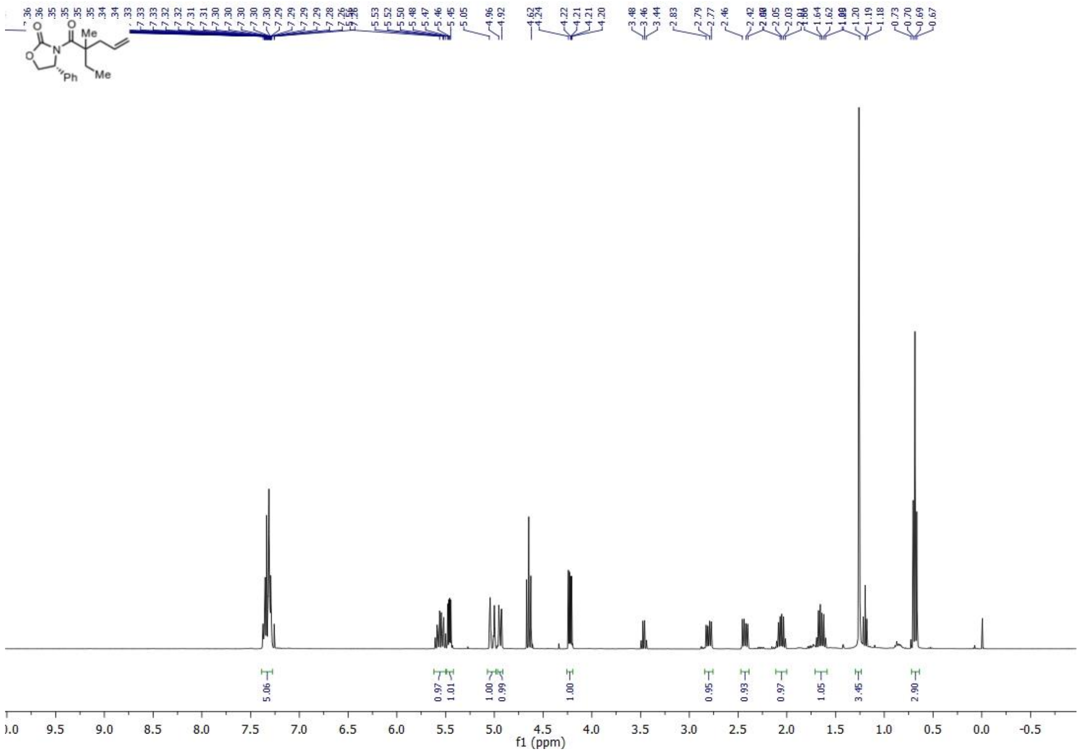

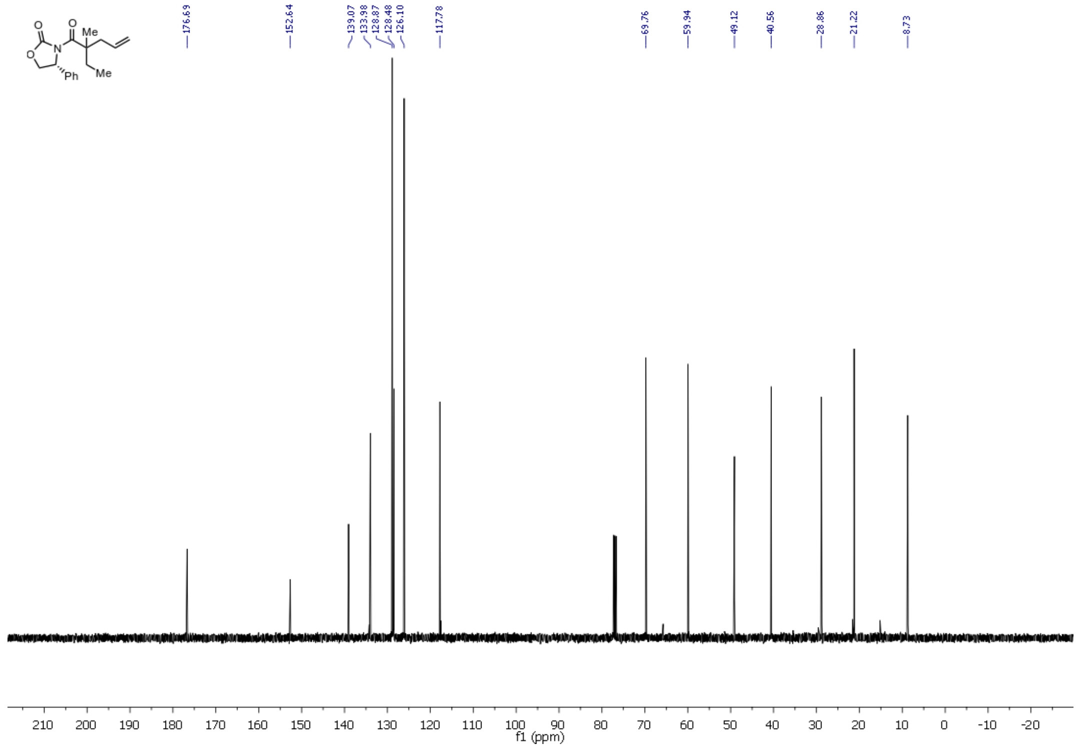

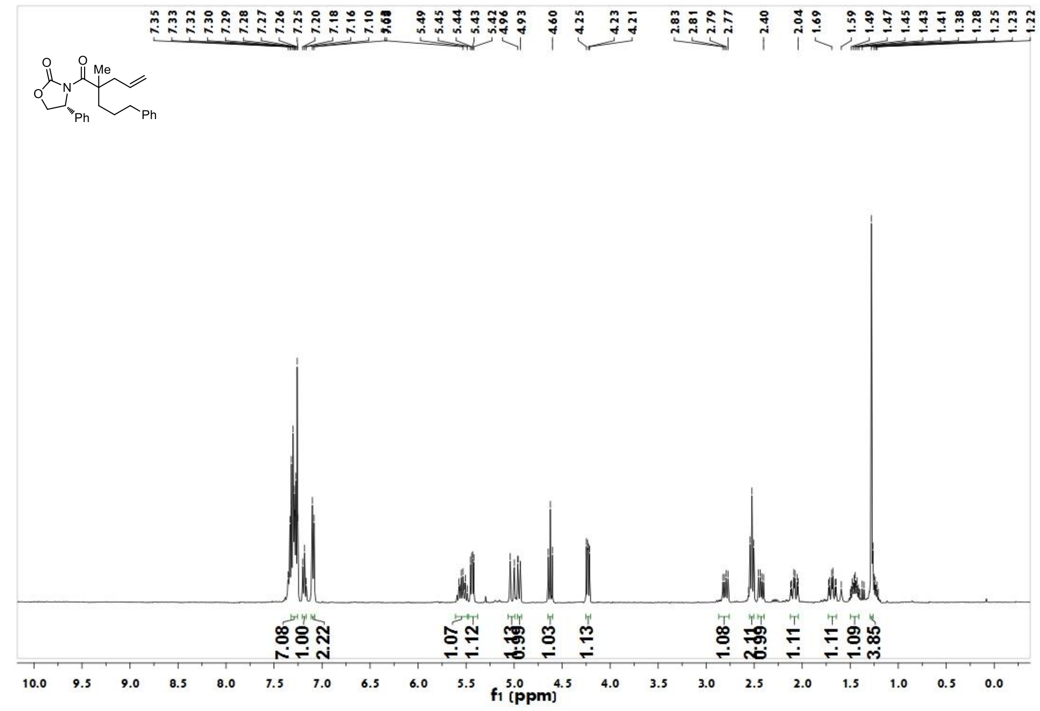

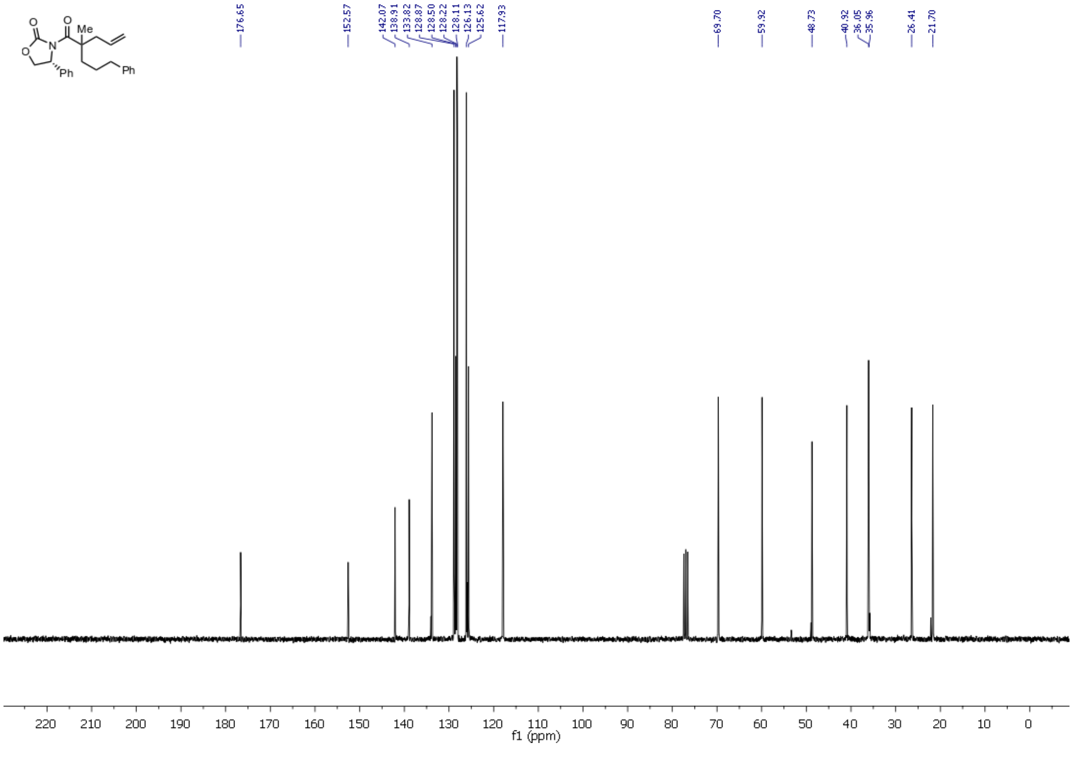

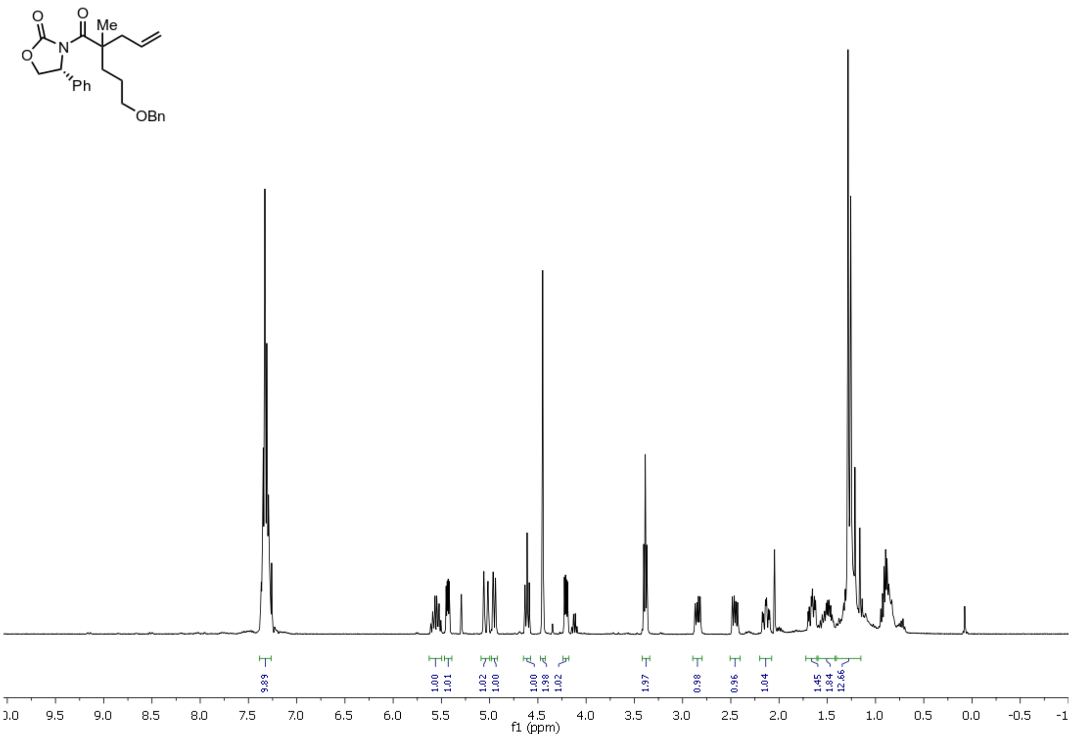

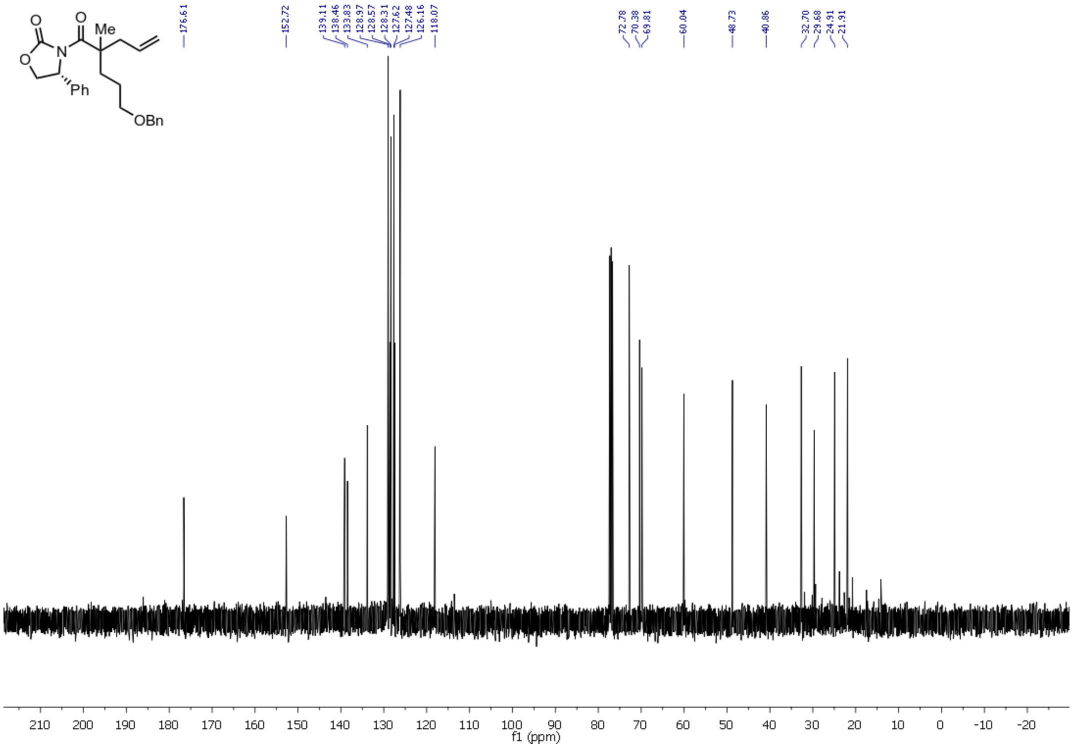

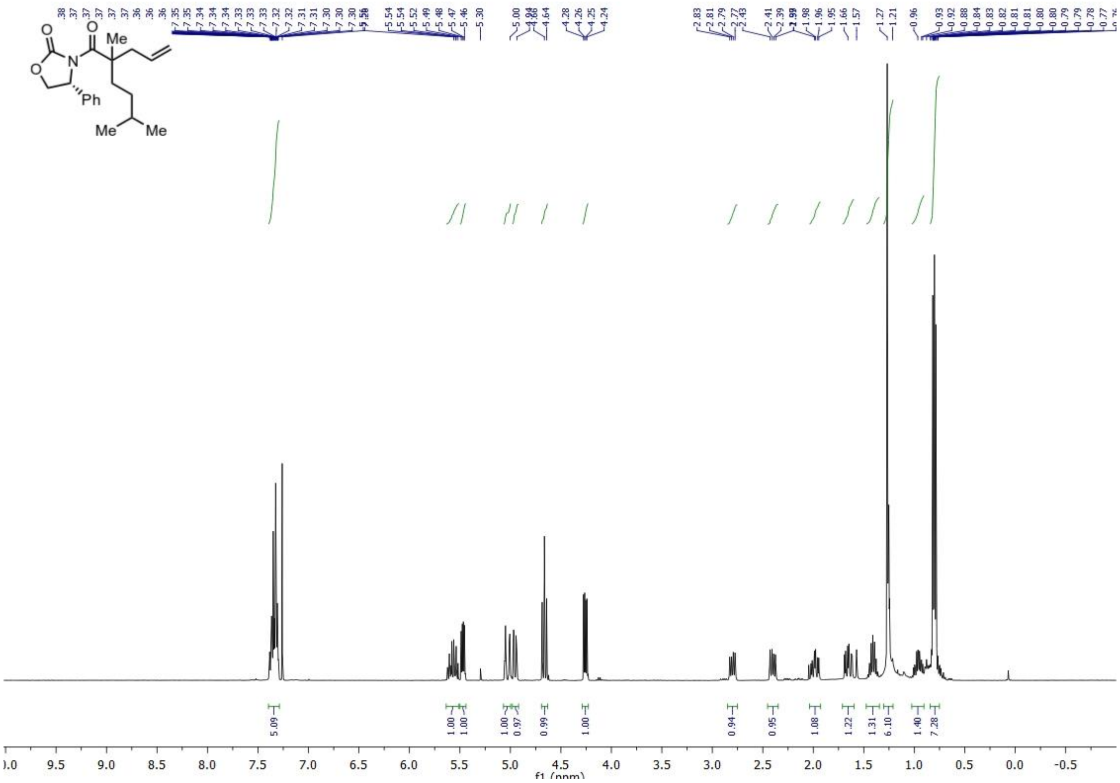

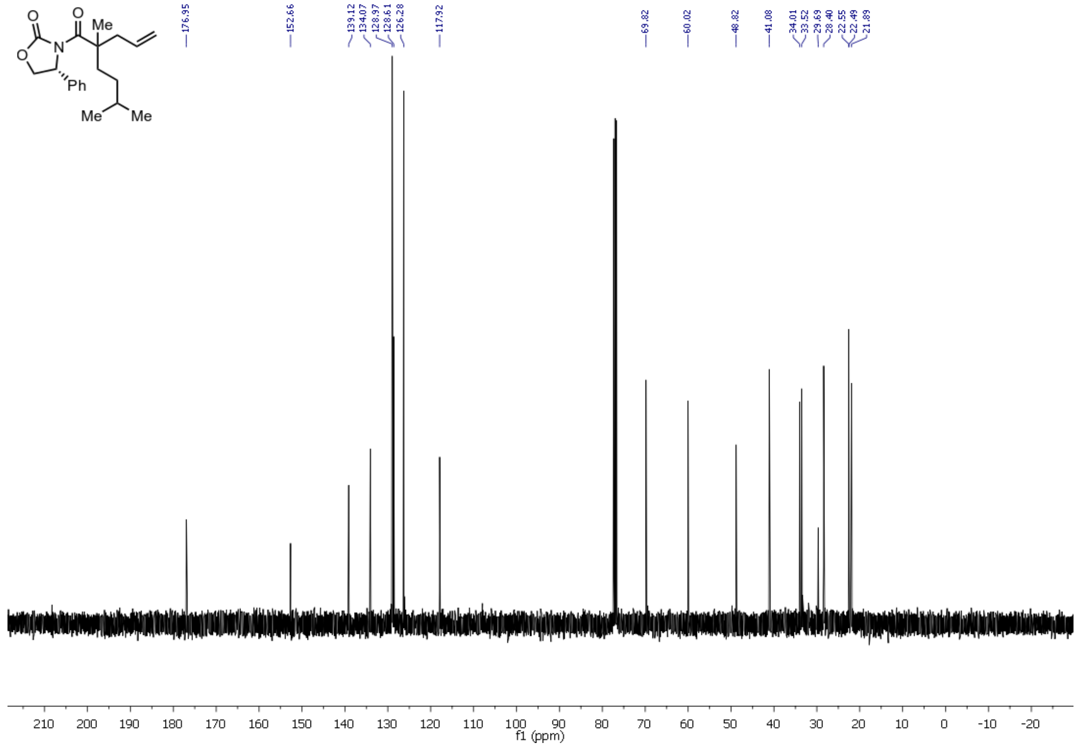

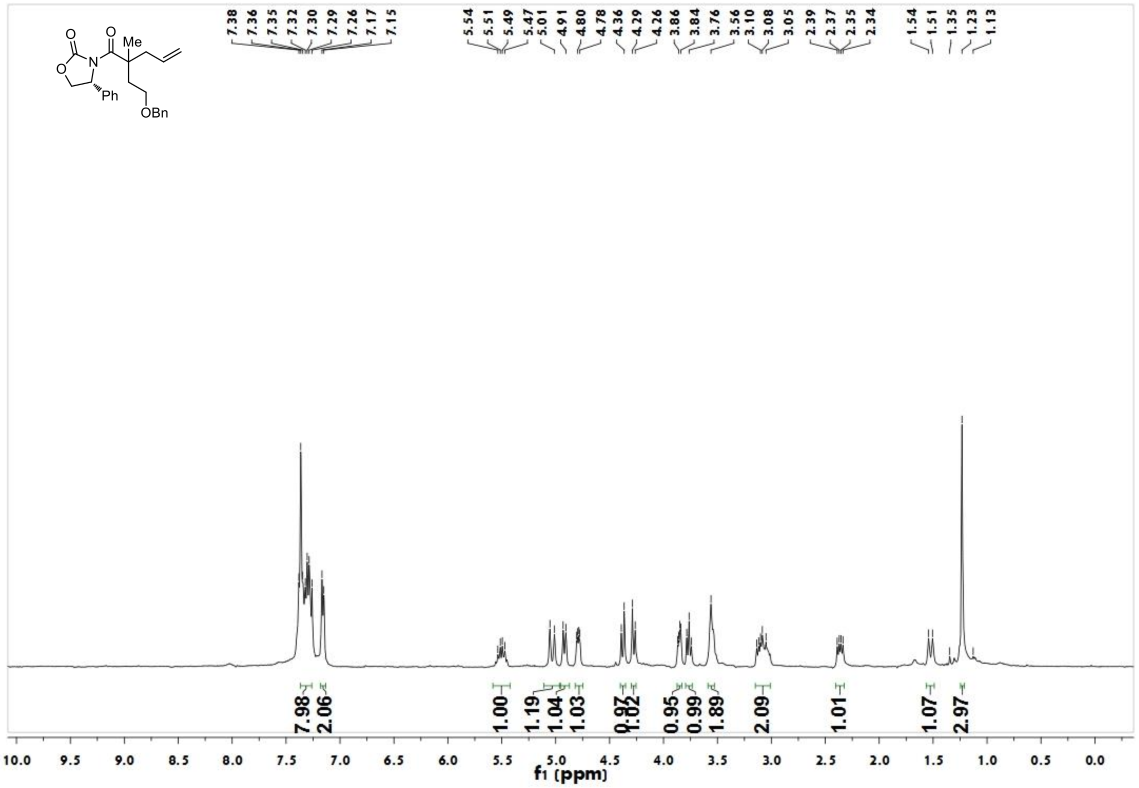

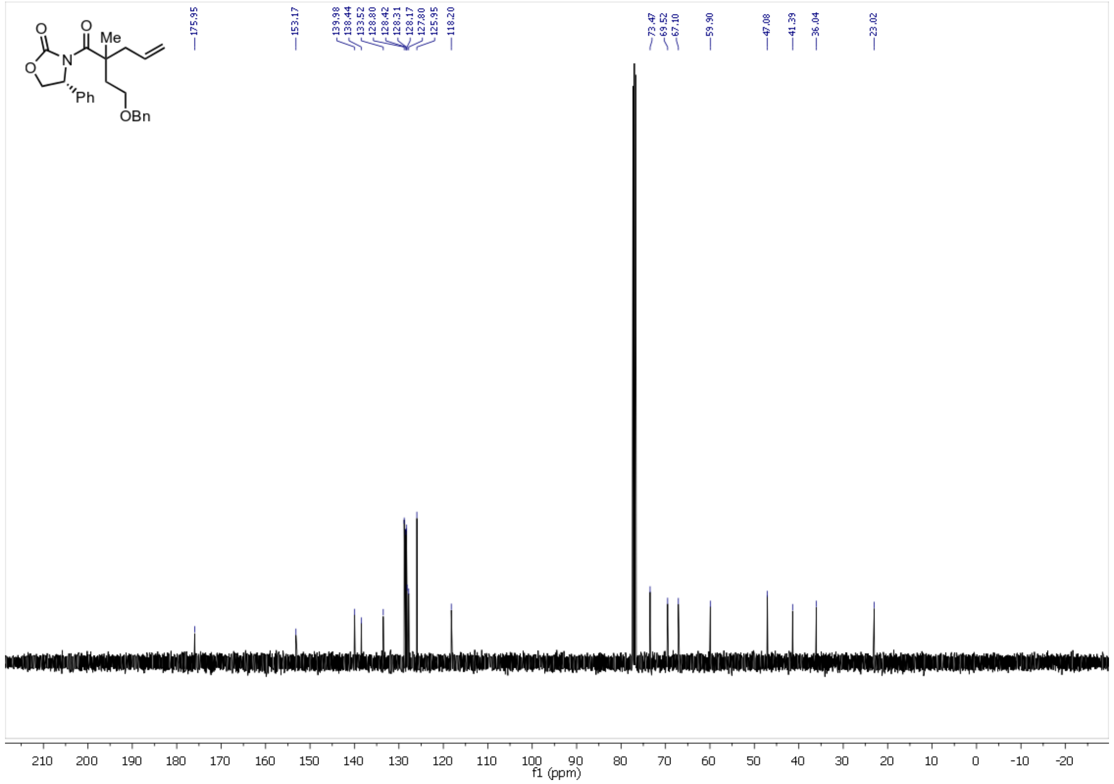

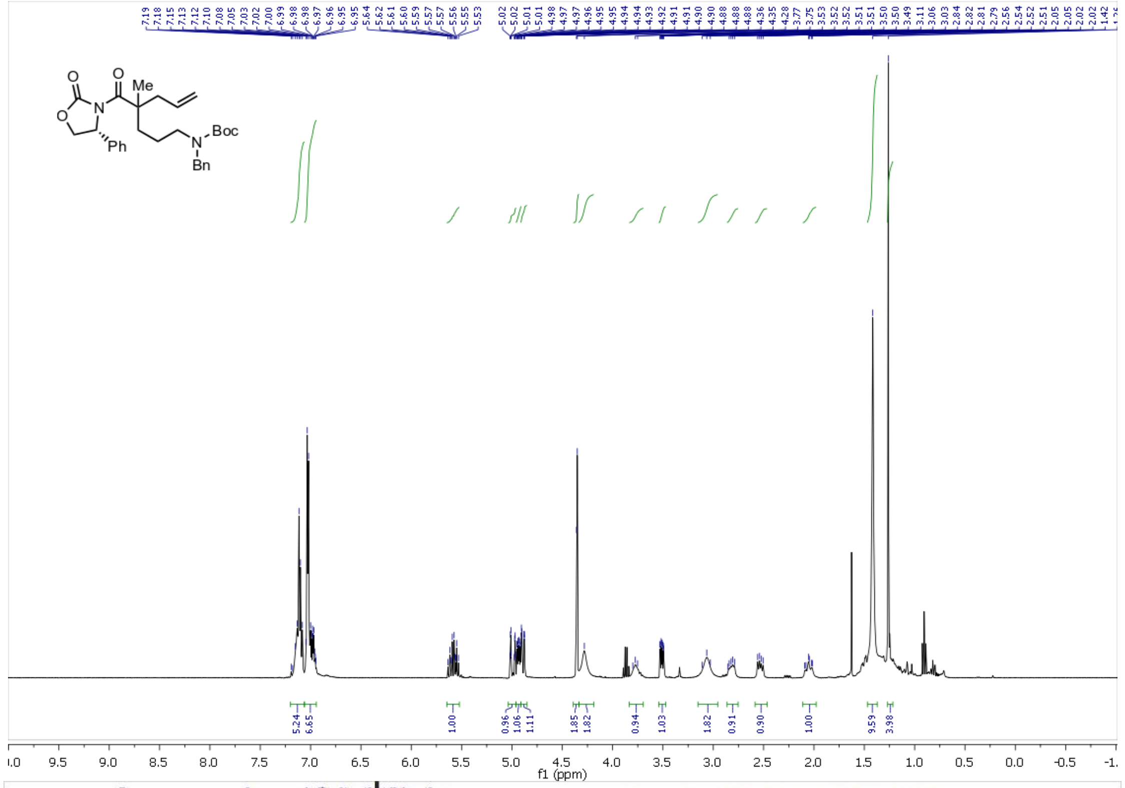

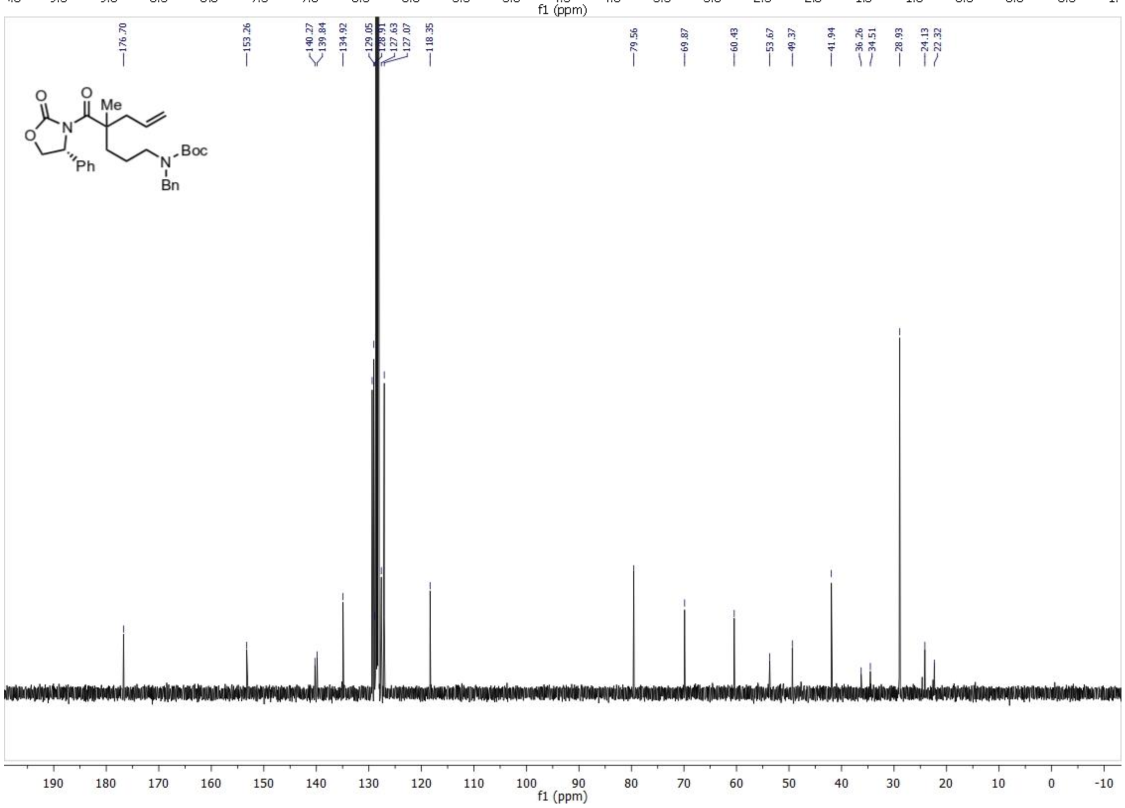

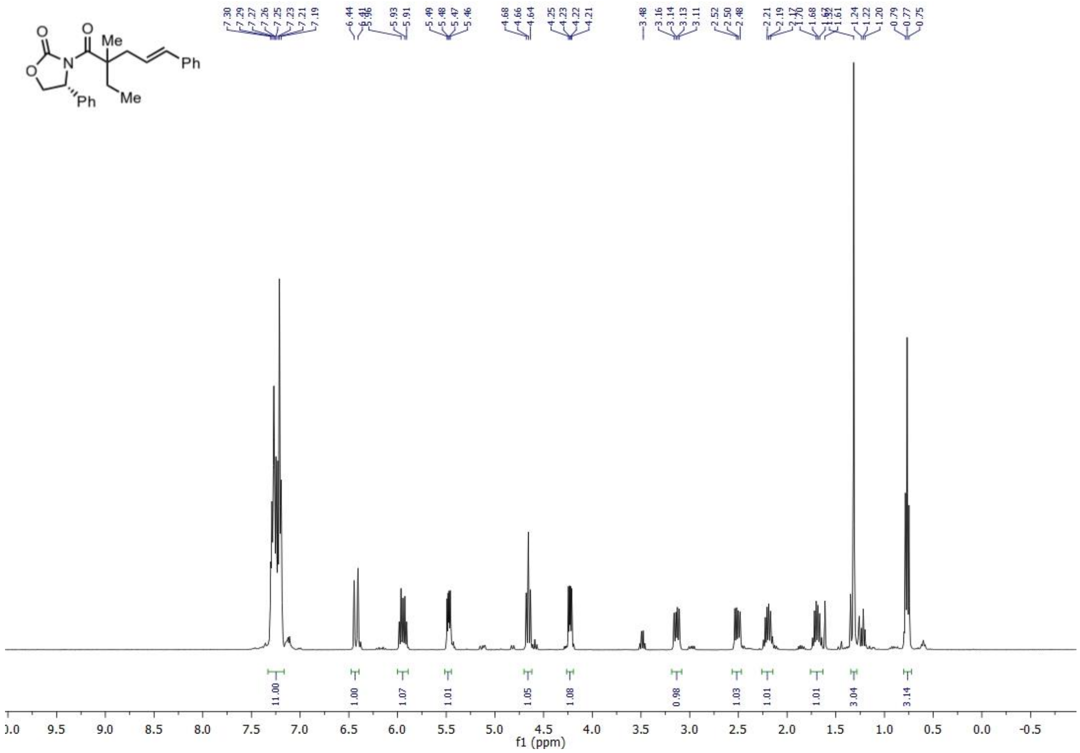

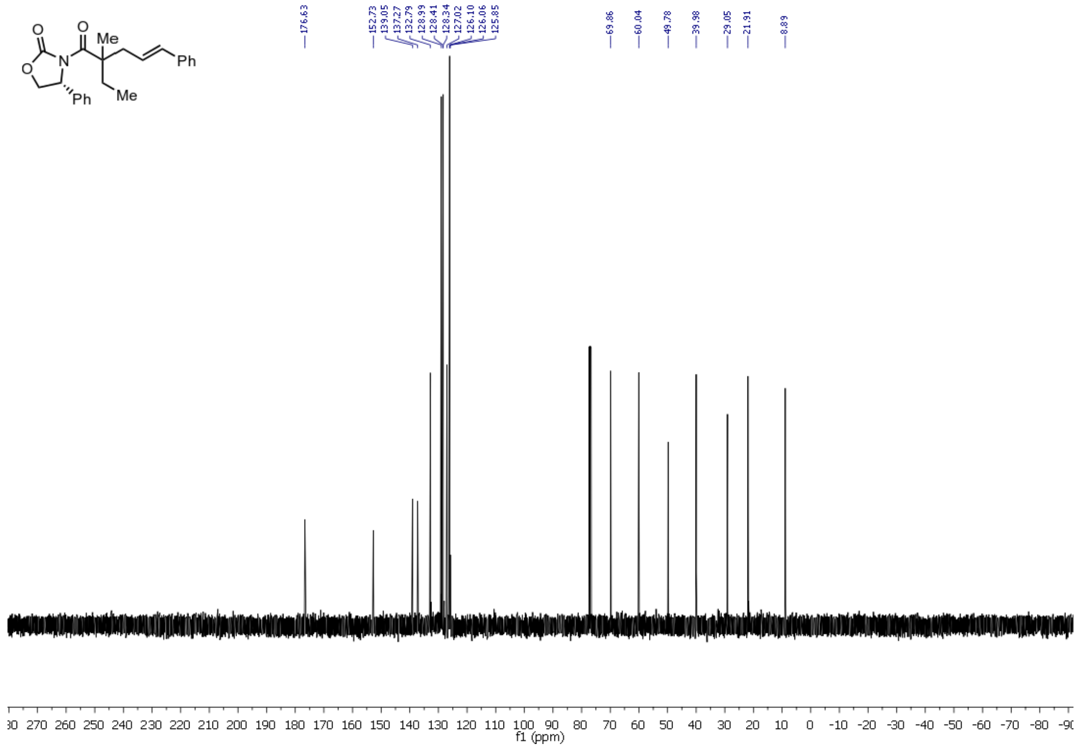

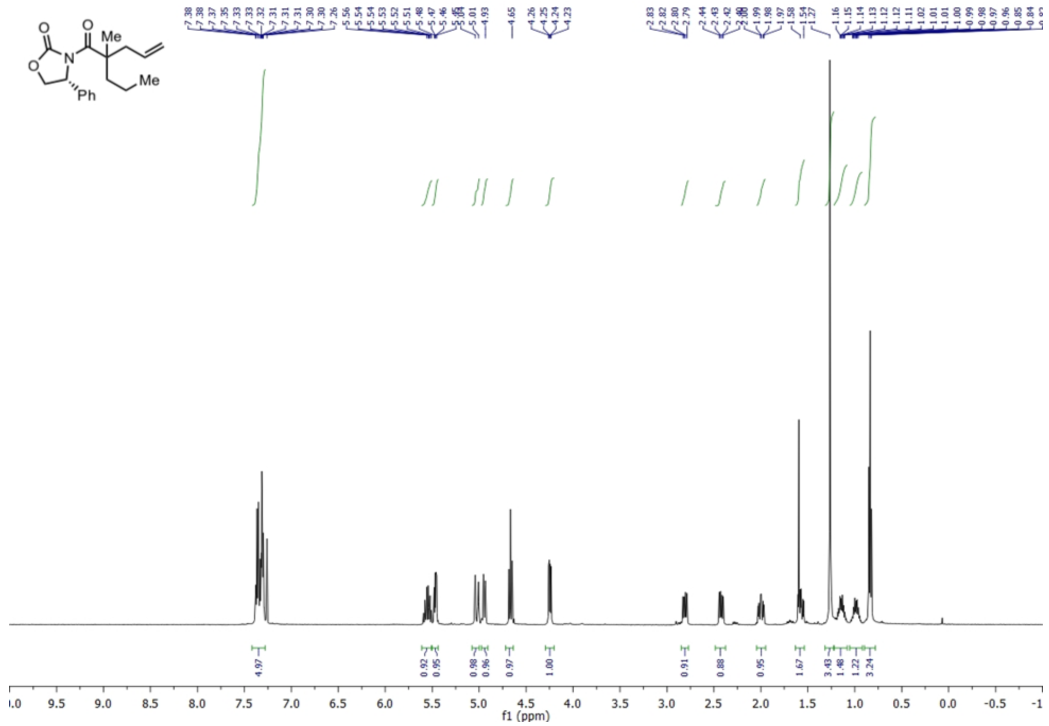

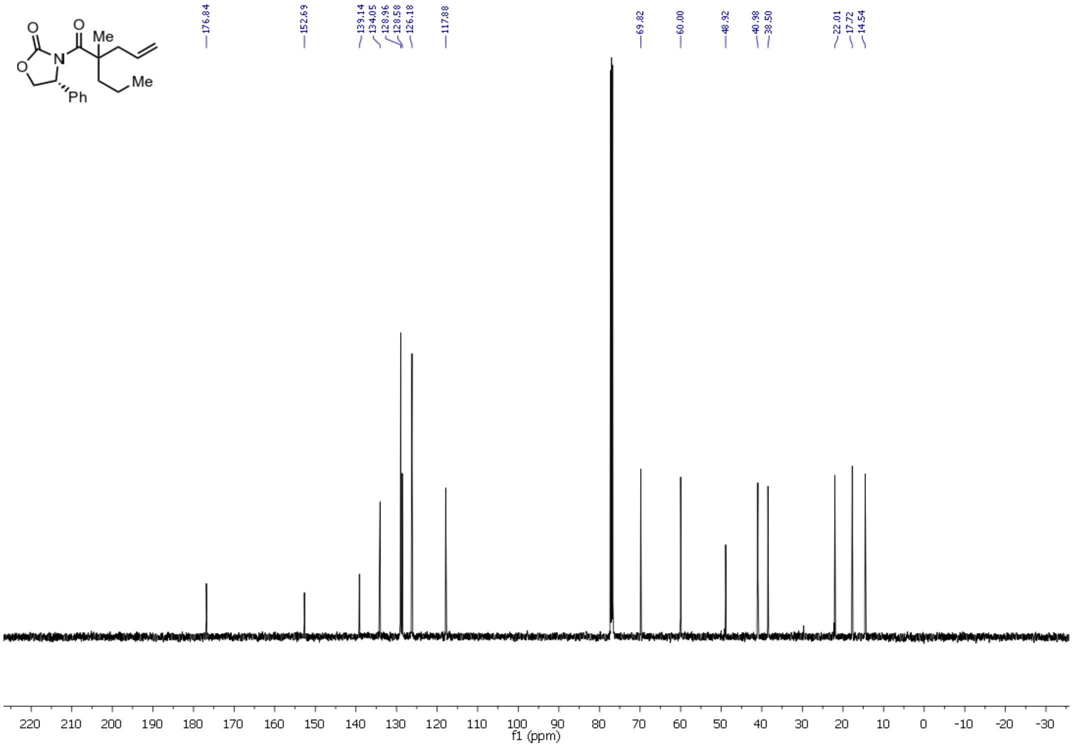

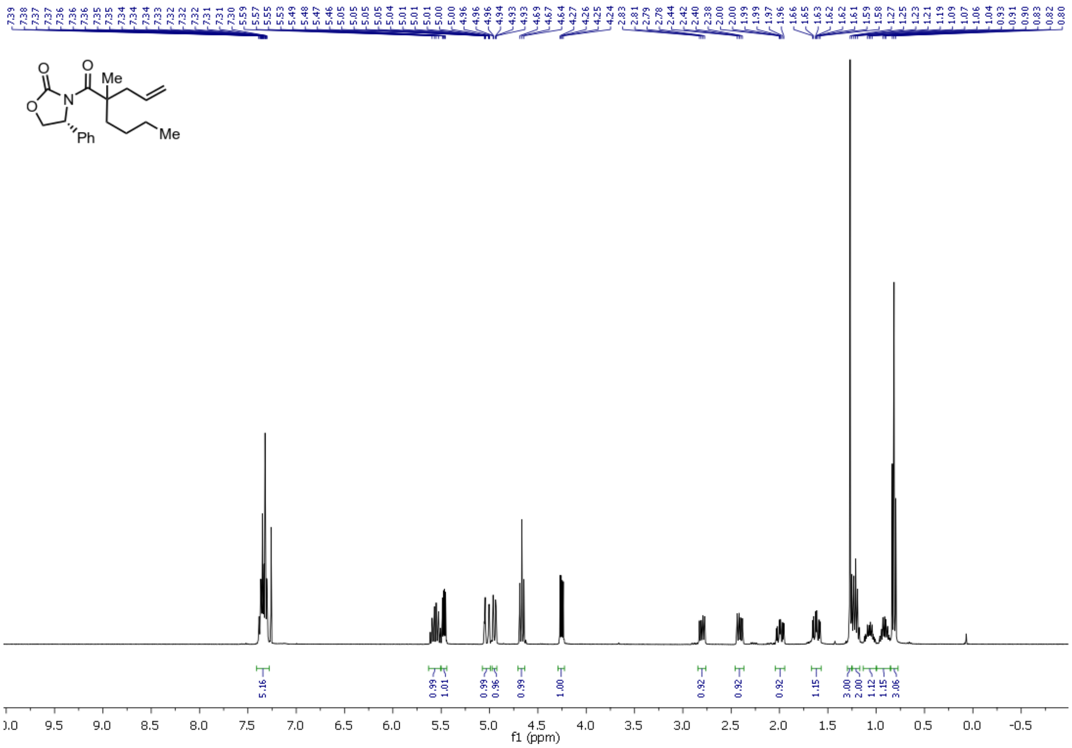

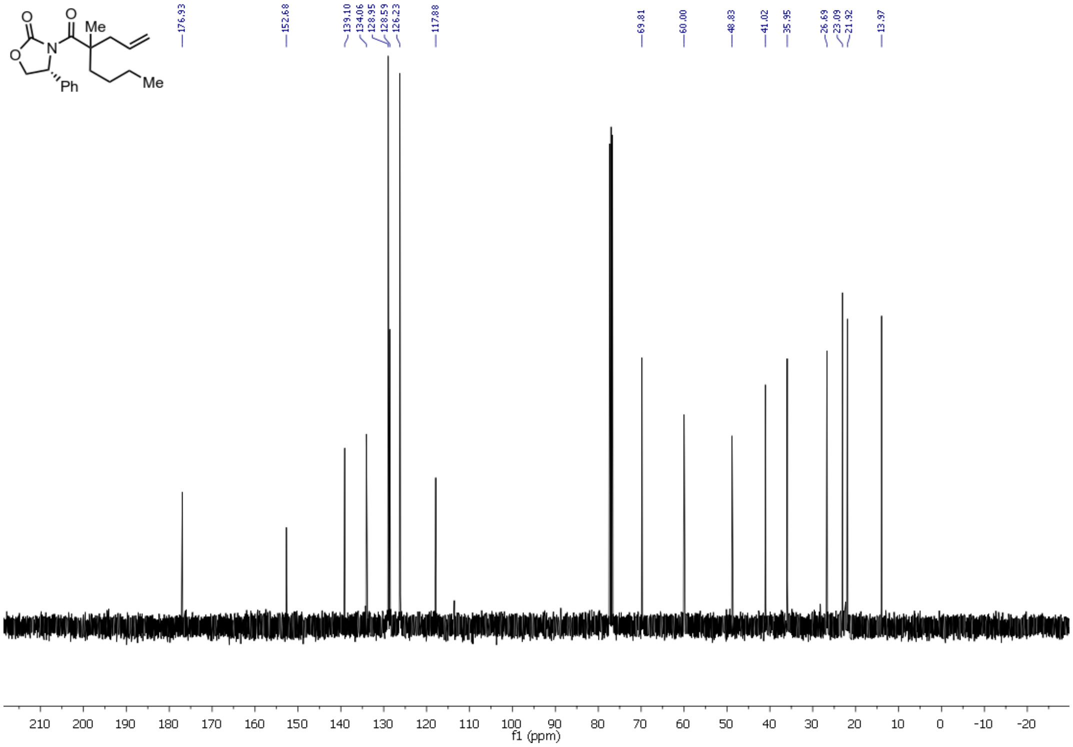

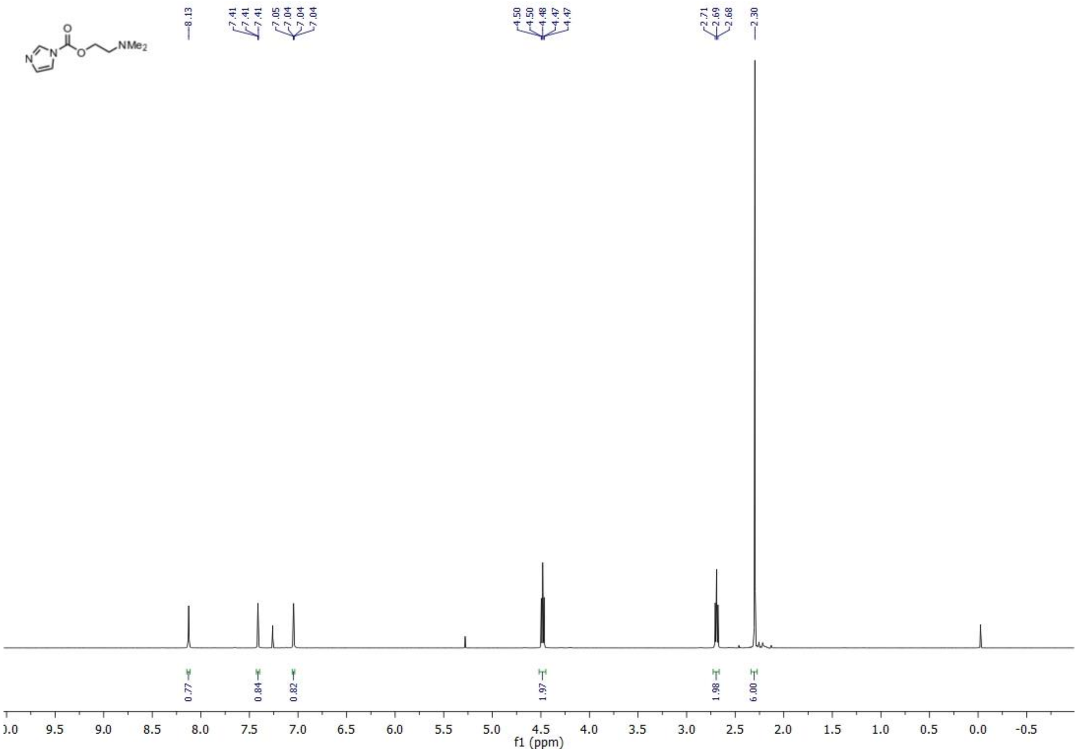
NMe2
8.13
7.41
7.41
7.41
7.05
7.04
7.04
7.04
4.50
4.50
4.48
4.47
4.47
2.71
2.69
2.68
2.30
0.77
0.84
0.82
1.97
1.98
6.00
f1 (ppm)

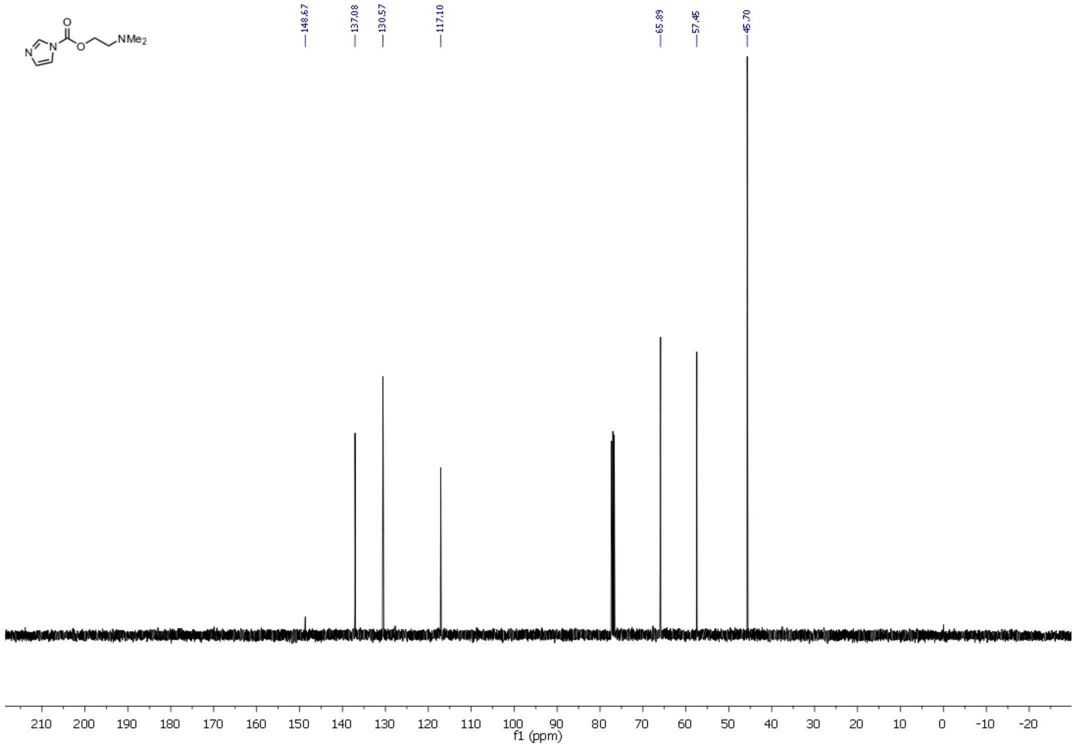
NMe2
148.67
137.08
130.57
117.10
65.89
57.45
45.70
f1 (ppm)

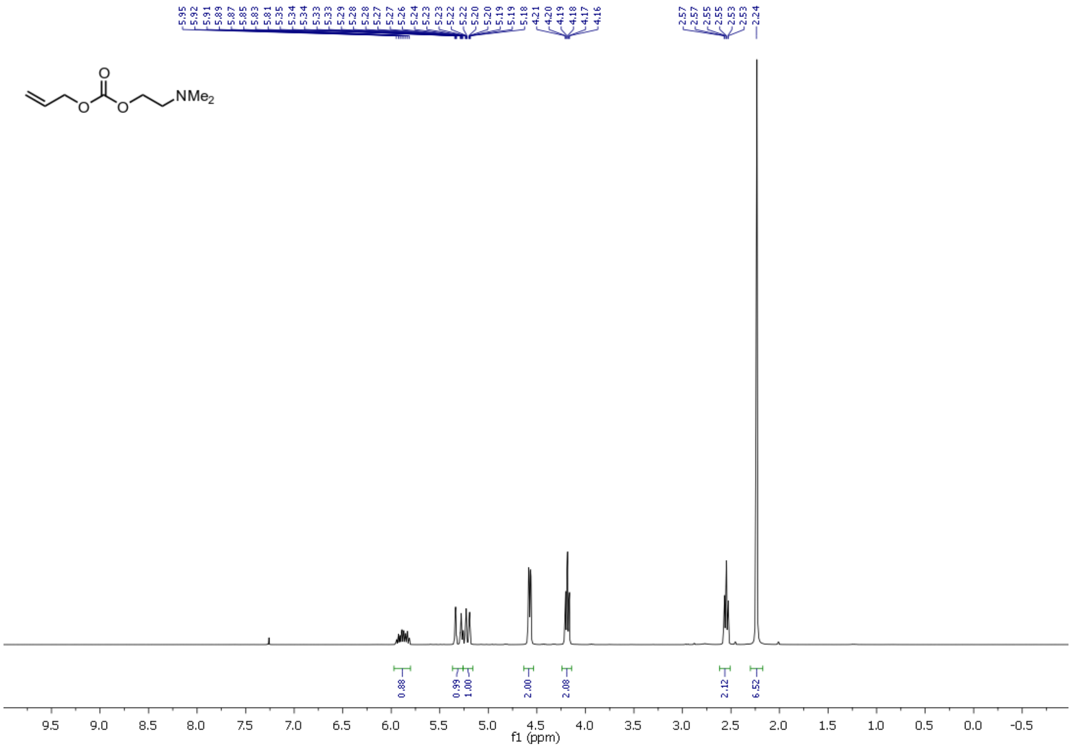

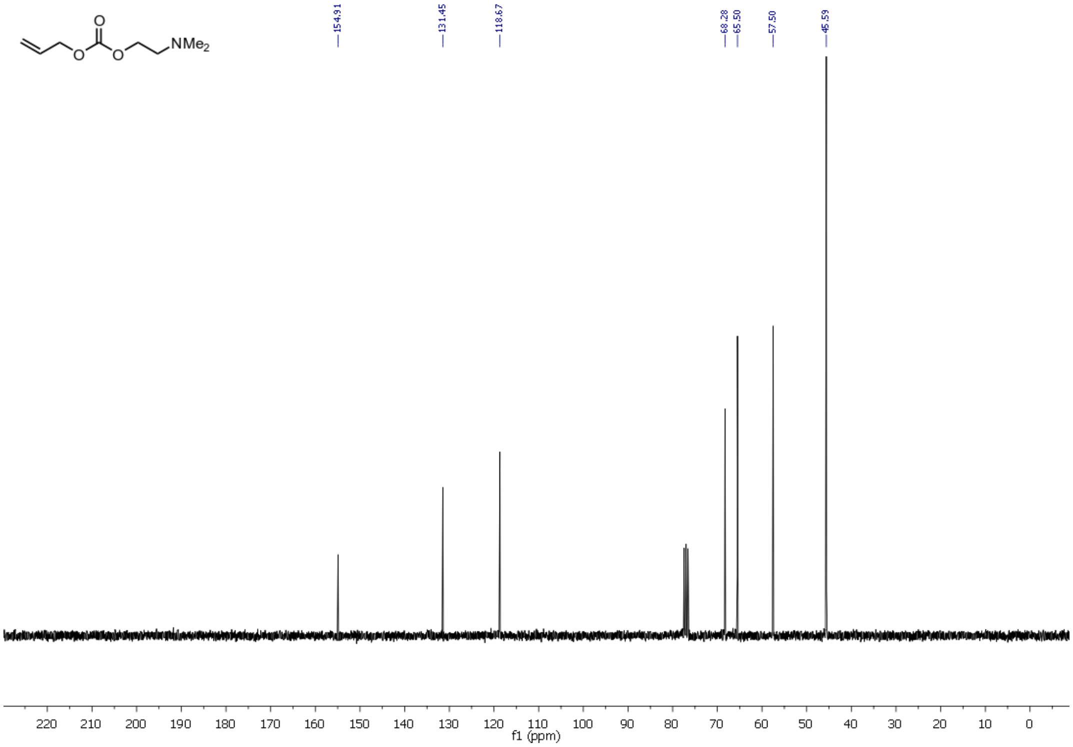

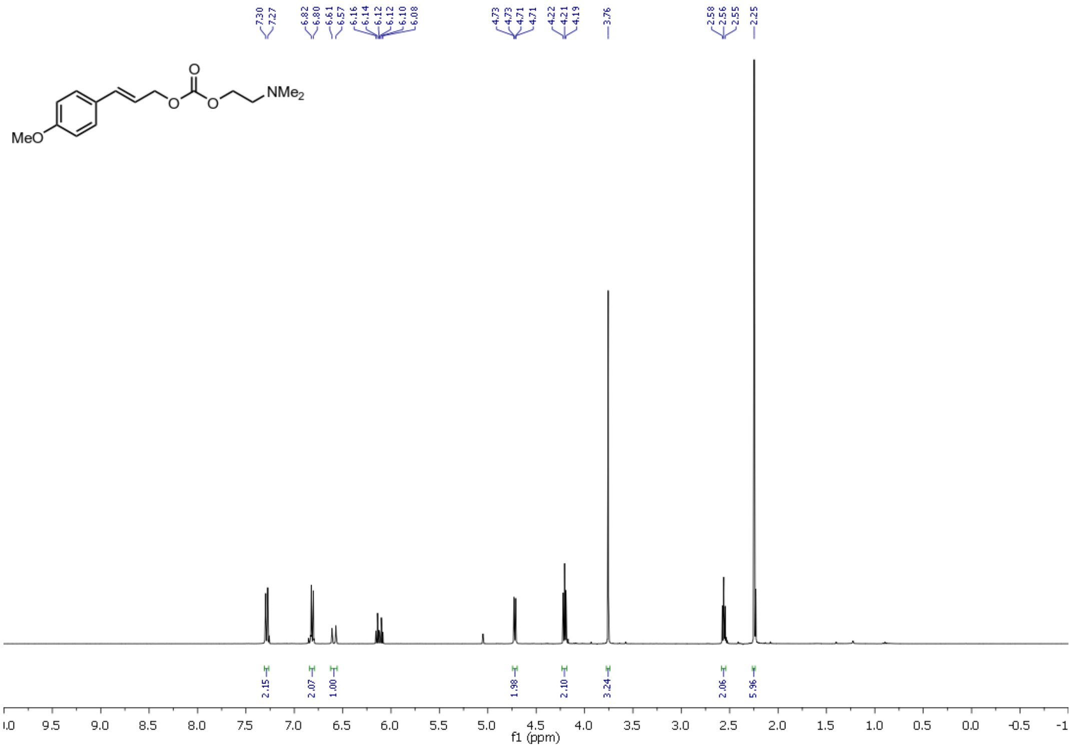

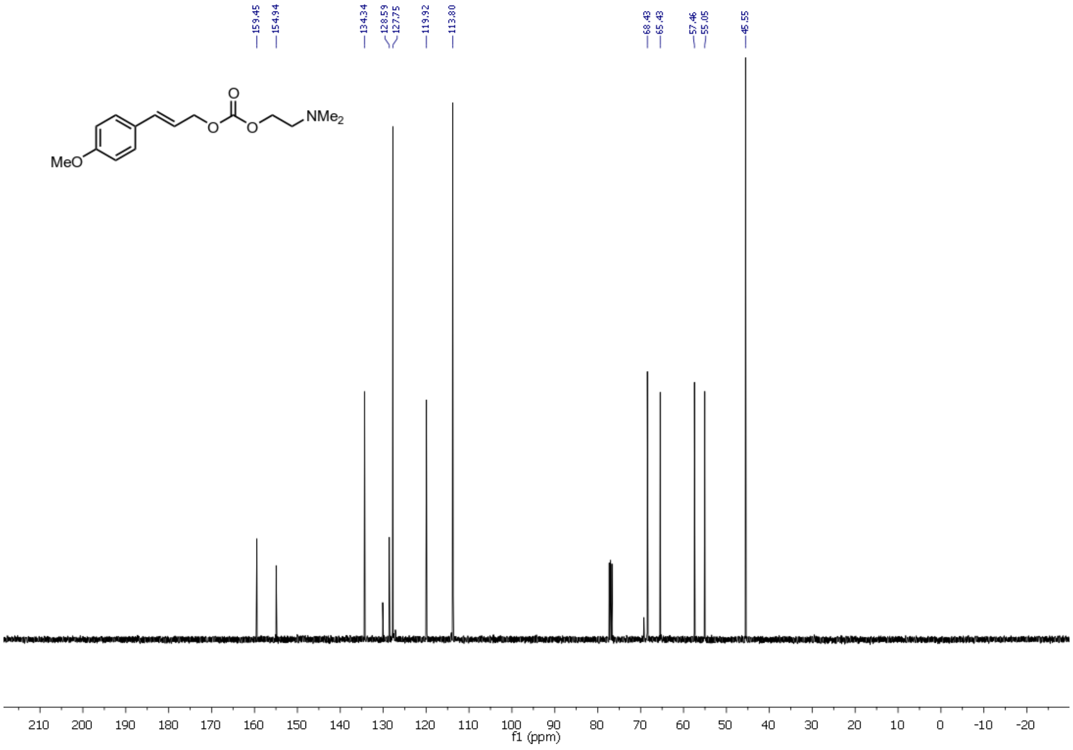

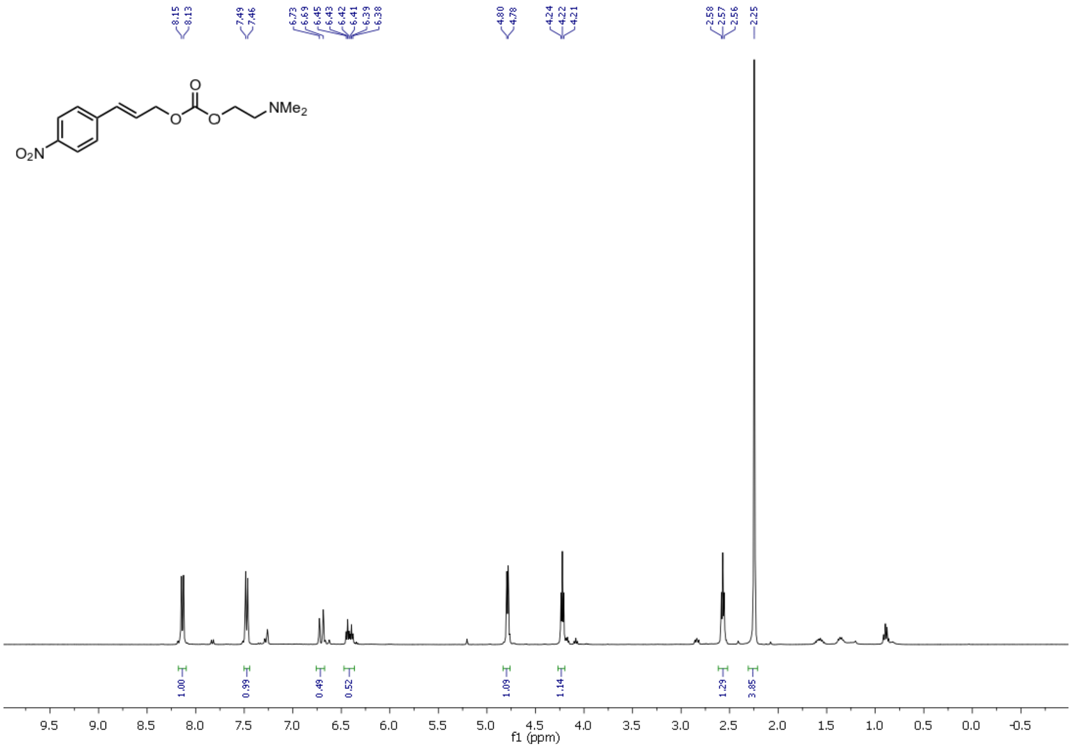
8.15
8.13
7.49
7.46
6.73
6.69
6.45
6.43
6.42
6.41
6.39
6.38
4.80
4.78
4.24
4.22
4.21
2.58
2.57
2.56
2.25
NMe2
O2N
O
O
O
1.00
0.99
0.49
0.52
1.09
1.14
1.29
3.85
f1 (ppm)

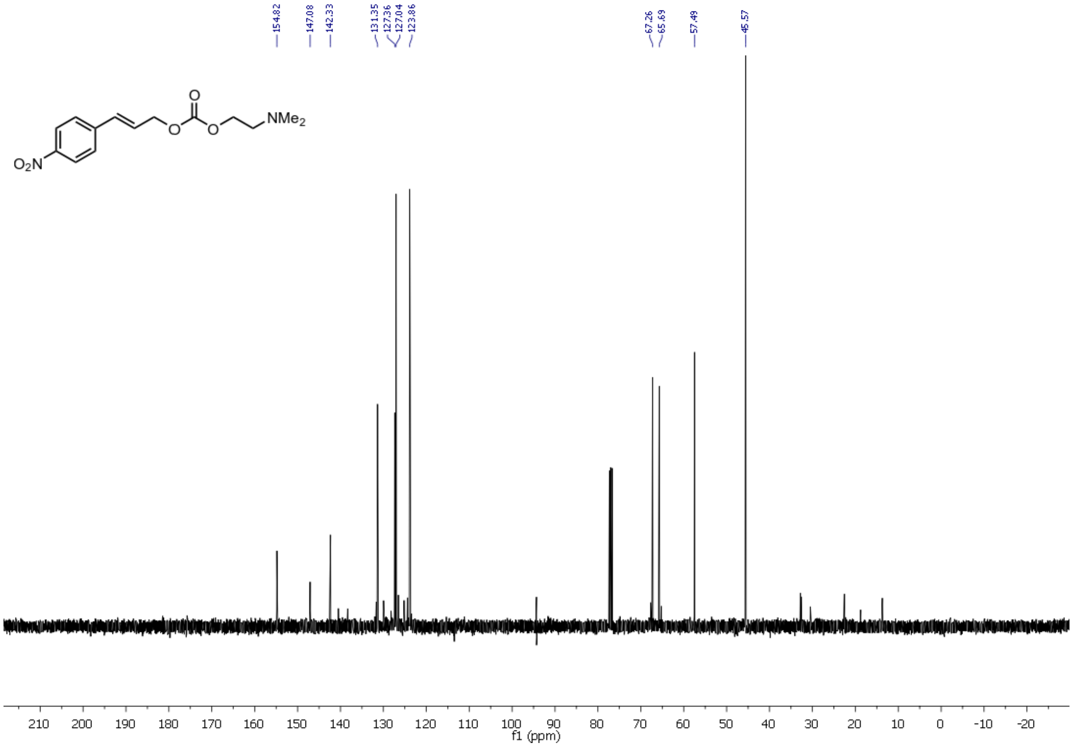
154.82
147.08
142.33
131.35
127.36
127.04
123.86
67.26
65.69
57.49
45.57
NMe2
O2N
O
O
O
f1 (ppm)

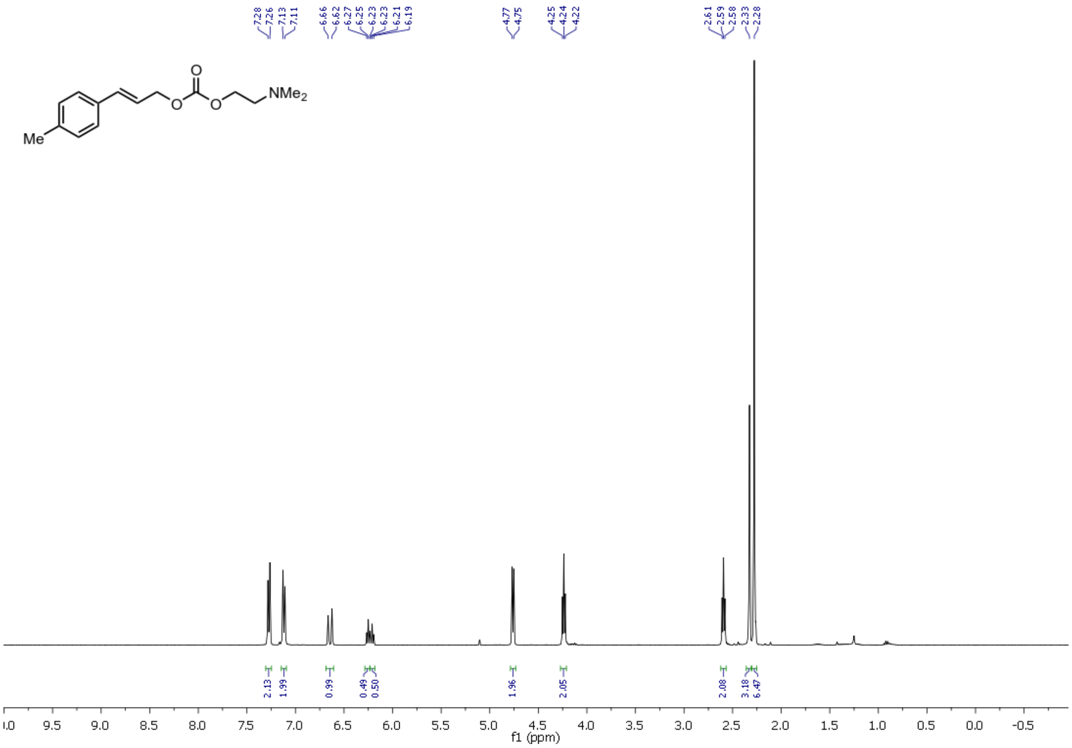

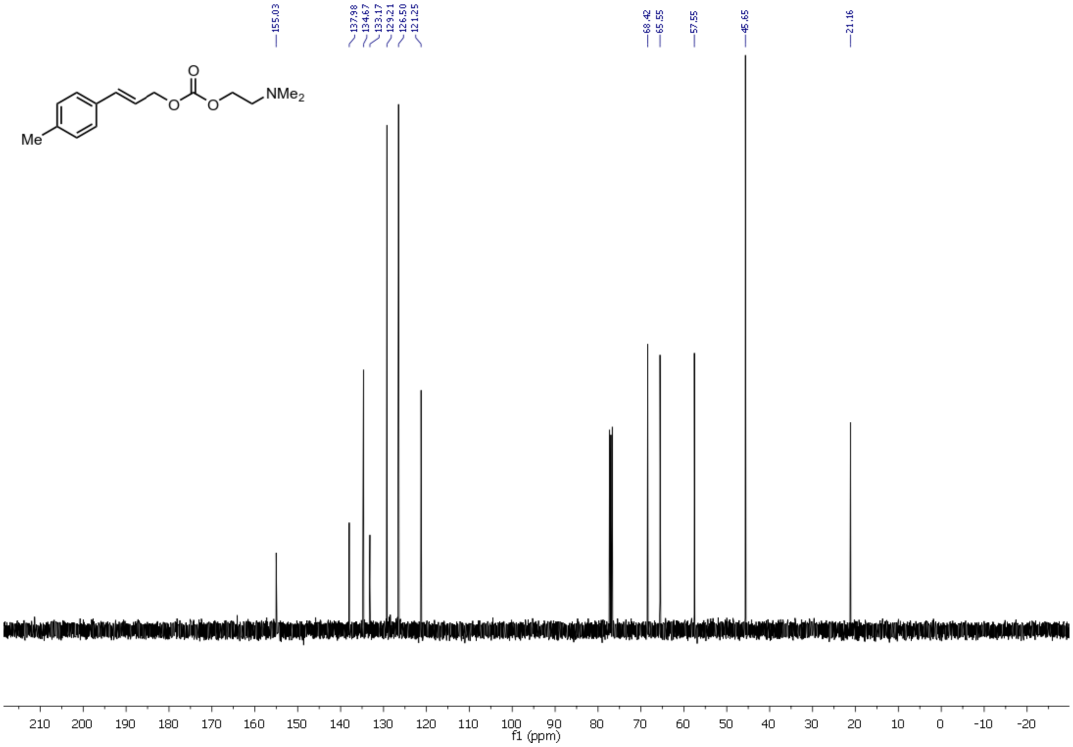

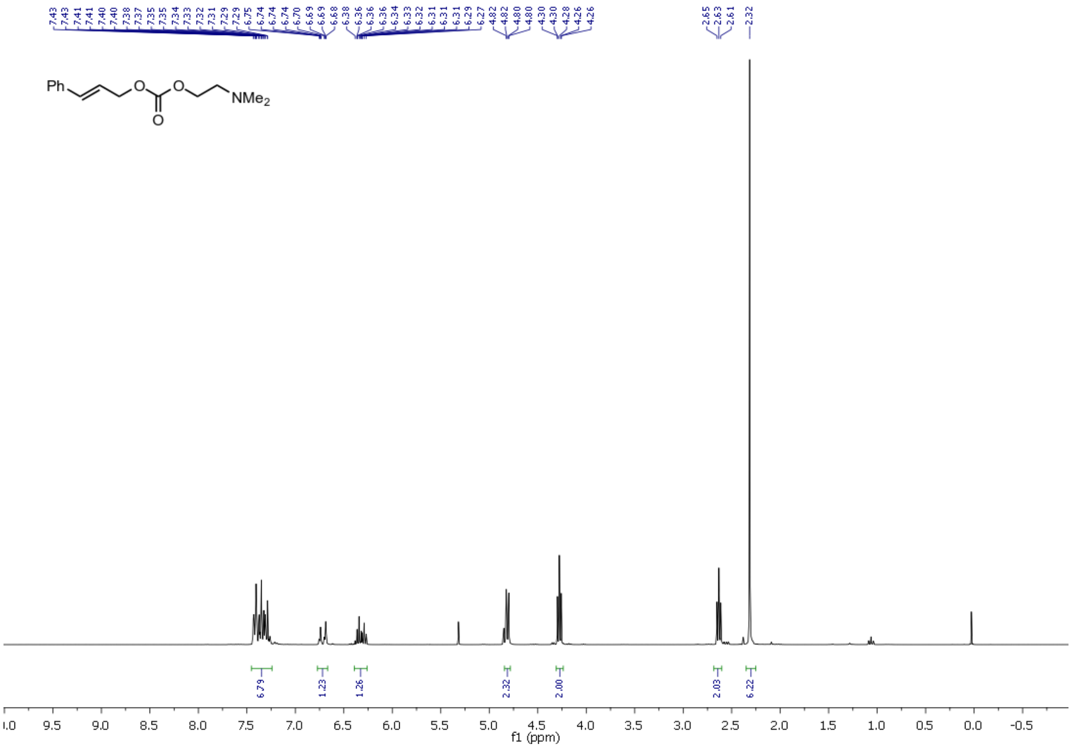

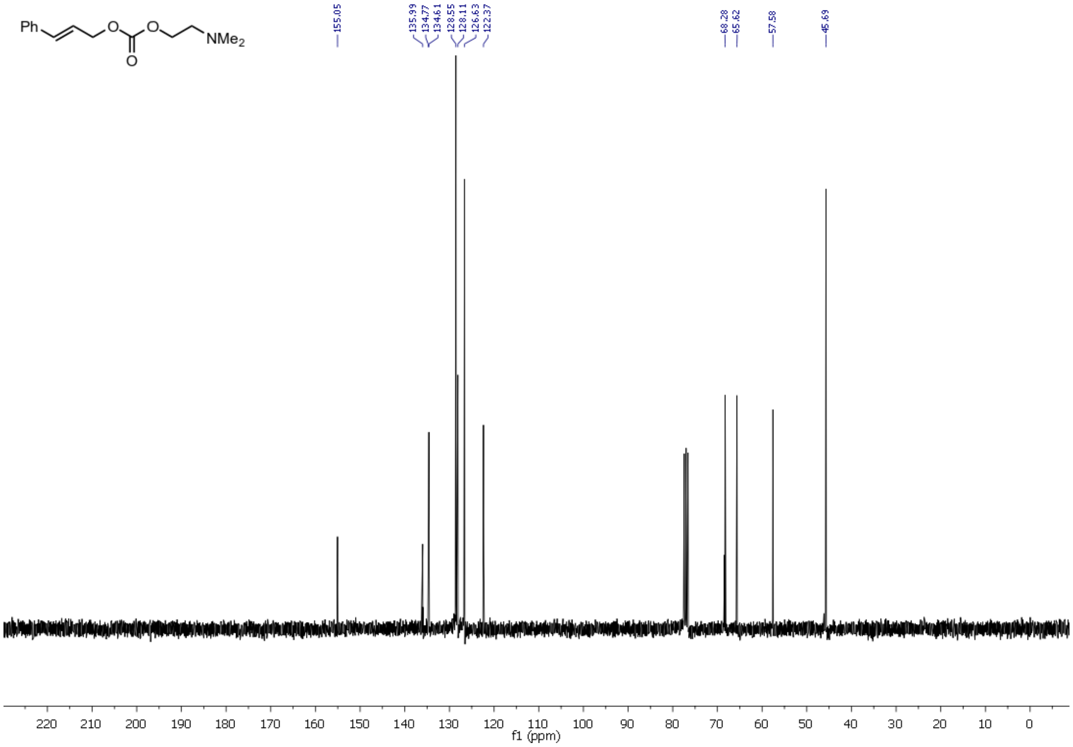

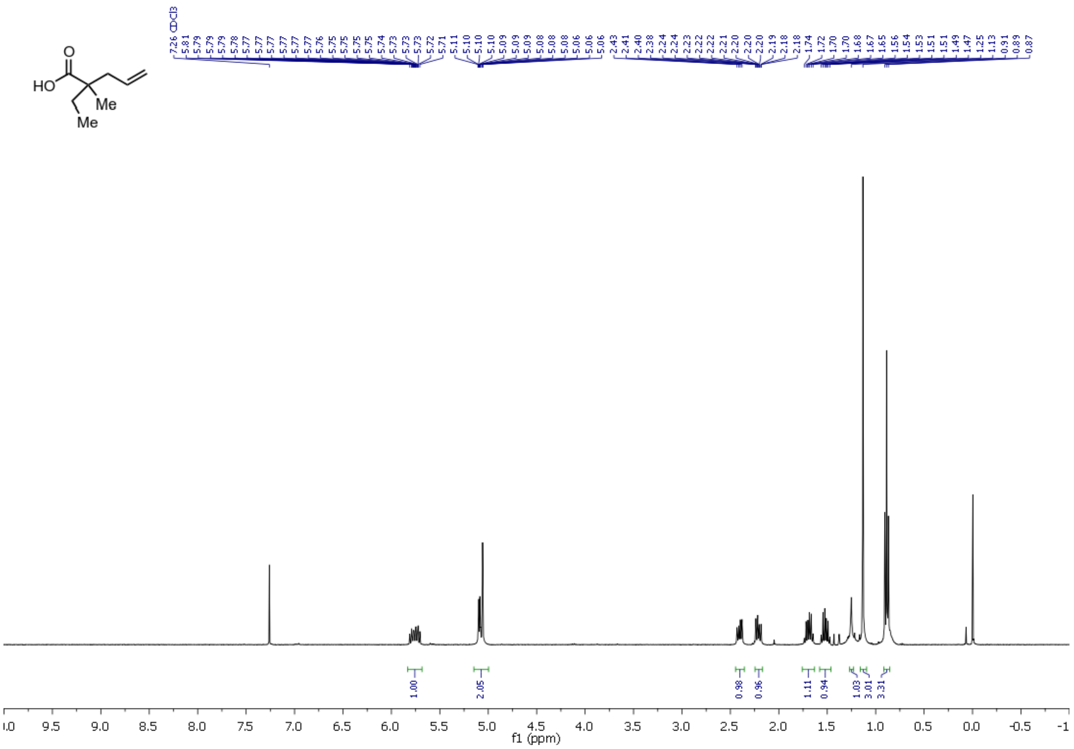
HO
O
Me
Me
f1 (ppm)

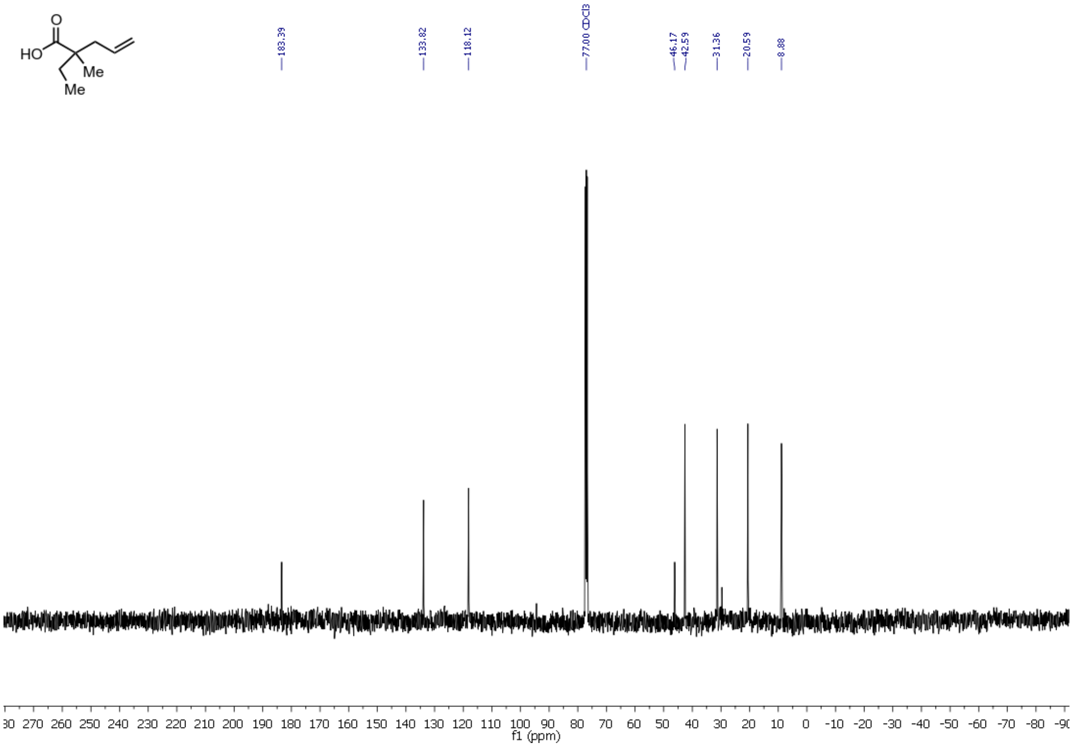
HO
O
Me
Me
183.39
133.82
118.12
77.00 CDCl3
46.17
42.59
31.36
20.59
8.88
f1 (ppm)
